# Furan-Site Bromination and Transformations of Fraxinellone as Insecticidal Agents Against *Mythimna separata* Walker

**DOI:** 10.1038/s41598-018-26747-0

**Published:** 2018-05-30

**Authors:** Qing-Miao Dong, Shuai Dong, Cheng Shen, Qing-Hao Cao, Ming-Yu Song, Qiu-Rui He, Xiao-Ling Wang, Xiao-Jun Yang, Jiang-Jiang Tang, Jin-Ming Gao

**Affiliations:** 1 Shaanxi Key Laboratory of Natural Products & Chemical Biology, College of Chemistry & Pharmacy, Northwest A&F University, Yangling, 712100 Shaanxi P.R. China; 2 Shaanxi Key Laboratory of Phytochemistry, Baoji University of Arts and Sciences, Baoji, 721007 Shaanxi P.R. China; 3 School of Chemistry & Chemical Engineering, Yanan University, Yanan, 716000 Shaanxi P.R. China

## Abstract

Furan ring of limoninoids is critical in exhibiting insecticidal activity. Herein, fraxinellone (**1**) was used as a template of furan-containing natural products and a series of its derivatives was synthesized by selective bromination in good yields on gram-scale and following Suzuki-Miyaura or Sonogashira coupling reactions in moderate to good yields. Bromination of limonin (**9**) was also accomplished without altering other functional groups in high yield. Furthermore, an evaluation of insecticidal activity against the instar larvae of *Mythimna separata* showed that derivatives **2**, **3b**, **3g**, **5a**, **5d** and **5h** displayed more potent insecticidal activity than **1** and toosendanin.

## Introduction

Nowadays, the wanton use of synthetic agrochemicals has resulted in problems such as pesticide accumulation in crops and resistance in pests’ resistance, which post great threat to food safety and human health. Therefore, development of new agrochemicals with new target sites is becoming rather urgent^1–4^. Natural products (NPs) play an important role in novel pesticide discovery for their unique sources and potential target sites^5–11^. Although many drugs are naturally occurring substances, natural product derivatives (NPDs) are often more necessary to improve their pharmacokinetic properties, exemplified as the number of NPDs is over 5 times than that of NPs in the new drugs from 1981 to 2014^12^. Generally, NPDs can be accessed through total synthesis^13–15^ and mutasynthesis^16–19^. In cases where a NP is readily available from the natural source, semisynthesis will be an attractive approach. Due to the complex scaffolds of NPs, semisynthesis requires highly selective transformations.

**Figure 1:**
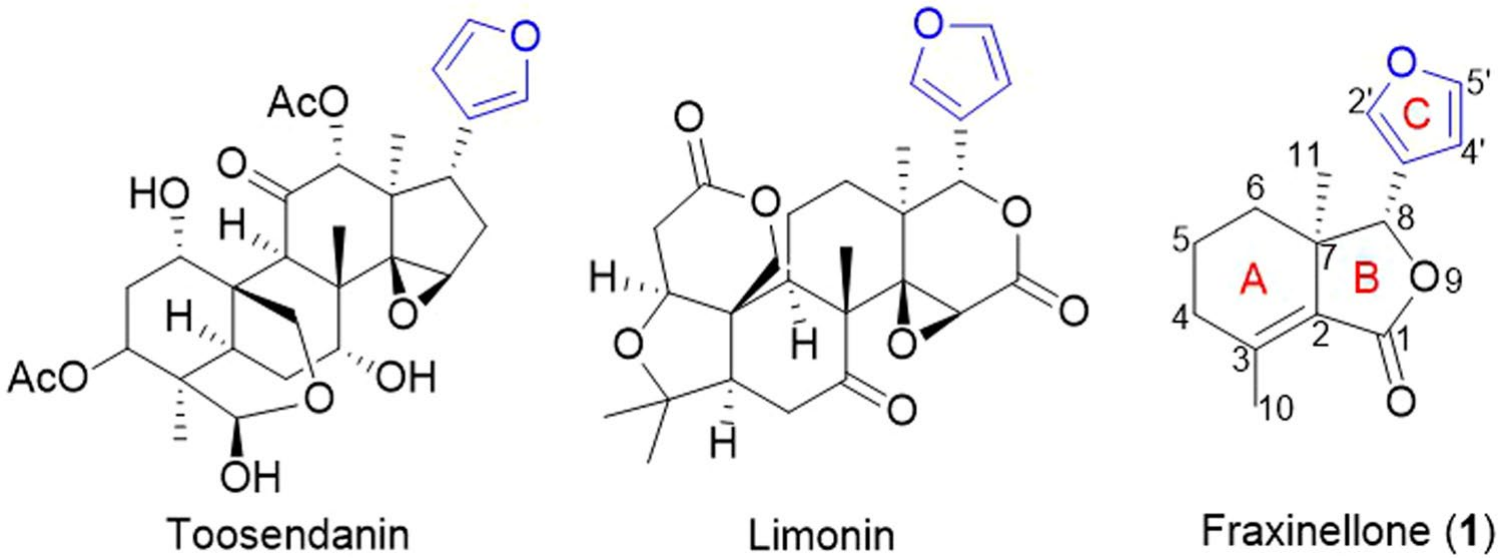
Representative furan-containing natural products: Toosendanin, Limonin and Fraxinellone (**1**).

Furan ring as an important pharmacophore is widely present in a variety of natural products (Fig. 1) exhibiting different bioactivities^8,20–24^. For example, toosendanin (Fig. 1), an allelochemical triterpenoid from the bark of the trees *Melia toosendan* and *M. azeduvach* (Meliaceae), exhibited potent antifeedant and growth inhibitory effects against armyworm *Mythimna separata* and cutworm *Peridroma saucia*^23,24^. Besides, limonin (Fig. 1), a highly oxygenated tetracyclic triterpene (enriched in citrus fruits), showed the insecticidal activity^25^, in which the furan ring seemed to be critical on exhibiting antifeedant activity and growth inhibitory activity against *C. Cucumerinum*^25^.

Fraxinellone (**1**, Fig. 1), a degraded limonoid^26^ mainly isolated from Meliaceae and Rutaceae plants, exhibits potential insecticidal activity^27,28^ and inhibits hepatic stellate cells (HSCs) activation through reducing CUG-binding protein 1 expression^29^. To enrich the structure diversity of **1**, semisynthetic derivatives have been synthesized by selectively altering the many functional groups present in **1**. The structure-activity relationships (SAR) regarding the C-4, C-10 positions of A-ring and the lactone of B-ring have been thoroughly investigated. In an elegant study, Xu group^30^ showed that the double bond between the C-2 and C-3 of **1** is not necessary for the insecticidal activity, whereas the lactone (B-ring) is vital motifs against instar larvae of *Mythimna separata*. Interestingly, they also reported that fraxinellone-based esters and hydrazones derivatives at C-4/C-10 position (A-ring) displayed higher insecticidal activity^31–33^. In contrast, the lack of chemical handle on the furan ring has limited the derivatives with the furan ring intact to only a few examples^34^. Since the importance has implicated the furan ring, such derivatives would be useful in elucidating additional SAR.

**Figure 2:**
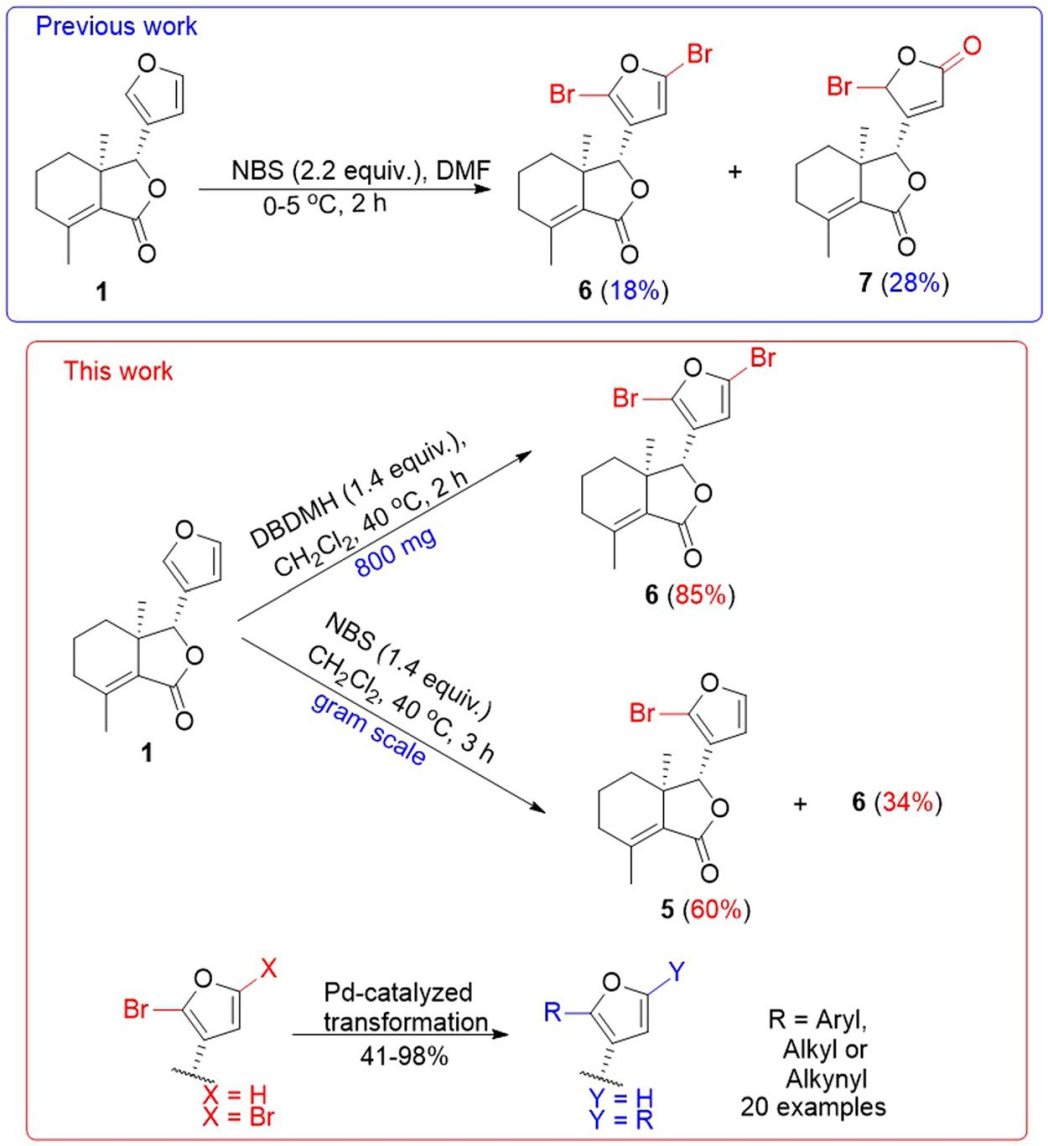
Improved selective bromination of **1**.

The modification of aromatic functionalities of NPs could be accomplished by introducing a halide using enzymatic^17,18,35^ or chemical methods^36^, followed by further diversification using various palladium-catalyzed transformations^20^. The furan ring of **1** was previously halogenated selectively at the C-2′ and C-5′ position using *N*-bromosuccinimide (NBS) or *N*-chloromosuccinimide (NCS), however, the reaction conditions only gave dihalofraxinellone **6** and lactone **7** in very low yields^34^ (less than 30% in total) (Fig. 2). Thus, the lack of selective halogenation of the furan ring of NPs had limited the synthese of their derivatives.

In our continuing endeavor to find more active natural product-based insecticidal hits^37–44^ and in order to improve the selectivity of halogenation and enrich the chemical diversity of furan-containing NPs, we herein report the more selective brominations and further palladium-catalyzed transformations on furan rings of fraxinellone (**1**) and its reduced derivative **2**, and their insecticidal activity against *M. separata* Walker.

## Results and Discussion

### Chemistry

**Figure 3:**
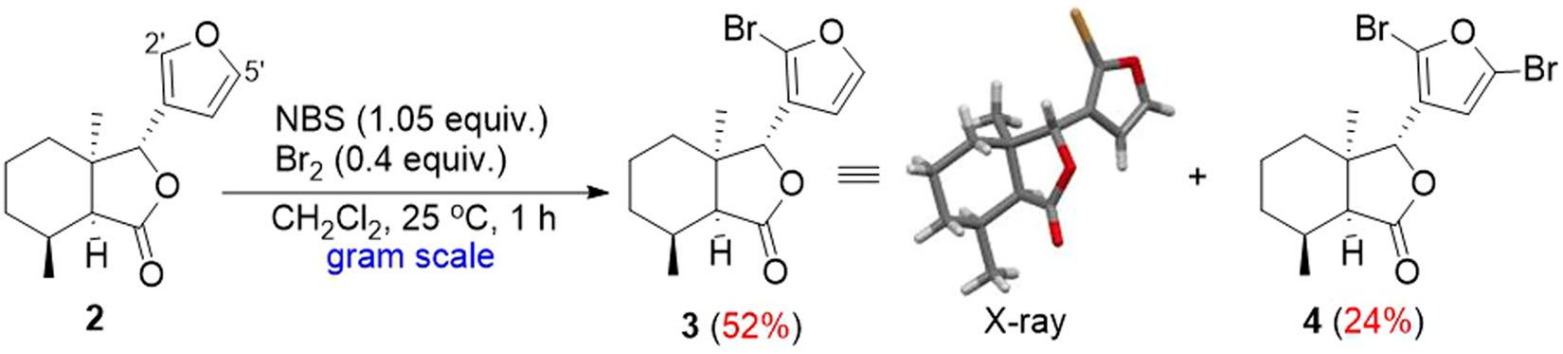
Bromination of **2**. CCDC number of compound **3** (X-ray) is 1549252.

The furan ring of fraxinellone (**1**) was previously brominated at the C-2′ and C-5′ positions using NBS (Fig. 2)^34^; however, these reaction conditions produced either low or highly variable yields. In order to optimize the reaction conditions to obtain the monobromofraxinellone or dibromofraxinellone selectively in a higher yield, a reduced fraxinellone (**2**) was used as the starting material for excluding the possible effect of C-2 and C-3 double bonds in the NBS bromination reaction of **1**. Reaction temperature and the loading amount of NBS were investigated (Table S1), but it was found that these reactions not only required long reaction times but also produced target production **3** or **4** (Fig. 3) in low yield. Considering that the Br^+^ mechanism was involved in the bromination and Br_2_ of low concentration was continuously released with NBS, we tested Br_2_ as the brominating reagent directly and the combination of NBS (as brominating reagent) and Br_2_ (as catalyst) in the reaction, respectively. Finally, studies revealed that 0.4 eq of Br_2_ with 1.05 eq of NBS under room temperature was optimal. In that condition, monobromination product **3** was produced in 52% yield and dibromides **4** was obtained in 24% yield (Fig. 3). The steric configuration of **3** was also confirmed by X-ray crystallography.

**Figure 4:**
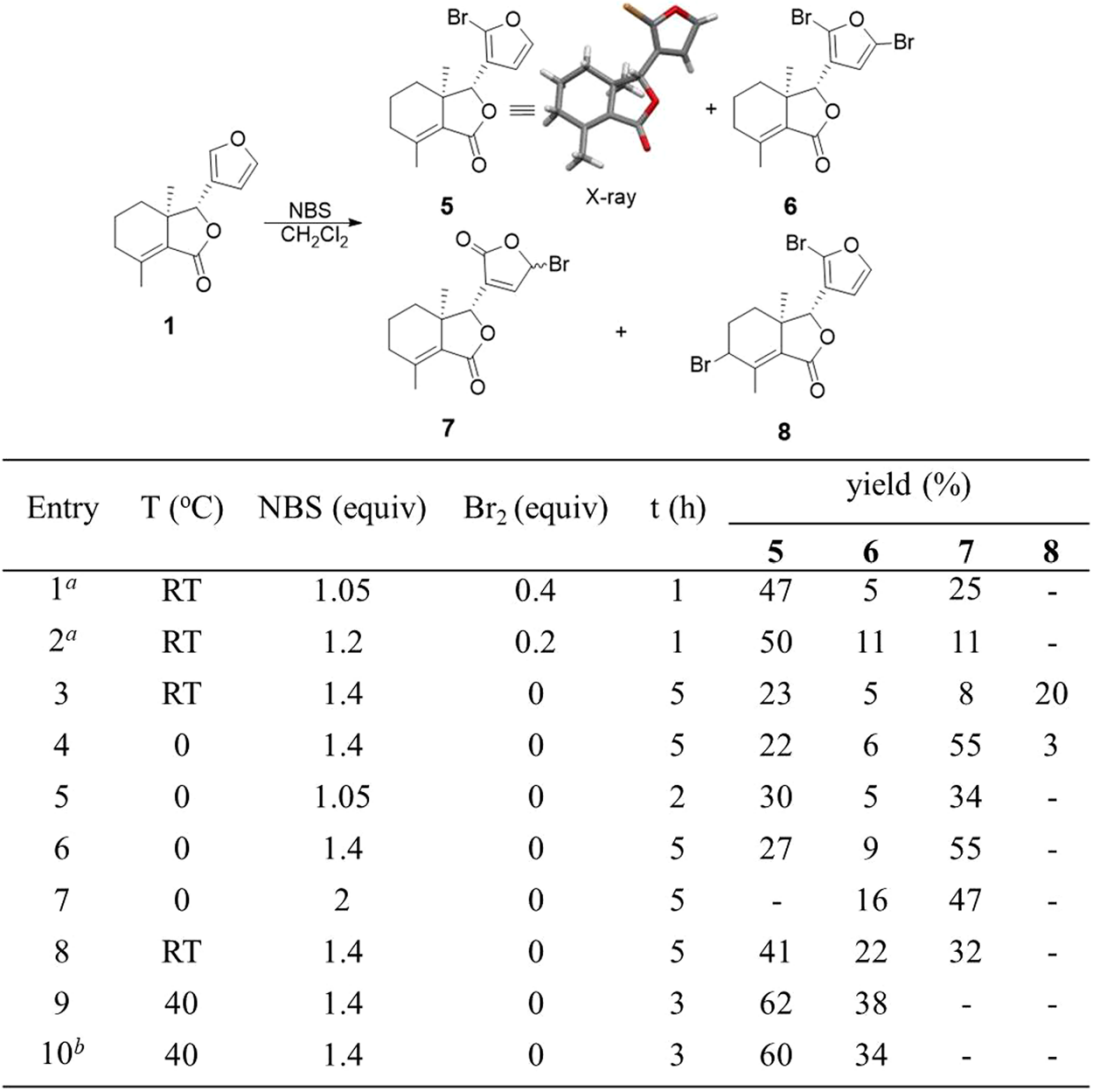
Bromination of Fraxinellone (**1**). Reactions were performed using 0.02 mmol of **1** in CH_2_Cl_2_. Yield was determined by ^1^H NMR analysis of the reaction mixture in 0.6 mL CDCl_3_ (See the Figs S3–S5 in the Supporting Information). Entries 1–4 were carried out under CFL (household compact fluorescent lamp, 28 W), and entries 5–10 were carried out in the dark; The CCDC number of compound **5** (X-ray) is 1549253; ^*a*^Isolated yield; ^*b*^1.1 g of **1** was carried out.

Using the optimal bromination condition of **2**, the bromination of fraxinellone (**1**) was then examined (Fig. 4, entries 1 and 2), and three main products (**5**, **6** and **7**) were separated and analyzed, in which the lactone **7** might be the product of the reaction between H_2_O and Br onium ion (more likely) or the hydrolysis of **6**. Interestingly, NBS as the only brominating reagent (entries 3 and 4), the reactions also could complete in 5 hours, while the byproduct **8** was produced naturally under the free radical mechanism from **5**. In order to obtain the target productions **5** and **6** selectively, the reactions were then carried out at 0 °C in the dark. However, compound **8** could be avoided but **7** was obtained in higher yields than that at room temperature whatever the loading of NBS was high or low (entries 5–7). For example, when 1.4 equivalent NBS was used, the lactone **7** was produced in 55% yield. Based on the experimental results, we proposed temperature may be related to the production of the lactone **7**. Next, the influence of temperature was investigated. To our delight, the yields of **7** decreases with the increasing of the temperature (entries 8-9). Notably, when the temperature was increased to 40 °C and 1.4 equivalent NBS was used alone, the monobromide **5** (62%) and dibromides **6** (38%) were obtained in nearly quantitative yield (entry 9).

**Figure 5:**
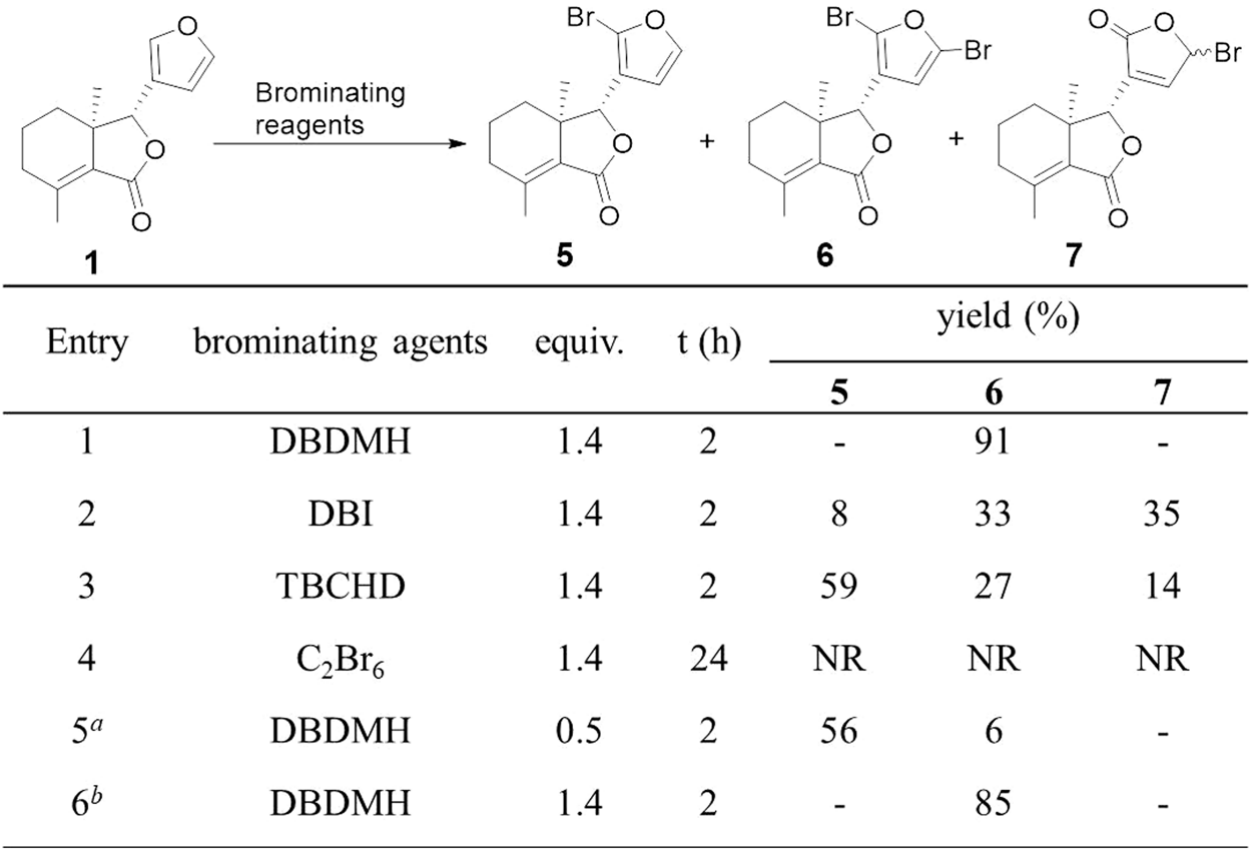
Bromination of **1** with different reagents. Reactions were performed using 0.02 mmol of **1** in CH_2_Cl_2_ at 40 °C in the dark. Yield was determined by ^1^H NMR analysis of the reaction mixture in 0.6 mL CDCl_3_ (See the Figs S3 and S6 in the Supporting Information); DBDMH: 1,3-Dibromo-5,5-dimethylhy-dantoin; DBI: 1,3-Dibromo-1,3,5-triazine-2,4,6-trione; TBCHD: 2,4,4,6-Tetrabromo-2,5-cyclohex-adien one. NR: No reaction; ^*a*^65% conversion of **1**; ^*b*^800 mg of **1** was carried out.

Next, other four typical brominating reagents (DBDMH, DBI, TBCHD and C_2_Br_6_) were further chosen to investigate the selective bromination of furan ring of fraxinellone (**1**), and the results are shown in Fig. 5. When the reaction was carried out with 1.4 equivalent of 1,3-dibromo-5,5-dimethylhydantoin (DBDMH) at 40 °C in 2 h, to our delight, the dibromides **6** was obtained in excellent yield (91%) as the sole product. 0.5 equivalent of DBDMH gave the monobromide **5** in 56% yield and 35% of **1** recycled. Other reagents, such as 1,3-dibromo-1,3,5-triazine-2,4,6-trione (DBI) or 2,4,4,6-tetrabromo-2,5-cyclohexadienone (TBCHD), gave the mixture of at least three products, or no product was converted with C_2_Br_6_.

**Figure 6:**
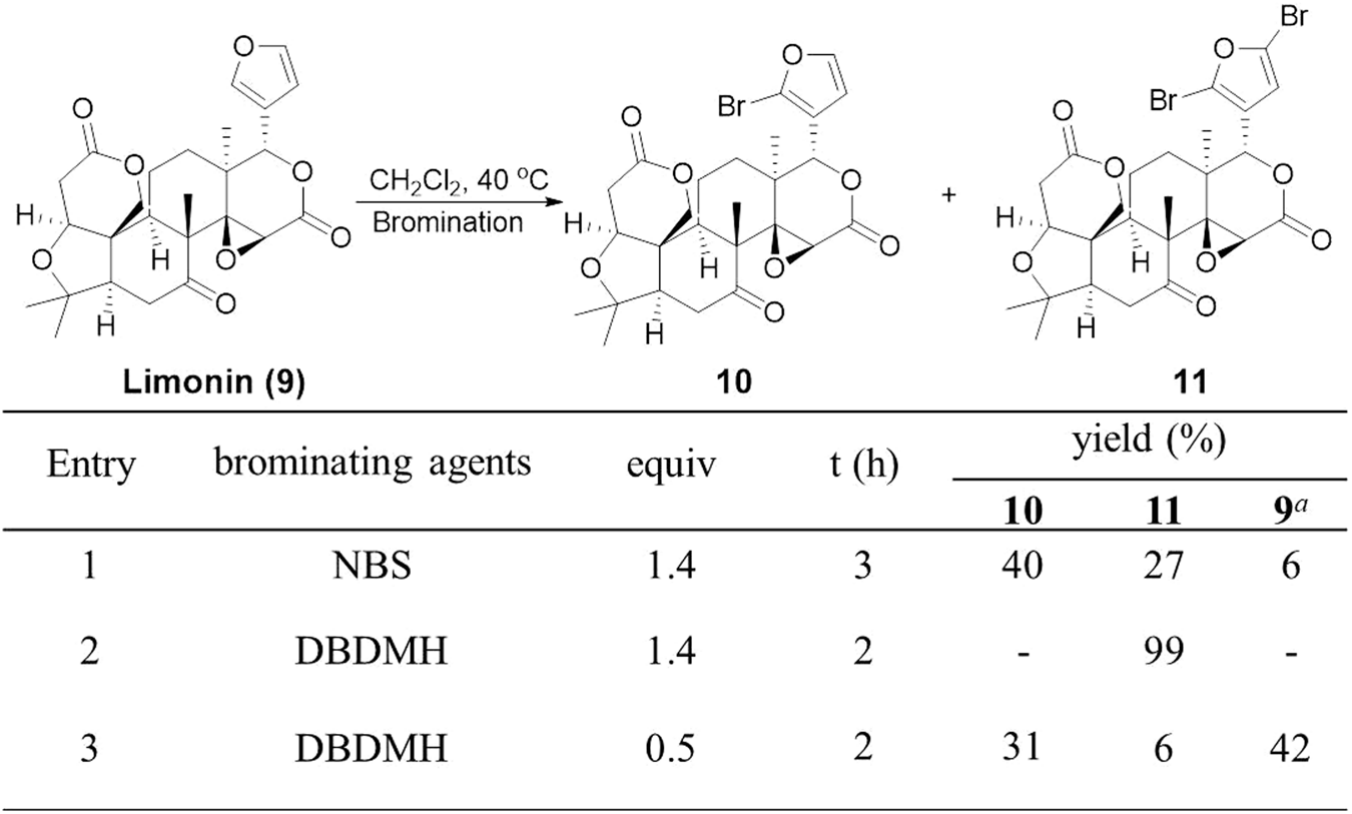
Bromination of Limonin (**9**). Reactions were performed using 0.02 mmol of **10** in CH_2_Cl_2_ at 40 °C in the dark. Yield was determined by ^1^H NMR analysis of the reaction mixture in 0.6 mL CDCl_3_ (See the Figs S7 and S8 in the Supporting Information); ^*a*^Percent of **9** recovered.

To extend the bromination conditions (entry 9 in Fig. 4 and entry 1 in Fig. 5) to more complex NPs, the bromination of limonin (**9**) was also investigated in Fig. 6. Monobromofuran **10** and dibromofuran **11** of limonin were both obtained in similar yields as above. Notably, when the reaction was carried out with 1.4 equivalent of DBDMH, an excellent yield (99%) of **11** was obtained (entry 2). This result implied that the bromination conditions may be applied to the synthesis of biological probes using other furan-containing NPs.

Having optimized the conditions to selectively introduce bromide(s) on the furan of **1** and **2**, next our attention turned to exploring the palladium-catalyzed transformations using Suzuki-Miyaura and Sonogashira couplings. All the intermediates (**3**, **4**, **5** and **6**) can be prepared in gram-scale. **2** with NBS (1.05 equiv.) and Br_2_ (0.4 equiv.) at room temperature for 1 h afforded **3** (52%) and **4** (24%), respectively (entry 12 in Table S1). **1** with NBS at 40 °C for 3 h gave **5** (60%) and **6** (35%), respectively (entry 10 in Fig. 4). 6 (85%) was obtained by reacting with DBDMH (1.4 equiv.) at 40 °C for 2 h (entry 6 in Fig. 5).

**Figure 7:**
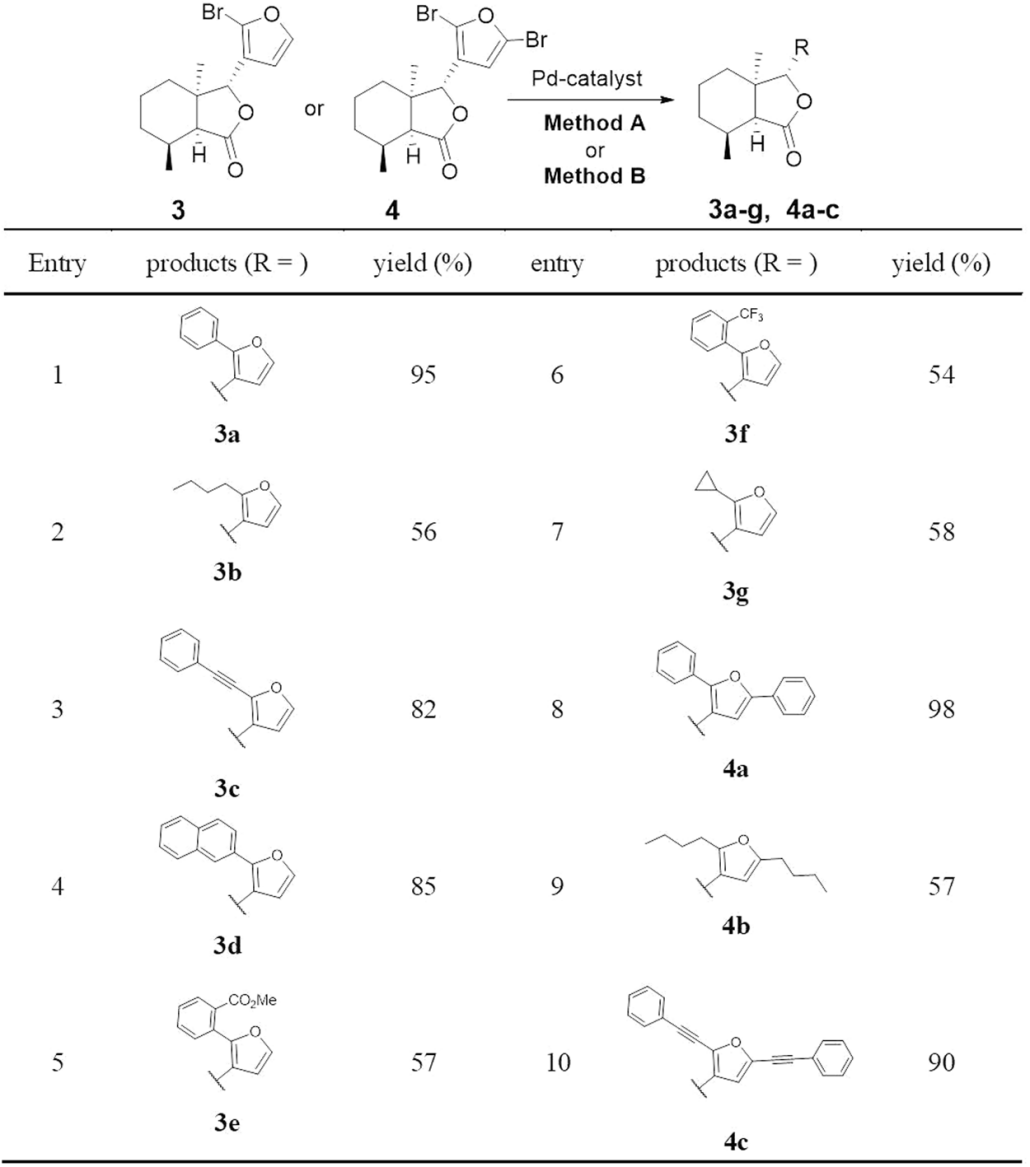
Palladium-Catalyzed Coupling Reactions of **3** and** 4**. Reaction conditions: Method A (Suzuki-Miyaura coupling for Entry 1, 2, 4–9): RB(OH)_2_ (2 or 4 equiv.), Pd_2_dba_2_ (0.04 equiv.), XPhos (0.16 equiv.), K_3_PO_4_ (3 equiv.), PhMe, 60 °C, 16 h; Method B (Sonogashira coupling for Entry 3, 10): PdCl_2_(PPh_3_)_2_ (0.05 equiv), CuI (0.1 equiv.), THF/Et_3_N (1:1), phenylethyne (2 or 4 equiv.), 60 °C, 16 h; Isolated yield.

**Figure 8:**
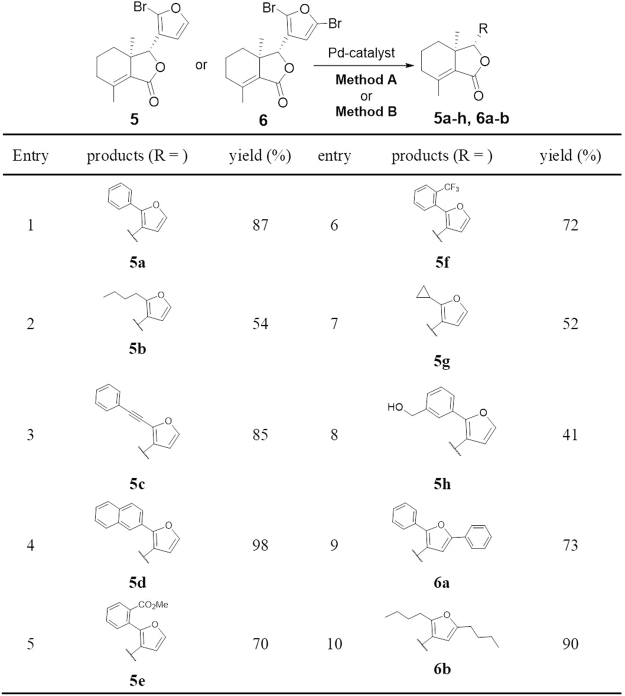
Palladium-Catalyzed Coupling Reactions of **5** and **6**. Reaction conditions: Method A (Suzuki-Miyaura coupling for Entry 1, 2, 4–9): RB(OH)_2_ (2 or 4 equiv.), Pd_2_dba_2_ (0.04 equiv.), XPhos (0.16 equiv.), K_3_PO_4_ (3 equiv.), PhMe, 60 °C, 16 h; Method B (Sonogashira coupling for Entry 3, 10): PdCl_2_(PPh_3_)_2_ (0.05 equiv), CuI (0.1 equiv.), THF/Et_3_N (1:1), phenylethyne (2 or 4 equiv.), 60 °C, 16 h; Isolated yield.

With bromofraxinellone derivatives **3**–**6** in hand, a variety of alkyl, aryl and alkynyl substituents were appended to the C-2′ and C-5′ position in **1** and **2** through palladium-catalyzed coupling reactions (Figs 7 and 8). For Suzuki-Miyaura coupling reaction of **3** and **4** as shown in Fig. 7, phenyl and naphthyl boronic acids typically produced good to excellent yields (**3a** 95%, **3d** 85%, **4a** 98%). In addition, aliphatic and phenyl rings bearing electron-withdrawing substituents produced fair yields (**3b** 56%, **3g** 58%, **4b** 57%, **3e** 57%, **3f** 54%). For Sonogashira coupling reaction, the phenylethynyl group was appended to furan ring of **1** to afford **3c** (82%) and **4c** (90%) in good to excellent yields. Similarly, coupling products of **5** and **6** were obtained through Suzuki-Miyaura and Sonogashira reactions (Fig. 8). In the case of *meta*-hydroxymethylphenyl boric acid, the expected product **5 h** was produced in 41% yield. Besides, representative compounds showed water stability at the 1.0 mM of concentration stayed for at least 3 days in PBS (pH 7.4) with HPLC analysis (shown in Fig. S9).

### Insecticidal activity evaluation

**Figure 9:**
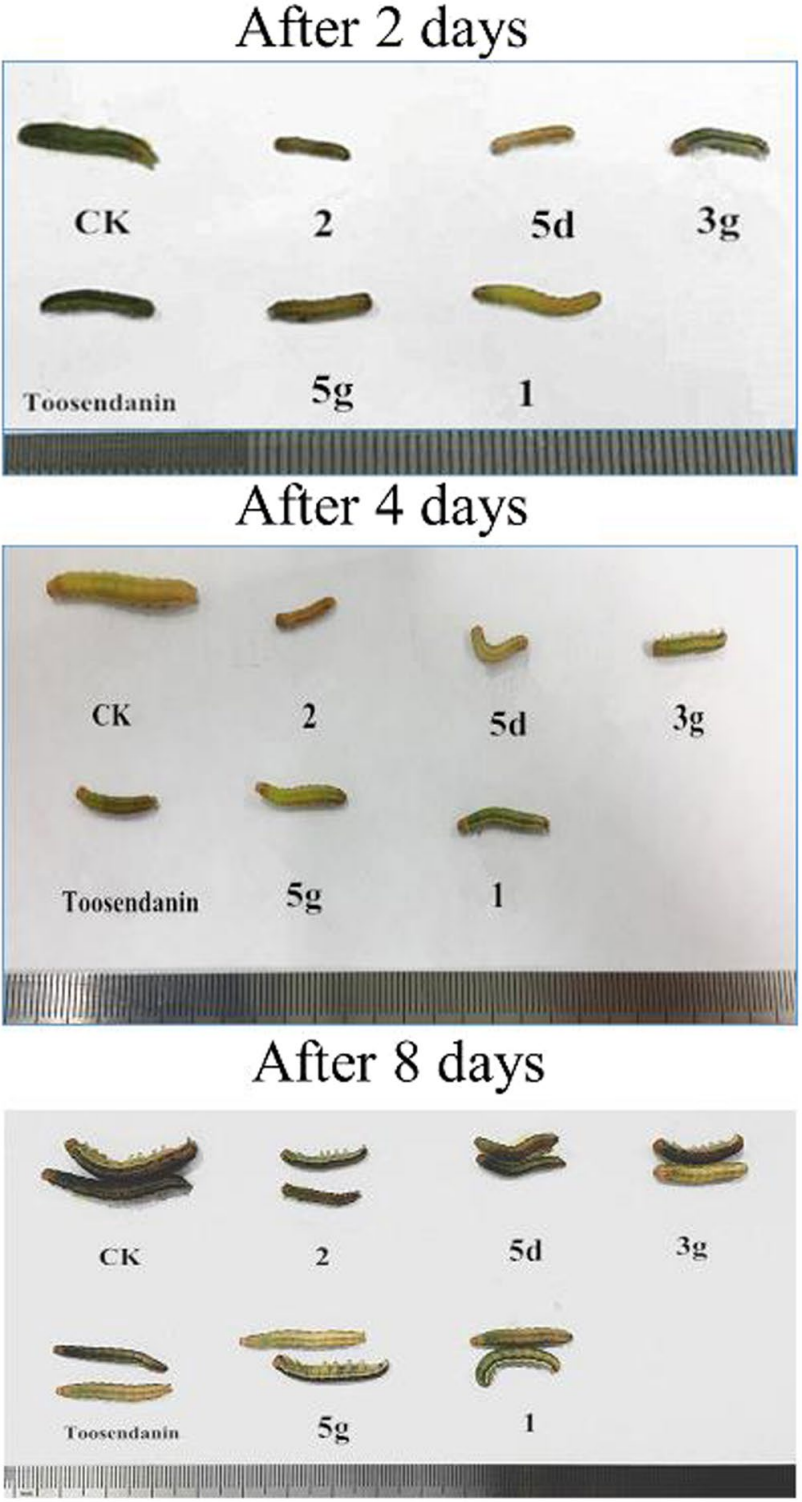
Representative growth inhibitory larvae pictures of derivatives **1, 2, 3g, 5d, 5g** (CK = blank control group, toosendanin = positive control group) after 2 days, 4 days and 8 days.

**Figure 10:**
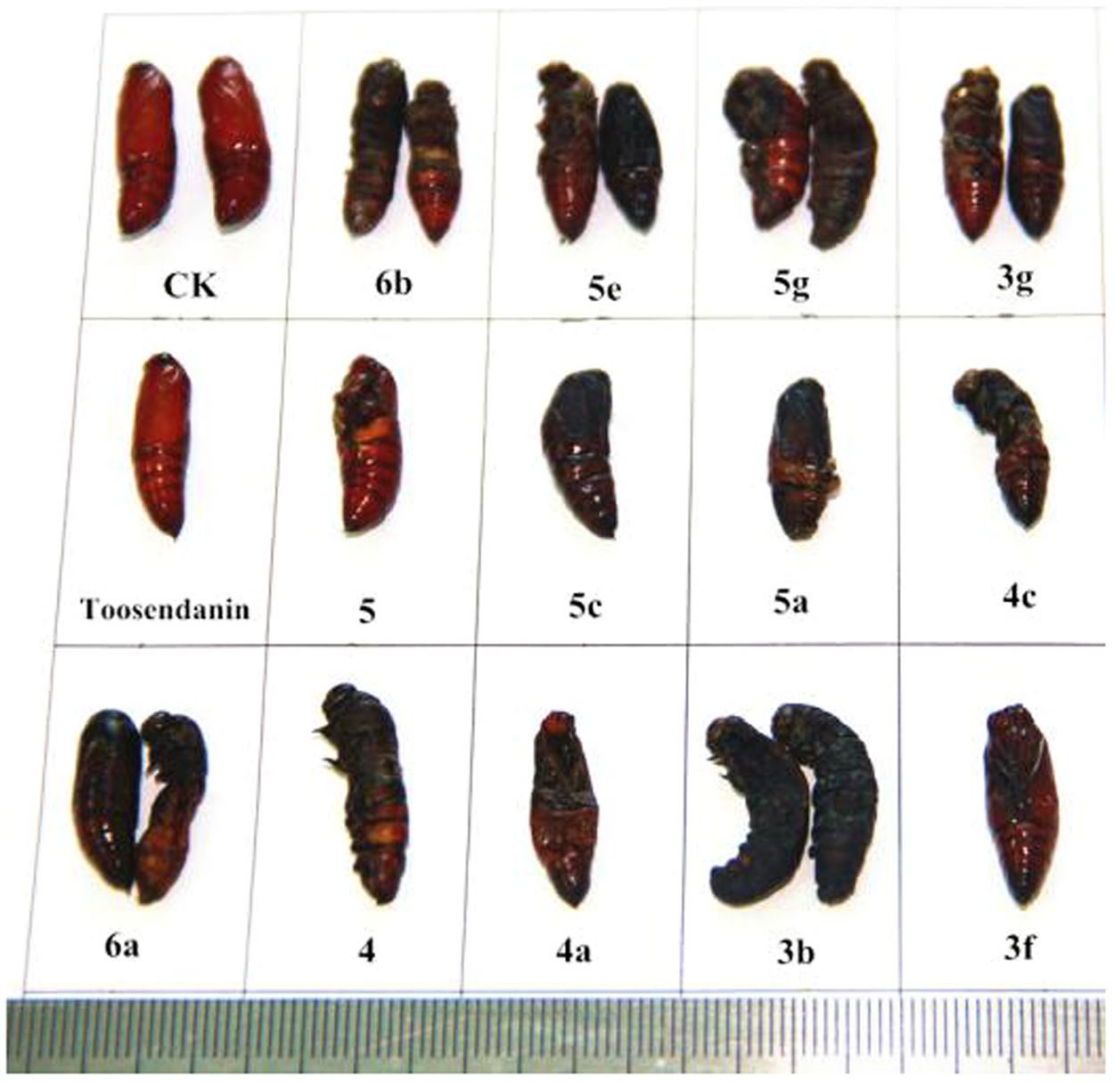
Representative malformed pupae pictures of derivatives **3b, 3f, 3g, 4, 4a, 4c, 5, 5a, 5c, 5e, 5g, 6a** and **6b** during the pupation period.

**Figure 11:**
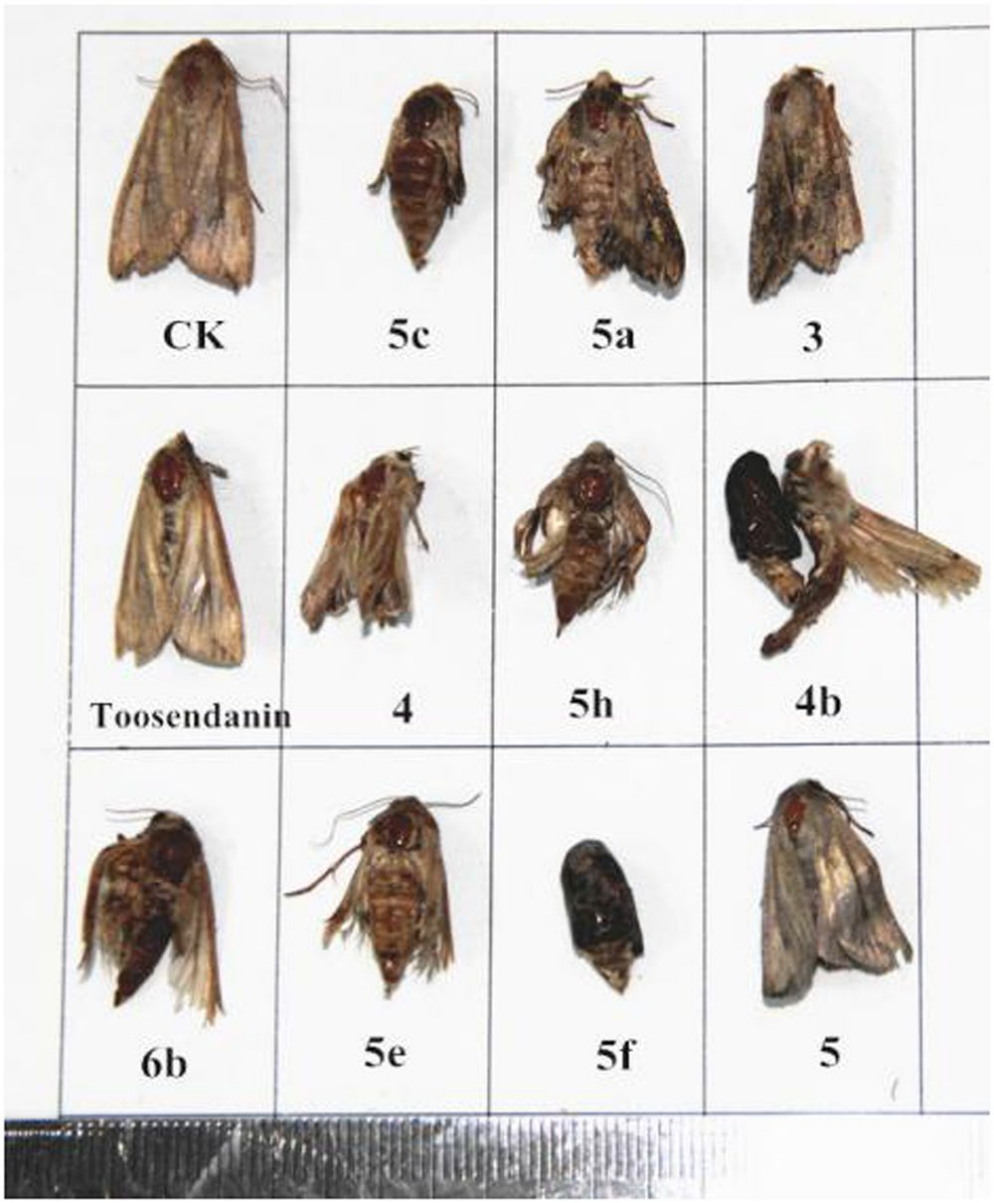
Representative malformed moth pictures of **3, 4, 4b, 5, 5a, 5c, 5h, 5e, 5f, 6b** during the stage of adult emergence (CK = blank control group, Toosendanin = positive control group).

The insecticidal activity of all derivatives against the pre-third-instar larvae of *M. separata* was tested by the leaf-dipping method as the mortality rates at 1 mg/mL^45^. Toosendanin, a commercial insecticide, was used as a positive control at 1 mg/mL, and leaves treated with acetone alone were used as a blank control group. The corrected mortality rate was outlined in Table 1. As a previous report^30^, the reduced fraxinellone **2** exhibited stronger mortality activity than precursor **1**, and the mortality rates of these tested compounds against *M. separata* after 35 days were often higher than those after 10 and 20 days. The symptoms of the larvae of *M. separate* treated by these compounds were slim and wrinkled bodies during the larval period (Fig. 9). Many larvae molted to malformed pupae or died in the treated groups during the stage of pupation (Fig. 10), and some malformed moths of the treated groups appeared with imperfect wings during the emergence period (Fig. 11), implying that these derivatives might display an antimolting hormone effect^32,33^.

**Table 1:** Insecticidal activity of all derivatives against *M. separata* Walkers on leaves treated with a concentration of 1 mg/mL.

compounds	corrected mortality rate (%)^a^		
	10 days	20 days	35 days
**1**	0.0 ± 0.0	11.3 ± 2.5	17.4 ± 4.9
**2**	53.3 ± 3.3	66.7 ± 3.2	71.4 ± 1.4
**3**	8.3 ± 0.0	8.3 ± 0.0	29.2 ± 4.2
**4**	8.3 ± 0.0	8.3 ± 0.0	20.8 ± 4.2
**5**	18.3 ± 3.3	18.3 ± 3.3	37.7 ± 4.2
**6**	20.0 ± 9.4	20.0 ± 9.4	31.1 ± 8.9
**7**	4.2 ± 3.2	4.2 ± 3.2	9.1 ± 1.7
**3a**	0.0 ± 0.0	4.2 ± 3.2	17.5 ± 1.7
**3b**	20.8 ± 0.0	25.0 ± 3.3	40.8 ± 0.8
**3c**	4.2 ± 3.2	9.2 ± 1.7	31.8 ± 4.5
**3d**	4.2 ± 3.2	8.3 ± 1.7	26.1 ± 1.1
**3e**	14.2 ± 1.6	19.2 ± 1.4	37.7 ± 4.2
**3f**	0.0 ± 0.0	0.0 ± 0.0	12.5 ± 5.7
**3g**	14.6 ± 3.9	22.9 ± 3.9	45.0 ± 3.3
**4a**	12.5 ± 0.0	12.5 ± 0.0	25.0 ± 0.0
**4b**	8.3 ± 2.0	17.5 ± 1.7	25.4 ± 3.2
**4c**	0.0 ± 0.0	0.0 ± 0.0	20.8 ± 4.2
**5a**	19.2 ± 2.0	23.3 ± 3.3	52.7 ± 4.2
**5c**	12.5 ± 0.0	16.7 ± 7.9	33.3 ± 4.2
**5d**	26.7 ± 5.0	35.0 ± 4.7	45.5 ± 4.7
**5e**	13.3 ± 1.6	17.5 ± 1.7	30.8 ± 5.7
**5f**	0.0 ± 0.0	9.2 ± 1.7	30.7 ± 5.7
**5g**	0.0 ± 0.0	4.2 ± 1.7	21.2 ± 3.4
**5h**	18.3 ± 0.0	23.3 ± 3.2	39.4 ± 6.1
**6a**	10.4 ± 4.2	10.4 ± 4.2	31.1 ± 6.1
**6b**	4.2 ± 3.2	4.2 ± 3.2	33.3 ± 0.0
toosendanin	9.2 ± 1.7	17.5 ± 3.9	39.0 ± 2.7
control	0.0 ± 0	0.0 ± 0	0.0 ± 0

^a^The corrected mortality rate was calculated in different periods. All data (mean ± SD) are the average of four independent groups (six larvae per group).

Meanwhile, some interesting SAR results of tested compounds were also observed: (1) Comparing mortality rates of two series derivatives before or after reduction of the C=C bond between the C-2 and C-3 positions, non-reduced bromofraxinellone **5**, **6** and almost all of their derivatives, except **5g**, showed a much better insecticidal activity (mortality rates >30%) than precursor **1**. The reduced derivative **2** showed the most promising insecticidal activity with a final mortality rate of 71.4%, yet its derivatives **3**, **4**, **3a-g** and **4a-c** all decreased dramatically. (2) Derivatives bearing one bromine atom on the furan ring showed stronger final mortality activity than those with two bromine atoms (**3** vs **4**, **5** vs **6**). (3) **7** showed the weakest potent of insecticidal activity, in consistence with previous report about the tetrahydrofuran fraxinellone^46^, also indicating that the aromatic property of furan ring was very important for the insecticidal activity. (4) **3b**, **3g**, **5a**, **5d** and **5h** displayed more potent insecticidal activity than toosendanin, perhaps simplying that aliphatic chain may lead to an improved level of insecticidal activity for **2**, and aromatic chain without electron withdrawing group may be beneficial for insecticidal activity of **1**.

## Conclusion

We semisynthesized a series of fraxinellone derivatives through furan-site selective bromination and Pd-catalyzed coupling reactions. The conditions of the NBS (1.4 equiv.) and 40 °C as well as DBDMH could improve the yields of bromination of fraxinellone. Notably, when the reaction was carried out with DBDMH, the dibromofraxinellone **6** was obtained in excellent yield (91%). Furthermore, a variety of alkyl, aryl and alkynyl substituents were introduced on their furan ring, and 20 derivatives were semisynthesized by Suzuki-Miyaura or Sonogashira couplings in 41–98% yields, revealing the ability of preparing new furan derivatives on furan-containing NPs. An evaluation of insecticidal activity showed that **2**, **3b**, **3g**, **5a**, **5d** and **5h** displayed more potent insecticidal activity than fraxinellone and toosendanin. However, the corresponding pharmacological data and target of action of fraxinellone and its active derivatives remained underexplored up to now. This study will provide new synthetic modification or give a highly amenable process for the synthesis of furan-containing limonoids as biological probes, which will be presented in due course.

## Methods

### Chemistry

#### General

All NMR spectra were recorded on a 500 MHz Bruker NMR spectrometer in CDCl_3_ with TMS as internal standard for ^1^H NMR and solvent signals as internal standard for ^13^C NMR. Chemical shift values are mentioned in δ (ppm) and coupling constants (*J*) are given in Hz. HR-ESI-MS spectra were recorded on an ESI-Thermo Fisher LTQ Fleet instrument spectrometer or AB Sciex 5600 Triple TOF mass spectrometer. Column chromatography (CC) was performed over silica gel (200–300 mesh, Qingdao Marine Chemical Ltd.). All reactions were monitored by thin-layer chromatography carried out on 2 cm × 5 cm pre-coated silica gel GF_254_ plates of thickness of 0.25 mm (Qingdao Marine Chemical Group, Co.) with UV light (254 nm and 365 nm), and were visualized using 5% phosphomolybdic acid followed by heating. Household compact fluorescent lamp (CFL, 28 W, Zhejiang Yankon Group Co., Ltd.) was used as light source in the reactions. All commercially available solvents and reagents were freshly purified and dried by standard techniques prior to use.

#### Reduction of **1**

NaBH_4_ (1.2 g, 31.9 mmol) was added to a solution of **1** (2 g, 8.6 mmol) in absolute EtOH (50 mL). After the reaction stirring at 40 °C for 24 hours, the EtOH was removed *in vacuo*. Then 50 mL H_2_O were added to the residue and the mixture was extracted with CHCl_3_ (50 mL × 4). The organic phase was washed with H_2_O until neutral, and then evaporated to dryness to give a light yellow oil. Flash chromatography of the yellow oil residue over silica gel (2.5 × 45 cm), using 30:1 PE-EtOAc, gave **2** (1.22 g, 60%) as a white powder.

#### Typical procedure for the synthesis of **3** and **4**

A drop of Br_2_ (171 μL, 0.17 mmol, 1 mL/mol) was added to a solution of **2** (100 mg, 0.43 mmol) and NBS (80 mg, 0.45 mmol) in dry DCM (10 mL). Stirring at room temperature was continued for 9 h and the mixture was subjected to silica gel flash chromatography directly, using 40:1 PE-EtOAc and then 20:1 PE-EtOAc, gave **3** and **4** in yields as listed in Table S1. In gram-scale, 1.3 g of compound **2** was carried out.

#### Typical procedure for the synthesis of **5**, **6** and **7**

A Schlenk tube was charged with fraxinellone (**1**) (100 mg, 0.43 mmol) and NBS (107 mg, 0.60 mmol). Then the DCM (10 mL) was added to this tube. The reaction mixture was stirred at 40 °C and kept in the dark for 3 h. After the reaction was completed, the mixture was subjected to silica gel flash chromatography directly, using 40:1 PE-EtOAc and then 20:1 PE-EtOAc, gave **5, 6** and **7** in yields as listed in Fig. 4. In gram-scale, 1.1 g of compound **1** was carried out.

#### General procedure for the synthesis of **3a-3b, 3d-3g, 5a-5b** and **5d-5h**

The typical Suzuki-Miyaura couplings were utilized with the ref.^47^. An oven-dried Schlenk tube was charged with **3** (40.4 mg, 0.129 mmol) or **5** (40.1 mg, 0.129 mmol), Pd_2_dba_3_ (4.7 mg, 0.0051 mmol), SPhos (9.8 mg, 0.020 mmol), boronic acid (0.258 mmol) and K_3_PO_4_ (82.2 mg, 0.387 mmol). The tube was evacuated and flushed with argon 5 times before adding toluene (3 mL). After stirring at room temperature for 5 min, the reaction mixture was heated to 60 °C for 16 h. Then the reaction was cooled to room temperature, diluted with EtOAc (3 mL) and filtered through a thin pad of silica gel. Solvent was evaporated under reduced pressure. Flash chromatography of the residue over silica gel (1.5 × 30 cm), using 30:1 PE-EtOAc, gave **3a-3b**, **3d-3g**, **5a-5b** and **5d-5h** in yields as listed in Figs 7 and 8.

#### General procedure for the synthesis of **4a-4b** and **6a-6b**

An oven-dried Schlenk tube was charged with **4** (50.6 mg, 0.129 mmol) or **6** (50.3 mg, 0.129 mmol), Pd_2_dba_3_ (4.7 mg, 0.005 mmol), SPhos (9.8 mg, 0.020 mmol), boronic acid (0.516 mmol) and K_3_PO_4_ (82.2 mg, 0.387 mmol). The tube was evacuated and flushed with argon 5 times before adding toluene (3 mL). After stirring at room temperature for 5 min, the reaction mixture was heated to 60 °C for 16 h. Then the reaction was cooled to room temperature, diluted with EtOAc (3 mL) and filtered through a thin pad of silica gel. Solvent was evaporated under reduced pressure. Flash chromatography of the residue over silica gel (1.5 × 30 cm), using 40:1 PE-EtOAc, gave **4a-4b** and **6a-6b** in yields as listed in Figs 7 and 8.

#### General procedure for the synthesis of **3c, 4c** and **5c**

The typical Sonogashira couplings were utilized with the ref.^48^. An oven-dried Schlenk tube was charged with **3** (40.4 mg, 0.129 mmol), **4** (50.6 mg, 0.129 mmol) or **5** (40.1 mg, 0.129 mmol), PdCl_2_(PPh_3_)_2_ (4.5 mg, 0.006 mmol) and CuI (2.5 mg, 0.013 mmol). The tube was evacuated and flushed with argon 5 times before adding toluene (3 mL) and THF-Et_3_N (1:1, 2 mL). After stirring at room temperature for 5 min, phenylethyne (258 mmol or 516 mmol) was added. Then the reaction mixture was heated to 80 °C for 16 h. When the reaction was completed, the reaction mixture was cooled to room temperature and filtered through a thin pad of silica gel, rinsing with EtOAc (70 mL). Then the filtrate was rinsed with 1 N HCl (~20 mL) and brine (~20 mL). The organic layer was dried over NaSO_4_, concentrated in vacuo. Flash chromatography of the residue over silica gel (1.5 × 30 cm), using 30:1 PE-EtOAc, gave **3c**, **4c** and **5c** in yields as listed in Figs 7 and 8.

#### Analytical characterization data

(*3R,3aR,7S,7aS*)*-3-*(*furan-3-yl*)*-3a,7-dimethylhexahydroisobenzofuran-1*(*3H*)*-one* (**2**)^30^. White powder; yield: 60%; m.p. 86–87 °C; ${[{\rm{\alpha }}]}_{{\rm{D}}}^{30}$ = −8° (*c* 0.028, in CHCl_3_); ^1^H NMR (500 MHz, CDCl_3_) δ 7.42 (t, *J* = 1.6 Hz, 1H, H-2′), 7.36 (d, *J* = 0.8 Hz, 1H, H-5′), 6.28 (d, *J* = 0.8 Hz, 1H, H-4′), 4.88 (s, 1H, H-8), 2.38(d, *J* = 4.4 Hz, 1H, H-2), 1.57–1.78 (m, 5H, H-3, 4, 5, 6), 1.41–1.51 (m,1H, H-5), 1.30 (d, *J* = 7.0 Hz, 3H, H-10), 1.15–1.25 (m, 1H, H-4),0.95(s, 3H, H-11); ^13^C NMR (125 MHz, CDCl_3_) δ 176.7, 143.8, 140.1, 121.9, 109.2, 81.9, 47.8, 43.0, 34.8, 30.8, 28.6, 22.2, 19.5, 18.5.

(*3R,3aR,7S,7aS*)*-3-*(*2-bromofuran-3-yl*)*-3a,7-dimethylhexahydroisobenzofuran-1*(*3H*)*-one* (**3**). White powder; m.p. 130–132 °C; ${[{\rm{\alpha }}]}_{{\rm{D}}}^{30}$ = −6° (*c*0.021, in CHCl_3_); ^1^H NMR (500 MHz, CDCl_3_) δ 7.45 (d, *J* = 2.1 Hz, 1H, H-5′), 6.30 (d, *J* = 2.1 Hz, 1H,H-4′), 4.83 (s, 1H, H-8), 2.40 (d, *J* = 4.6 Hz, 1H, H-2), 1.77 (ddd, *J* = 15.6, 7.8, 4.2 Hz, 1H, H-3), 1.71–1.54 (m, 4H, H-4, 5, 6), 1.49–1.42 (m, 1H, H-5), 1.32 (d, *J* = 7.1 Hz, 3H, H-10), 1.20 (dd, *J* = 13.0, 3.5 Hz, 1H, H-4), 0.93 (s, 3H, H-11); ^13^C NMR (125 MHz, CDCl_3_) δ 176.4, 144.8, 121.8, 120.4, 111.0, 81.4, 48.6, 43.9, 35.0, 30.6, 28.7, 22.2, 19.1, 18.6; HRMS (ESI): *m/z* calcd for C_14_H_18_BrO_3_ [M + H]^+^ 313.0434, found 313.0434.

(*3R,3aR,7S,7aS*)*-3-*(*2,5-dibromofuran-3-yl*)*-3a,7-dimethylhexahydroisobenzofuran-1*(*3H*)*-one* (**4**). Yellow solid; m.p. 99–101 °C; ${[{\rm{\alpha }}]}_{{\rm{D}}}^{30}$ = −6° (*c* 0.028, in CHCl_3_); ^1^H NMR (500 MHz, CDCl_3_) δ 6.27 (s, 1H, H-4′), 4.77 (s, 1H, H-8), 2.36 (d, *J* = 4.4 Hz, 1H, H-2), 1.78 (ddd, *J* = 15.8, 7.8, 4.1 Hz, 1H, H-3), 1.70–1.58 (m, 4H, H-4, 5, 6), 1.49–1.41 (m, 1H, H-5), 1.32 (d, *J* = 7.1 Hz, 3H, H-10), 1.19 (dd, *J* = 12.9, 3.5 Hz, 1H, H-4), 0.97 (s, 3H, H-11); ^13^C NMR (125 MHz, CDCl_3_) δ 176.2, 123.7, 123.46, 121.4, 112.8, 81.0, 48.7, 42.6, 35.0, 30.7, 28.7, 22.2, 19.2, 18.5; HRMS (ESI): *m/z* calcd for C_14_H_17_Br_2_O_3_ [M + H]^+^ 390.9539, found 390.9540.

(*3R,3aR*)*-3-*(*2-bromofuran-3-yl*)*-3a,7-dimethyl-3a,4,5,6-tetrahydroisobenzofuran-1*(*3H*)*-one* (**5**). Yellow solid; m.p. 128–130 °C; ${[{\rm{\alpha }}]}_{{\rm{D}}}^{30}$ = 3° (*c* 0.022, in CHCl_3_); ^1^H NMR (500 MHz, CDCl_3_) δ 7.43 (d, *J* = 1.9 Hz, 1H, H-5′), 6.50 (d, *J* = 2.0 Hz, 1H, H-4′), 4.83 (s, 1H, H-10), 2.24 (dd, *J* = 19.6, 6.8 Hz, 1H, H-4), 2.18–2.13 (overlap, 1H, H-4), 2.11 (s, 3H, H-11), 1.83–1.69 (m, 3H, H-5, 6), 1.48 (td, *J* = 13.3, 3.4 Hz, 1H, H-6), 0.91 (s, 3H, H-11); ^13^C NMR (125 MHz, CDCl_3_) δ 169.9, 149.2, 144.5, 127.1, 121.0, 120.0, 112.60, 82.6, 44.5, 32.3, 32.2, 20.8, 18.7, 18.4; HRMS (ESI): *m/z* calcd for C_14_H_16_BrO_3_ [M + H]^+^ 311.0277, found 311.0275.

(*3R,3aR*)*-3-*(*2,5-dibromofuran-3-yl*)*-3a,7-dimethyl-3a,4,5,6-tetrahydroisobenzofuran-1*(*3H*)*-one* (**6**). Yellow solid; m.p. 98–100 °C; ${[{\rm{\alpha }}]}_{{\rm{D}}}^{30}$ = −1° (*c* 0.034, in CHCl_3_); ^1^H NMR (500 MHz, CDCl_3_) δ 6.44 (s, 1H, H-4′), 4.76 (s, 1H, H-8), 2.25 (dd, *J* = 19.9, 6.6 Hz, 1H, H-4), 2.13–2.17 (overlap, 1H, H-4), 2.10 (s, 3H, H-10), 1.84–1.69 (m, 3H, H-5, 6), 1.46 (td, *J* = 13.1, 3.5 Hz, 1H, H-6), 0.92 (s, 3H, H-11); ^13^C NMR (125 MHz, CDCl_3_) δ 169.5, 149.7, 126.7, 123.2, 122.8, 120.6, 114.2, 82.1, 44.4, 32.3, 32.1, 20.89, 18.7, 18.3; HRMS (ESI): *m/z* calcd for C_14_H_15_Br_2_O_3_ [M + H]^+^ 388.9383, found 388.9383.

(*3R,3aR*)*-3-*(*5-bromo-2-oxo-2,5-dihydrofuran-3-yl*)*-3a,7-dimethyl-3a,4,5,6-tetrahydroisobenzofuran-1*(*3H*)*-one* (**7**). Light yellow solid; m.p. 143–144 °C; ${[{\rm{\alpha }}]}_{{\rm{D}}}^{30}$ = −7° (*c* 0.022, in CHCl_3_); ^1^H NMR (500 MHz, CDCl_3_): δ 7.60 (s, 1H, H-4′), 6.95 (s, 0.32H, H-5′), 6.94–6.91 (m, 0.67H, H-5′), 4.73 (s, 0.31H, H-8), 4.71 (t, *J* = 1.8 Hz, 0.68H, H-8), 2.25 (dd, *J* = 19.8, 6.6 Hz, 1H, H-4), 2.19–2.10 (m, 1H, H-4), 2.09 (s, 3H, H-11), 2.05 (dt, *J* = 12.6, 3.5 Hz, 1H, H-5), 1.87–1.76 (m, 1H, H-6), 1.70–1.64 (m, 1H, H-5), 1.61–1.44 (m, 1H, H-6), 0.92 (s, 2.16H, H-10), 0.86 (s, 1.14H, H-10). ^13^C NMR (125 MHz, CDCl_3_): δ 168.7, 168.6, 168.2, 168.1, 151.2, 151.2, 150.3, 150.1, 131.0, 130.8, 126.2, 126.1, 81.9, 81.4, 74.9, 74.8, 43.5, 42.9, 32.2, 32.2, 32.2, 32.1, 21.0, 20.9, 18.8, 18.3, 18.3.

(*3R,3aR*)*-6-bromo-3-*(*2-bromofuran-3-yl*)*-3a,7-dimethyl-3a,4,5,6-tetrahydroisobenzofuran-1*(*3H*)*-one* (**8**). White powder; m.p. 102–103 °C; ${[{\rm{\alpha }}]}_{{\rm{D}}}^{30}$ = −27° (*c* 0.018, in CHCl_3_); ^1^H NMR (500 MHz, CDCl_3_): δ 7.46 (d, *J* = 2.0 Hz, 1H, H-5′), 6.50 (d, *J* = 2.0 Hz, 1H, H-4′), 4.96 (s, 1H, H-8), 4.71 (d, *J* = 3.0 Hz, 1H, H-4), 2.33–2.28 (m, 1H, H-5), 2.27 (s, 3H, H-11), 2.24–2.19 (m, 1H, H-5), 2.04–2.09 (m, 1H, H-6), 1.69 (dt, *J* = 13.0, 3.2 Hz, 1H, H-6), 0.93 (s, 3H, H-10); ^13^C NMR (125 MHz, CDCl_3_): δ 169.2, 145.3, 144.7, 129.8, 121.3, 119.3, 112.5, 82.1, 51.9, 44.9, 29.3, 27.3, 20.9, 17.6.

(*3R,3aR,7S,7aS*)*-3a,7-dimethyl-3-*(*2-phenylfuran-3-yl*)*hexahydroisobenzofuran-1*(*3H*)*-one* (**3a**). Yellowish oil; yield: 95%; ${[{\rm{\alpha }}]}_{{\rm{D}}}^{30}$ = −3° (*c* 0.020, in CHCl_3_); ^1^H NMR (500 MHz, CDCl_3_) δ 7.5–7.50 (m, 2H, Ph-H), 7.45 (d, *J* = 1.8 Hz, 1H, H-5′), 7.34–7.44 (m, 3H, Ph-H), 6.37 (d, *J* = 1.9 Hz, 1H, H-4′), 5.27 (s, 1H, H-8), 2.52 (d, *J* = 4.5 Hz, 1H, H-2), 1.83–1.75 (m, 1H, H-3), 1.68–1.57 (m, 4H, H-4, 5, 6), 1.49–1.39 (m, 1H, H-5), 1.34 (d, *J* = 7.1 Hz, 3H, H-10), 1.22 (dd, *J* = 12.9, 2.6 Hz, 1H, H-4), 0.96 (s, 3H, H-11); ^13^C NMR (125 MHz, CDCl_3_) δ 176.6, 151.4, 142.3, 130.6, 129.0 (2C), 128.7, 126.8 (2C), 117.6, 110.7, 81.6, 48.9, 43.8, 35.6, 30.9, 28.8, 22.4, 19.4, 18.7; HRMS (ESI): *m/z* calcd for C_20_H_23_O_3_ [M + H]^+^ 311.1642, found 311.1642.

(*3R,3aR,7S,7aS*)*-3-*(*2-butylfuran-3-yl*)*-3a,7-dimethylhexahydroisobenzofuran-1*(*3H*)*-one* (**3b**). Colorless oil; yield: 56%; ${[{\rm{\alpha }}]}_{{\rm{D}}}^{30}$ = −11° (*c* 0.026, in CHCl_3_); ^1^H NMR (500 MHz, CDCl_3_) δ 7.26 (d, *J* = 1.9 Hz, 1H), 6.15 (d, *J* = 1.9 Hz, 1H), 4.81 (s, 1H), 2.56 (t, *J* = 7.6 Hz, 2H, –CH_2_CH_2_CH_2_CH_3_), 2.45 (d, *J* = 4.6 Hz, 1H, H-2), 1.80–1.72 (m, 1H, H-3), 1.68–1.56 (m, 6H, –CH_2_CH_2_CH_2_CH_3_ and H-4, 5, 6), 1.45–1.40 (m, 1H, H-5), 1.33–1.28 (m, 5H, –CH_2_CH_2_CH_2_CH_3_ and H-10), 1.20 (dd, *J* = 12.9, 3.5 Hz, 1H, H-6), 0.90 (t, *J* = 7.4 Hz, 3H, –CH_2_CH_2_CH_2_CH_3_), 0.87 (s, 3H, H-11); ^13^CNMR (125 MHz, CDCl_3_) δ 176.8, 154.0, 141.2, 115.4, 109.1, 82.1, 48.8, 43.6, 35.5, 30.9, 30.8, 28.9, 26.3, 22.5, 22.4, 19.6, 18.7, 14.0; HRMS (ESI): *m/z* calcd for C_18_H_27_O_3_ [M + H]^+^ 291.1955, found 291.1955.

(*3R,3aR,7S,7aS*)*-3a,7-dimethyl-3-*(*2-*(*phenylethynyl*)*furan-3-yl*)*hexahydroisobenzofuran-1*(*3H*)*-one* (**3c**). Red oil; yield: 82%; ${[{\rm{\alpha }}]}_{{\rm{D}}}^{30}$ = −2° (*c* 0.021, in CHCl_3_); ^1^H NMR (500 MHz, CDCl_3_) δ 7.52–7.48 (m, 2H, Ph-H), 7.38 (d, *J* = 1.8 Hz, 1H, H-5′), 7.35–7.33 (m, 3H, Ph-H), 6.31 (d, *J* = 1.8 Hz, 1H, H-4′), 5.02 (s, 1H, H-8), 2.48 (d, *J* = 4.5 Hz, 1H, H-2), 1.77–1.73 (m, 1H, H-3), 1.69–1.61 (m, 4H, H-4, 5, 6), 1.43–1.38 (m, 1H, H-5), 1.30 (d, *J* = 7.1 Hz, 3H, H-10), 1.20 (dd, *J* = 13.2, 3.0 Hz, 1H, H-4), 0.95 (s, 3H, H-11); ^13^CNMR (125 MHz, CDCl_3_) δ 176.7, 144.0, 140.9, 131.6 (2C), 129.2, 128.7 (2C), 126.6, 122.1, 110.6, 97.2, 81.9, 78.3, 48.7, 44.4, 35.2, 30.9, 28.7, 22.4, 19.5, 18.6; HRMS (ESI): *m/z* calcd for C_22_H_23_O_3_ [M + H]^+^ 335.1642, found 335.1642.

(*3R,3aR,7S,7aS*)*-3a,7-dimethyl-3-*(*2-*(*naphthalen-2-yl*)*furan-3-yl*)*hexahydroisobenzofuran-1*(*3H*)*-one* (**3d**). White powder; yield: 85%; m.p. 144–146 °C; ${[{\rm{\alpha }}]}_{{\rm{D}}}^{30}$ = −9° (*c* 0.014, in CHCl_3_); ^1^H NMR (500 MHz, CDCl_3_) δ 7.95 (s, 1H, Nap-H), 7.89 (d, *J* = 8.6 Hz, 1H, Nap-H), 7.84 (ddd, *J* = 8.5, 5.3, 2.9 Hz, 2H, Nap-H), 7.67 (dd, *J* = 8.6, 1.7 Hz, 1H, Nap-H), 7.53–7.47 (m, 3H, Nap-H and H-5′), 6.42 (d, *J* = 1.9 Hz, 1H, H-4′), 5.40 (s, 1H, H-8), 2.54 (d, *J* = 4.6 Hz, 1H, H-2), 1.85–1.74 (m, 1H, H-3), 1.69–1.61 (m, 4H, H-4, 5, 6), 1.51–1.39 (m, 1H, H-5), 1.34 (d, *J* = 7.1 Hz, 3H, H-10), 1.22 (dd, *J* = 12.9, 3.1 Hz, 1H, H-4), 0.99 (s, 3H, H-11); ^13^CNMR (125 MHz, CDCl_3_) δ 176.6, 151.5, 142.5, 133.4, 133.0, 128.8, 128.5, 128.0, 127.9, 126.9, 126.8, 125.9, 124.4, 118.1, 110.9, 81.8, 48.9, 43.9, 35.7, 30.9, 28.9, 22.4, 19.5, 18.7; HRMS (ESI): *m/z* calcd for C_24_H_25_O_3_ [M + H]^+^ 361.1798, found 361.1798.

*Methyl 2-*(*3-*((*1R,3aS,4S,7aR*)*-4,7a-dimethyl-3-oxooctahydroisobenzofuran-1yl*)*-furan-2-yl*) *benzoate* (**3e**). Yellow solid; yield: 57%; m.p. 121–123 °C; ${[{\rm{\alpha }}]}_{{\rm{D}}}^{30}$ = −5° (*c* 0.032, in CHCl_3_); ^1^H NMR (500 MHz, CDCl_3_) δ 7.92 (dd, *J* = 7.7, 1.2 Hz, 1H, Ph-H), 7.55 (td, *J* = 7.5, 1.4 Hz, 1H, Ph-H), 7.48 (td, *J* = 7.6, 1.3 Hz, 1H, Ph-H), 7.45–7.42 (m, 2H, Ph-H and H-5′), 6.38 (d, *J* = 1.9 Hz, 1H, H-4′), 4.89 (s, 1H, H-8), 3.73 (s, 3H, -OMe), 2.44 (d, *J* = 4.5 Hz, 1H, H-2), 1.76–1.70 (m, 1H, H-3), 1.5–1.41 (m, 4H, H-4, 5, 6), 1.38–1.34 (m, 1H, H-5), 1.25 (d, *J* = 7.1 Hz, 3H, H-10), 1.14 (dd, *J* = 12.7, 3.1 Hz, 1H, H-4), 0.90 (s, 3H, H-11); ^13^CNMR (125 MHz, CDCl3) δ 176.7, 167.7, 151.2, 142.5, 132.0, 131.9, 131.4, 130.8, 130.3, 129.4, 117.8, 110.4, 82.0, 52.6, 48.9, 43.9, 35.3, 30.8, 28.8, 22.2, 19.3, 18.7; HRMS (ESI): *m/z* calcd for C_22_H_25_O_5_ [M + H]^+^ 369.1697, found 369.1697.

(*3R,3aR,7S,7aS*)*-3a,7-dimethyl-3-*(*2-*(*2-*(*trifluoromethyl*)*phenyl*)*furan-3-yl*)*hexahydroisobenzofuran-1*(*3H*)*-one* (**3f**). White powder; yield 54%; m.p. 95–96 °C; ${[{\rm{\alpha }}]}_{{\rm{D}}}^{30}$ = −23° (*c* 0.015, in CHCl_3_); ^1^H NMR (500 MHz, CDCl_3_) δ 7.78 (d, *J* = 7.4 Hz, 1H, Ph-H), 7.62–7.53 (m, 2H, Ph-H), 7.49 (d, *J* = 2.0 Hz, 1H, H-5′), 7.46 (d, *J* = 7.5 Hz, 1H, Ph-H), 6.41 (d, *J* = 2.0 Hz, 1H, H-4′), 4.77 (s, 1H, H-8), 2.48 (d, *J* = 4.6 Hz, 1H, H-2), 1.81–1.69 (m, 1H, H-3), 1.61–1.47 (m, 2H, H-5, 6), 1.45–1.32 (m, 3H, H-4, 5, 6), 1.28 (d, *J* = 7.1 Hz, 3H, H-10), 1.14 (dd, *J* = 12.7, 3.1Hz, 1H, H-4), 0.88 (s, 3H, H-11); ^13^CNMR (125 MHz, CDCl_3_) δ 176.5, 149.6, 143.1, 132.9, 132.0, 130.4, 129.9, 128.3, 127.3, 123.8, 119.1, 110.1, 81.7, 49.0, 43.6, 35.4, 30.8, 28.9, 22.2, 19.3, 18.7; HRMS (ESI): *m/z* calcd for C_21_H_22_F_3_O_3_ [M + H]^+^ 379.1516, found 379.1515.

(*3R,3aR,7S,7aS*)*-3-*(*2-cyclopropylfuran-3-yl*)*-3a,7-dimethylhexahydroisobenzofuran-1*(*3H*)*-one* (**3g**). White powder; yield: 58%; m.p. 78–79 °C; ${[{\rm{\alpha }}]}_{{\rm{D}}}^{30}$ = −8° (*c* 0.018, in CHCl_3_); ^1^H NMR (500 MHz, CDCl_3_) δ 7.14 (d, *J* = 2.0 Hz, 1H,H-5′), 6.14 (d, *J* = 2.0 Hz, 1H, H-4′), 4.99 (s, 1H, H-8), 2.44 (d, *J* = 4.6 Hz, 1H, H-2), 1.81–1.72 (m, 2H, cyclopropane-H and H-3), 1.61–1.51 (m, 4H, H-4, 5, 6), 1.48–1.38 (m, 1H, H-5), 1.31 (d, *J* = 7.1 Hz, 3H, H-10), 1.20 (dd, *J* = 13.0, 3.4 Hz, 1H, H-4), 0.91–0.86 (m, 7H, Cyclopropane-H and H-11); ^13^CNMR (125 MHz, CDCl_3_) δ 177.0, 153.3, 140.1, 115.8, 109.7, 82.0, 48.8, 43.8, 35.3, 30.9, 28.8, 22.4, 19.5, 18.7, 7.9, 6.8, 6.7; HRMS (ESI): *m/z* calcd for C_17_H_23_O_3_ [M + H]^+^ 275.1642, found 275.1645.

(*3R,3aR,7S,7aS*)*-3-*(*2,5-diphenylfuran-3-yl*)*-3a,7-dimethylhexahydroisobenzofuran-1*(*3H*)*-one* (**4a**). White powder; yield: 98%; m.p. 171–173 °C; ${[{\rm{\alpha }}]}_{{\rm{D}}}^{30}$ = −2° (*c* 0.011, in CHCl_3_); ^1^H NMR (500 MHz, CDCl_3_) δ 7.72 (d, *J* = 8.1 Hz, 2H, Ph-H), 7.61 (d, *J* = 8.1 Hz, 2H, Ph-H), 7.35–7.47 (m, 4H, Ph-H), 7.31–7.23 (m, 2H, Ph-H), 6.62 (s, 1H, H-4′), 5.33 (s, 1H, H-8), 2.58 (d, *J* = 4.0 Hz, 1H, H-2′), 1.861.76 (m, 1H, H-3), 1.73–1.58 (m, 4H, H-4, 5, 6), 1.51–1.43 (m, 1H, H-5), 1.38 (d, *J* = 7.0 Hz, 3H, H-10), 1.25–1.18 (m, 1H, H-4), 1.02 (s, 3H, H-11); ^13^CNMR (125 MHz, CDCl_3_) δ 176.6, 153.5, 150.5, 130.7, 130.2, 129.1 (2C), 129.0 (2C), 128.3, 128.1, 126.6 (2C), 124.2 (2C), 119.9, 105.9, 81.7, 48.9, 43.9, 35.7, 30.9, 28.9, 22.4, 19.5, 18.7; HRMS (ESI): *m/z* calcd for C_26_H_27_O_3_ (M + H)^+^ 387.1955, found 387.1956.

(*3R,3aR,7S,7aS*)*-3-*(*2,5-dibutylfuran-3-yl*)*-3a,7-dimethylhexahydroisobenzofuran-1*(*3H*)*-one* (**4b**). Colorless oil; yield: 57%; ${[{\rm{\alpha }}]}_{{\rm{D}}}^{30}$ = −8° (*c* 0.013, in CHCl_3_); ^1^H NMR (500 MHz, CDCl_3_) δ 5.72 (s, 1H, H-5′), 4.75 (s, 1H, H-8), 2.57–2.48 (m, 4H, –CH_2_CH_2_CH_2_CH_3_), 2.46 (d, *J* = 4.5 Hz, 1H, H-2), 1.79–1.72 (m, 1H, H-3), 1.60–1.52 (m, 9H, –CH_2_CH_2_CH_2_CH_3_ and H-4, 5, 6), 1.35–1.31 (m, 7H, –CH_2_CH_2_CH_2_CH_3_ and H-10), 1.17 (dd, *J* = 7.3, 2.6 Hz, 1H, H-4), 0.92–0.87 (m, 9H, –CH_2_CH_2_CH_2_CH_3_ and H-11); ^13^CNMR (125 MHz, CDCl_3_) δ 177.0, 155.2, 152.1, 115.6, 104.0, 82.4, 48.9, 43.5, 35.6, 31.0, 30.2, 29.9, 29.0, 27.9, 26.3, 22.6, 22.5 (2C), 19.6, 18.8, 14.0 (2C); HRMS (ESI): *m/z* calcd for C_22_H_35_O_3_ [M + H]^+^ 347.2581, found 347.2579.

(*3R,3aR,7S,7aS*)*-3-*(*2,5-bis*(*phenylethynyl*)*furan-3-yl*)*-3a,7-dimethylhexahydroisobenzofuran-1*(*3H*)*-one* (**4c**). Brown powder; yield: 90%; m.p. 147–149 °C; ${[{\rm{\alpha }}]}_{{\rm{D}}}^{30}$ = −5° (*c* 0.021, in CHCl_3_); ^1^H NMR (500 MHz, CDCl_3_) δ 7.5–7.48 (m, 4H, Ph-H), 7.37–7.33 (m, 6H, Ph-H), 6.55 (s, 1H, H-4′), 5.00 (s, 1H, H-8), 2.49 (d, *J* = 4.4 Hz, 1H, H-2), 1.81–1.72 (m, 1H, H-3), 1.70–1.56 (m, 4H, H-4, 5, 6), 1.50–1.42 (m, 1H, H-5), 1.31 (d, *J* = 7.1 Hz, 3H, H-10), 1.20 (dd, *J* = 12.8, 3.3 Hz, 1H, H-4), 0.99 (s, 3H, H-11); ^13^CNMR (125 MHz, CDCl_3_) δ 176.5, 137.9, 135.3, 131.7 (4C), 129.4 (2C), 128.7 (4C), 127.6, 121.8, 121.7, 115.2, 97.9, 94.9, 81.5, 78.9, 78.2, 48.6, 44.3, 35.09, 30.8, 28.7, 22.3, 19.5, 18.6; HRMS (ESI): *m/z* calcd for C_30_H_27_O_3_ [M + H]^+^ 435.1955, found 435.1955.

(*3R,3aR*)*-3a,7-dimethyl-3-*(*2-phenylfuran-3-yl*)*-3a,4,5,6-tetrahydroisobenzofuran-1*(*3H*)*-one* (**5a**). Yellowish green oil; yield: 87%; ${[{\rm{\alpha }}]}_{{\rm{D}}}^{30}$ = −2° (*c* 0.035, in CHCl_3_); ^1^H NMR (500 MHz, CDCl_3_) δ 7.55–7.51 (m, 2H, Ph-H), 7.44 (d, *J* = 1.9 Hz, 1H, H-5′), 7.40–7.29 (m, 3H, Ph-H), 6.61 (d, *J* = 1.9 Hz, 1H, H-4′), 5.28 (s, 1H, H-8), 2.21 (dd, *J* = 20.2, 6.1 Hz, 1H, H-4), 2.12–2.06 (m, 4H, H-10, 4), 1.73–1.62 (m, 2H, H-5), 1.50 (dt, *J* = 12.3, 3.4 Hz, 1H, H-6), 1.29 (td, *J* = 12.9, 4.1 Hz, 1H, H-6), 0.97 (s, 3H, H-11); ^13^CNMR (125 MHz, CDCl_3_) δ 170.0, 151.4, 148.6, 141.8, 130.9, 128.8 (2C), 128.4, 127.7, 127.2 (2C), 116.5, 112.4, 82.8, 44.7, 32.3, 32.2, 21.0, 18.6, 18.4; HRMS (ESI): *m/z* calcd for C_20_H_21_O_3_ [M + H]^+^ 309.1485, found 309.1486.

(*3R,3aR*)*-3-*(*2-butylfuran-3-yl*)*-3a,7-dimethyl-3a,4,5,6-tetrahydroisobenzofuran-1*(*3H*)*-one* (**5b**). Yellow oil; yield: 54%; ${[{\rm{\alpha }}]}_{{\rm{D}}}^{30}$ = −4° (*c* 0.051, in CHCl_3_); ^1^H NMR (500 MHz, CDCl_3_) δ 7.27 (d, *J* = 1.9 Hz, 1H, H-5′), 6.33 (d, *J* = 1.9 Hz, 1 H, H-4′), 4.84 (s, 1 H, H-8), 2.59–2.55 (m, 2 H, –CH_2_CH_2_CH_2_CH_3_), 2.25 (dd, *J* = 19.5, 6.7 Hz, 1H, H-4), 2.17–2.11 (m, 4H, –CH_2_CH_2_CH_2_CH_3_ and H-4), 1.72–1.55 (m, 5H, –CH_2_CH_2_CH_2_CH_3_ and H-5, 6), 1.39–1.30 (m, 3H, –CH_2_CH_2_CH_2_CH_3_ and H-6), 0.92–0.88 (m, 6H, –CH_2_CH_2_CH_2_CH_3_ and H-11); ^13^C NMR (125 MHz, CDCl_3_) δ 170.4, 153.6, 148.3, 140.8, 127.9, 114.1, 110.4, 83.6, 44.1, 32.3, 32.0, 30.9, 26.6, 22.6, 20.7, 18.6, 18.4, 14.0; HRMS (ESI): *m/z* calcd for C_18_H_25_O_3_ [M + H]^+^ 289.1798, found 289.1794.

(*3R,3aR*)*-3a,7-dimethyl-3-*(*2-phenylfuran-3-yl*)*-3a,4,5,6-tetrahydroisobenzofuran-1*(*3H*)*-one* (**5c**). Reddish brownoil; yield: 85%; ${[{\rm{\alpha }}]}_{{\rm{D}}}^{30}$ = +8° (*c*0.010, in CHCl_3_); ^1^H NMR (500 MHz, CDCl_3_) δ 7.49–7.45 (m, 2H, Ph-H), 7.39 (d, *J* = 1.9 Hz, 1H, H-5′), 7.36–7.33 (m, 3H, Ph-H), 6.55 (d, *J* = 1.9 Hz, 1H, H-4′), 5.10 (s, 1H, H-4, H-8), 2.25 (dd, *J* = 19.8, 6.7 Hz, 1H, H-4), 2.19–2.11 (m, 4H, H-10, 4), 1.89 (dt, *J* = 12.4, 3.3 Hz, 1H, H-5), 1.80–1.67 (m, 2H, H-5, 6), 1.51 (td, *J* = 13.2, 3.8 Hz, 1H, H-6), 0.92 (s, 3H, H-11); ^13^C NMR (125 MHz, CDCl_3_) δ 170.0, 149.0, 143.8, 134.6, 131.5 (2C), 129.2, 128.7 (2C), 127.4, 126.1, 122.2, 111.3, 96.8, 83.0, 78.4, 44.7, 32.3, 32.1, 20.8, 18.7, 18.5; HRMS (ESI): *m/z* calcd for C_22_H_21_O_3_ [M + H]^+^ 333.1485, found 333.1486.

(*3R,3aR*)*-3a,7-dimethyl-3-*(*2-*(*naphthalen-2-yl*)*furan-3-yl*)*-3a,4,5,6-tetrahydroisobenzofuran-1*(*3H*)*-one* (**5d**). Yellow power; yield: 98%; m.p. 57–58 °C; ${[{\rm{\alpha }}]}_{{\rm{D}}}^{30}$ = +27° (*c* 0.025, in CHCl_3_); ^1^H NMR (500 MHz, CDCl_3_) δ 8.01 (s, 1H, Nap-H), 7.87–7.80 (m, 3H,Nap-H), 7.68 (dd, *J* = 8.5, 1.7 Hz, 1H, Nap-H), 7.53–7.46 (m, 3H, Nap-H and H-5′), 6.67 (d, *J* = 1.9 Hz, 1H, H-4′), 5.42 (s, 1H, H-8), 2.21 (dd, *J* = 20.1, 6.5 Hz, 1H, H-4), 2.11 (s, 3H, H-10), 2.09–2.02 (overlap, 1H, H-4), 1.70–1.58 (m, 2H, H-5), 1.55–1.50 (m, 1H, H-6), 1.32 (td, *J* = 12.7, 4.4 Hz, 1H, H-6), 0.99 (s, 3H, H-11); ^13^C NMR (125 MHz, CDCl_3_) δ 170.1, 151.3, 148.8, 142.1, 133.3, 133.0, 128.6, 128.4, 128.3, 127.9, 127.6, 126.8, 126.7, 126.3, 124.8, 117.0, 112.6, 82.9, 44.8, 32.4, 32.2, 21.1, 18.7, 18.4. HRMS (ESI): *m/z* calcd for C_24_H_23_O_3_ [M + H]^+^ 359.1642, found 359.1642.

*Methy2-*(*3-*((*1R,7aR*)*-4,7a-dimethyl-3-oxo-1,3,5,6,7,7-hexahydroisobenzofuran-1-yl*)*furan-2-yl*)*benzoate* (**5e**). Yellow oil; yield: 70%; ${[{\rm{\alpha }}]}_{{\rm{D}}}^{30}$ = +6° (*c* 0.023, in CHCl_3_); ^1^H NMR (500 MHz, CDCl_3_) δ 7.83 (d, *J* = 7.7 Hz, 1H, Ph-H), 7.46–7.42 (m, 1H,Ph-H), 7.34–7.39 (m, 3H,Ph-H and H-5′), 6.56–6.47 (d, *J* = 1.9 Hz, 1H, H-4′), 4.90 (s, 1H, H-8), 3.64 (s, 3H, H-OMe), 2.09–2.01 (m, 1H, H-4), 1.96 (s, 3H, H-10), 1.95–1.81 (m, 2H, H-4,5), 1.50–1.44 (m, 2H, H-5,6), 1.20–1.16 (m, 1H, H-6), 0.85 (s, 3H, H-11); ^13^C NMR (125 MHz, CDCl_3_) δ 170.3, 167.7, 150.0, 148.5, 142.1, 131.9, 131.8, 130.9, 130.8 (2C), 129.3, 127.6, 117.1, 111.5, 83.2, 52.6, 44.5, 32.2, 31.3, 20.6, 18.6, 18.3; HRMS (ESI): *m/z* calcd for C_22_H_23_O_5_ [M + H]^+^ 367.1540, found 367.1541.

(*3R,3aR*)*-3a,7-dimethyl-3-*(*2-*(*2-*(*trifluoromethyl*)*phenyl*)*furan-3-yl*)*-3a,4,5,6-tetrahydroisobenzofuran-1*(*3H*)*-one* (**5f**). Yellow oil; yield: 72%; ${[{\rm{\alpha }}]}_{{\rm{D}}}^{30}$ = −7° (*c* 0.031, in CHCl_3_); ^1^H NMR (500 MHz, CDCl_3_) δ 7.76 (d, *J* = 7.4 Hz, 1H, Ph - H), 7.6–7.51 (m, 2H, Ph-H), 7.49 (d, *J* = 1.9 Hz, 1H, H-5′), 7.46 (d, *J* = 7.3 Hz, 1H, Ph-H), 6.62 (d, *J* = 1.9 Hz, 1H, H-4′), 4.87 (s, 1H, H-8), 2.18–2.11 (m, 1H, H-4), 2.05 (d, *J* = 7.2 Hz, 3H, H −10), 2.01–1.90 (m, 1H, H-4), 1.6–1.19 (m, 4H, H-5, 6), 0.92 (s, 3H, H-11); ^13^C NMR (125 MHz, CDCl_3_) δ 170.15, 148.7, 148.2, 142.7, 132.35, 131.9, 130.4, 129.7, 128.8, 127.5, 127.2,123.8, 118.4, 111.4, 82.9, 44.3, 32.1, 31.25, 20.7, 18.6, 18.3; HRMS (ESI): *m/z* calcd for C_21_H_20_F_3_O_3_ [M + H]^+^ 377.1359, found 377.1359.

(*3R,3aR*)*-3-*(*2-cyclopropylfuran-3-yl*)*-3a,7-dimethyl-3a,4,5,6-tetrahydroisobenzofuran-1*(*3H*)*-one* (**5g**). White powder; yield: 52%; m.p. 114–116 °C; ${[{\rm{\alpha }}]}_{{\rm{D}}}^{30}$ = −2° (*c* 0.073, in CHCl_3_); ^1^H NMR (500 MHz, CDCl_3_) δ 7.14 (d, *J* = 1.9 Hz, 1H, H-5′), 6.32 (d, *J* = 1.9 Hz, 1H, H-4′), 4.99 (s, 1H, H-8), 2.25 (dd, *J* = 20.1, 6.5 Hz, 1H, H-4), 2.17–2.08 (m, 4H, H-10, 4), 1.84–1.71 (m, 4H, Cyclopropane-H and H-5, 6), 1.45–1.37 (m, 1H, H-6), 0.92–0.83 (m, 7H, Cyclopropane-H and H-11); ^13^C NMR (125 MHz, CDCl_3_) δ 170.5, 152.9, 148.3, 139.7, 127.9, 114.6, 110.8, 83.6, 44.3, 32.3, 32.0, 20.7, 18.7, 18.5, 8.1, 6.9, 6.7; HRMS (ESI): *m/z* calcd for C_17_H_21_O_3_ [M + H]^+^ 273.1485, found 273.1489.

(*3R,3aR*)*-3-*(*2-*(*3-*(*hydroxymethyl*)*phenyl*)*furan-3-yl*)*-3a,7-dimethyl-3a,4,5,6-tetrahydroisobenzofuran-1*(*3H*)*-one* (**5h**). Brown oil; yield: 41%; ${[{\rm{\alpha }}]}_{{\rm{D}}}^{30}$ = +7° (*c* 0.010, in CHCl_3_); ^1^H NMR (500 MHz, CDCl_3_) δ 7.58 (s, 1H, Ph-H), 7.49–7.45 (m, 2H, Ph-H and H-5′), 7.38 (t, *J* = 6.4 Hz, 1H, Ph-H), 7.31 (d, *J* = 7.6 Hz, 1H, Ph-H), 6.61 (d, *J* = 1.9 Hz, 1H, H-4′), 5.29 (s, 1H, H-8), 4.72 (s, 2H, -CH_2_OH), 2.22 (dd, *J* = 19.3, 6.9 Hz, 1H, H-4), 2.16–2.12 (m, 1H, H-4), 2.10 (s, 3H, H-10), 1.74–1.62 (m, 3H, H-5, 6), 1.30 (m, 1H, H-6), 0.97 (s, 3H, H-11); ^13^C NMR (125 MHz, CDCl_3_) δ 170.1, 151.1, 148.8, 141.9, 141.6, 131.2, 129.0, 127.6, 126.9, 126.3, 125.7, 116.7, 112.5, 82.8, 65.3, 44.7, 32.4, 32.2, 21.1, 18.7, 18.4; HRMS (ESI): *m/z* calcd for C_21_H_23_O_4_ [M + H]^+^ 339.1591, found 339.1591.

(*3R,3aR*)*-3-*(*2,5-diphenylfuran-3-yl*)*-3a,7-dimethyl-3a,4,5,6-tetrahydroisobenzofuran-1*(*3H*)*-one* (**6a**). Yellow powder; yield: 73%; m.p. 79–81 °C; ${[{\rm{\alpha }}]}_{{\rm{D}}}^{30}$ = +11° (*c* 0.021, in CHCl_3_); ^1^H NMR (500 MHz, CDCl_3_) δ 7.72 (dd, *J* = 8.2, 1.0 Hz, 2H, Ph-H), 7.65–7.61 (m, 2H, Ph-H), 7.45–7.36 (m, 4H, Ph-H), 7.35–7.24 (m, 2H, Ph-H), 6.90 (s, 1H, H-4′), 5.34 (s, 1H, H-8), 2.23 (dd, *J* = 19.5, 6.6 Hz, 1H, H-4), 2.14–2.06 (m, 4H, H-10, 4), 1.76–1.63 (m, 2H, H-5), 1.54 (dt, *J* = 12.2, 3.3 Hz, 1H, H-6), 1.35 (td, *J* = 13.0, 4.0 Hz, 1H, H-6), 1.03 (s, 3H, H-11); ^13^C NMR (125 MHz, CDCl_3_) δ 170.0, 153.0, 150.5, 148.8, 130.9, 130.5, 128.9 (4C), 128.4, 127.9, 127.6, 127.1 (2C), 124.1 (2C), 118.8, 107.8, 82.7, 44.7, 32.5, 32.2, 21.1, 18.7, 18.5. HRMS (ESI): *m/z* calcd for C_26_H_25_O_3_ [M + H]^+^ 385.1798, found 385.1798.

(*3R,3aR*)*-3-*(*2,5-dibutylfuran-3-yl*)*-3a,7-dimethyl-3a,4,5,6-tetrahydroisobenzofuran-1*(*3H*)*-one* (**6b**). Colorless oil; yield: 57%; ${[{\rm{\alpha }}]}_{{\rm{D}}}^{30}$ = −4° (*c* 0.032, in CHCl_3_); ^1^H NMR (500 MHz, CDCl_3_) δ 5.91 (s, 1H, H-4′), 4.78 (s, 1H, H-8), 2.59–2.47 (m, 4H, –CH_2_CH_2_CH_2_CH_3_), 2.23 (dd, *J* = 19.9, 6.2 Hz, 1H, H-4), 2.14–2.08 (m, 4H, H-4, 10), 1.65–1.53 (m, 7H, –CH_2_CH_2_CH_2_CH_3_ and H-5, 6), 1.36–1.30 (m, 5H, –CH_2_CH_2_CH_2_CH_3_ and H-6), 0.93–0.88 (m, 9H, –CH_2_CH_2_CH_2_CH_3_ and H-11); ^13^C NMR (125 MHz, CDCl_3_) δ 170.5, 154.6, 151.6, 148.0, 128.1, 114.3, 105.3, 83.8, 44.1, 32.3, 32.0, 31.2, 30.3, 27.8, 26.6 (2C), 22.4, 20.8, 18.6, 18.5, 14.1, 14.0; HRMS (ESI): *m/z* calcd for C_22_H_33_O_3_ [M + H]^+^ 345.2424, found 345.2423.

(*4aS,6aR,8aR,8bR,9aS,12S,12aS,14aR,14bR*)*-12-*(*furan-3-yl*)*-6,6,8a,12a-tetramethyldecahydro-1H,3H-oxireno*[*2,3-d*]*pyrano*[*4′,3′:3,3a*]*isobenzofuro*[*5,4-f*]*isochromene-3,8,10*(*6H,9aH*)*-trione* (Limonin, **9**)^49^. ^1^H NMR (500 MHz, CDCl_3_) δ 7.40–7.37 (m, 2H, H-21 and H-23), 6.32 (dd, J = 1.7, 0.7 Hz, 1H, H-22), 5.44 (s, 1H, H-17), 4.74 (d, J = 13.1 Hz, 1H, H-19a), 4.44 (d, J = 13.1 Hz, 1H, H-19b), 4.02 (s, 2H, H-1 and H-15), 2.95 (dd, J = 16.8, 3.8 Hz, 1H, H-2b), 2.83 (dd, J = 15.7, 14.7 Hz, 1H, H-6b), 2.66 (dd, J = 16.8, 1.8 Hz, 1H,H-2a), 2.53 (dd, J = 12.4, 2.8 Hz, 1H, H-9), 2.44 (dd, J = 14.5, 3.3 Hz, 1H, H-6a), 2.21 (dd, J = 15.8, 3.3 Hz, 1H, H-5), 1.92–1.77 (m, 2H, H-11), 1.50 (m, J = 23.6, 18.9, 8.8 Hz, 2H, H-12), 1.27 (s, 3H, H-25a), 1.15 (d, J = 2.1 Hz, 6H, H-18, 25b), 1.05 (s, 3H, H-24); ^13^C NMR (125 MHz, CDCl_3_) δ 206.3, 169.3, 166.8, 143.5, 141.3, 120.2, 109.9, 80.5, 79.4, 78.0, 65.9, 65.6, 60.8, 54.1, 51.6, 48.3, 46.2, 38.2, 36.6, 35.9, 31.0, 30.4, 21.6, 20.9, 19.1, 17.8; HRMS (ESI): m/z calcd for C_26_H_31_O_8_ [M + H]^+^ 471.2013, found 471.2010.

(*4aS,6aR,8aR,8bR,9aS,12R,12aS,14aR,14bR*)*-12-*(*2-bromofuran-3-yl*)*-6,6,8a,12a-tetramethyldecahydro-1H,3H-oxireno*[*2,3-d*]*pyrano*[*4*′*,3*′*:3,3a*]*isobenzofuro*[*5,4-f*]*isochromene-3,8,10*(*6H,9aH*)*-trione* (**10**). White powder; m.p. 172–173 °C; ${[{\rm{\alpha }}]}_{{\rm{D}}}^{30}$ = −13° (c 0.023, in CHCl_3_); ^1^H NMR (500 MHz, CDCl_3_) δ 7.43 (d, J = 2.1 Hz, 1H, H-23), 6.46 (d, J = 2.1 Hz, 1H, H-22), 5.39 (s, 1H, H-17), 4.74 (d, J = 13.1 Hz, 1H, H-19a), 4.44 (d, J = 13.1 Hz, 1H, H-19b), 4.07 (s, 1H, H-15), 4.00–4.11 (m, 1H, H-1), 2.94 (dd, J = 16.8, 3.8 Hz, 1H, H-2b), 2.87–2.80 (m, 1H, H-6b), 2.64 (dd, J = 16.7, 1.6 Hz, 1H, H-2a), 2.55–2.49 (m, 1H, H-9), 2.44 (dd, J = 14.5, 3.3 Hz, 1H, H-6a), 2.21 (dd, J = 15.8, 3.2 Hz, 1H, H-5), 1.91–1.84 (m, 7.5 Hz, 2H, H-11), 1.79–1.69 (m, 2H, H-12), 1.26–1.27 (m, 6H, H-18, 25a), 1.15 (s, 3H, H-25b), 1.07 (s, 3H, H-24); ^13^C NMR (125 MHz, CDCl_3_) δ 206.4, 169.3, 166.7, 144.8, 123.3, 119.0, 112.8, 80.5, 79.4, 76.7, 65.6, 65.5, 60.7, 54.2, 51.5, 48.4, 46.2, 39.6, 36.6, 35.8, 30.4, 30.0, 21.6, 21.2, 19.1, 18.0; HRMS (ESI): m/z calcd for C_26_H_30_O_8_Br [M + H]^+^ 549.1119, found 549.1109.

(*4aS,6aR,8aR,8bR,9aS,12R,12aS,14aR,14bR*)*-12-*(*2,5-dibromofuran-3-yl*)*-6,6,8a,12a-tetramethyldecahydro-1H,3H-oxireno*[*2,3-d*]*pyrano*[*4′,3′:3,3a*]*isobenzofuro*[*5,4-f*]*isochromene-3,8,10*(*6H,9aH*)*-trione* (**11**). White powder; m.p.193–194 °C; ${[{\rm{\alpha }}]}_{{\rm{D}}}^{30}$ = 11° (c 0.027, in CHCl_3_); ^1^H NMR (500 MHz, CDCl_3_) δ 6.40 (s, 1H, H-22), 5.34 (s, 1H, H-17), 4.74 (d, J = 13.1 Hz, 1H, H-19a), 4.44 (d, J = 13.1 Hz, 1H, H-19b), 4.07 (s, 1H, H-15), 4.00 (m, 1H, H-1), 2.95 (dd, J = 16.8, 3.7 Hz, 1H, H-2b), 2.89–2.78 (m, 1H, H-6b), 2.65 (dd, J = 16.8, 1.5 Hz, 1H, H-2a), 2.52–2.49 (m, 1H, H-9), 2.45 (dd, J = 14.5, 3.2 Hz, 1H, H-6a), 2.21 (dd, J = 15.8, 3.1 Hz, 1H, H-5), 1.92–1.85 (m,2H, H-11), 1.79–1.71 (m, 2H, H-12), 1.24 (d, J = 6.0 Hz, 6H, H-18, 25a), 1.16 (s, 3H, H-25b), 1.07 (s, 3H, H-24); ^13^C NMR (125 MHz, CDCl_3_) δ 206.3, 169.2, 166.3, 123.5, 122.9, 122.2, 114.5, 80.6, 79.4, 77.5, 76.5, 60.8, 54.2, 51.5, 48.4, 46.2, 39.6, 36.6, 35.8, 30.4, 30.0, 21.6, 21.2, 19.1, 18.0; HRMS (ESI): m/z calcd for C_26_H_29_O_8_Br_2_ [M + H]^+^ 627.6224, found 549.6214.

#### Biological assay

The insecticidal activity of **1-7, 3a**-**g, 4a**-**c, 5a, 5c**-**h** and **6a, 6b** was tested as the mortality rate values by using the leaf-dipping method against the pre-third-instar larvae of *M. separata* using the reported procedure^32^. For each sample, a total of 24 pre-third-instar larvae (6 larvae per group) were used. Each treatment was performed four times. Acetone solutions of **1-7**, **3a**-**g**, **4a**-**c**, **5a**, **5c**-**h**, **6a, 6b** and toosendanin (positive control) were prepared at 1 mg/mL. Fresh wheat leaf discs (1 × 1 cm) were dipped into the corresponding solution for 3 s, then taken out and dried. Leaf discs treated with acetone alone were used as a blank control group. Several pieces of treated leaf discs were kept in each 6 well plate which was then placed in a conditioned room (25 ± 2 °C, 65–80% relative humidity (RH), 12 h/12 h (light/dark). Once the treated leaves were consumed, the corresponding ones were added to the dish. After 2 days, untreated fresh leaves were added to the all dish until the adult pupae emergence. The corrected mortality rates of the tested compounds against *M. separata* Walker were calculated in three different periods by the following formula:

$${\rm{corrected}}\,{\rm{mortality}}\,{\rm{rate}}\,( \% )=\frac{{\rm{mortality}}\,{\rm{rate}}\,{\rm{of}}\,{\rm{test}}\,-\,{\rm{mortality}}\,{\rm{rate}}\,{\rm{of}}\,{\rm{control}}}{100 \% \,-\,{\rm{mortality}}\,{\rm{rate}}\,{\rm{of}}\,{\rm{control}}}\times 100 \% $$

## Electronic supplementary material

Supplementary Information
