# Supplementary material for: Furan-Site Bromination and Transformations of Fraxinellone as Insecticidal Agents Against Mythimna separata Walker
Source: Sci Rep. 2018 May 30;8:8372. doi: 10.1038/s41598-018-26747-0 (PMC5976728; doi:10.1038/s41598-018-26747-0)

Supporting Information

Furan-Site Bromination and Transformations of Fraxinellone as Insecticidal Agents Against *Mythimna separata* Walker

Qing-Miao Dong[1,†], Shuai Dong[1,†], Cheng Shen[1], Qing-Hao Cao[1], Ming-Yu Song[1], Qiu-Rui He[1], Xiao-Ling Wang[2,*], Xiao-Jun Yang[3], Jiang-Jiang Tang[1,*], Jin-Ming Gao[1,2]

[1]Shaanxi Key Laboratory of Natural Products & Chemical Biology, College of Chemistry & Pharmacy, Northwest A&F University, Yangling, 712100, Shaanxi, P. R. China

[2]Shaanxi Key Laboratory of Phytochemistry, Baoji University of Arts and Sciences, Baoji 721007, P. R. China

[3]School of Chemistry & Chemical Engineering, Yanan University, Yanan 716000, Shaanxi, P. R. China

[†] These authors contributed equally to this work.

*Corresponding authors.

E-mail: tangjiang11@nwafu.edu.cn; xlwangwang@163.com

Content list

1. Control studies: Figure S1-8……………………………………………..S2-S10

2. Water stability: Figure S9…………………………………………… …….S11, S12

3. 1D/2D NMR..S13-S44

4. (HR)-ESI-MS spectra...S45-S58

1. Control studies

1). Investigation of bromination of 2

As shown in Table S1, bromination of 2 at room temperature gave the monobromination product **3** in 32% yield after 24 hours while lower temperature diminished its yield significantly (entries 1-3). The influence of the loading of NBS on the distribution of products was then studied. When the amount of NBS was increased to 1.4 equivalent, the dibromides **4** was obtained as the main product (entry 4). However, the monobromide **3** could be separated in comparative higher yield than **4** even when 2 equivalents of NBS was used if the reaction was stopped at 16 h, which indicated that the distribution of products was the result of both the concentration of Br^+ and the reaction time. Probably, **4** was the futher bromination product of **3**. Considering that the Br^+ mechanism was involved in the bromination and Br_2 of low concentration was continuously released with NBS, we tested Br_2 as the brominating reagent directly in the reaction. As we expected, the reaction was finished much more quickly (within one hour) and **3** was obtained in 28% yield. But higher concentration of Br_2 (3 eq) diminished its yield completely while the yield of **4** increased (entries 6 and 7).We then assumed that the combination of NBS (as brominating reagent) and Br_2 (as catalyst) would increase the reaction rate without damage of the yield of **3**. To our delight, this strategy increased the yield significantly, such as the entry 9 (54% for **3** and 26% for **4**,80% in total) and entry 11 (51% for **3** and 21% for **4**, 72% in total). Further studies revealed that 0.4 eq of Br_2 with 1.05 eq of NBS under room temperature was optimal.

A Schlenk tube was charged with **2** (0.02 mmol, 1 equiv) and *N*-bromosuccinimide (NBS, 0.04 mmol, 2 equiv). Then the dichloromethane (DCM, 0.5 mL) were added to this tube. The reaction mixture was stirred at 25 ^{o}C. After the reaction was completed, 2 mL of H_2O was added. The mixture was extracted with DCM (5 mL × 4) and evaporated under reduced pressure. The crude reaction mixture was diluted with 0.6 mL of $CDCl_3$ and analyzed by ^{1}H NMR (Table S1 and Figure S2, entry 5). The other two fractions were prepared by the same procedure, but Br_2 in DCM (1 or 3 equiv, 1 mol/L) was added to the tube instead of NBS (Table S1 and Figure S2, entry 6 and 7).

Table S1.Investigation of Bromination of 2

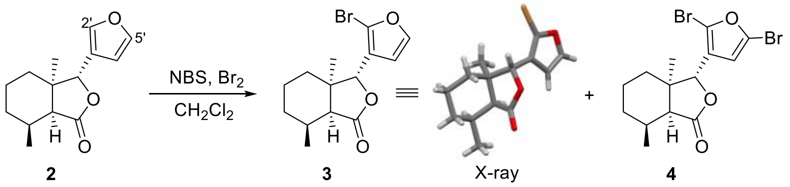

entry	T (°C)	NBS (equiv)	Br_2 (equiv)	t (h)	yield (%)[a]	
					**3**[b]	**4**
1	-10	1.05	0	24	-	-
2	0	1.05	0	24	23	6.2
3	RT	1.05	0	24	32	7.3
4	RT	1.4	0	24	17	23
5[c]	RT	2	0	16	25	23
6[c]	RT	0	1	1	28	3
7[c]	RT	0	3	1	-	37
8	RT	1.05	0.3	1	45	19
9	RT	1.05	0.4	1	54	26
10	RT	1.05	0.5	1	28	38

11	40	1.05	0.4	1	51	21
12[c]	RT	1.05	0.4	1	52	24

[a]Isolated yield. Reactions were performed using 0.02 mmol of **2** in CH_2Cl_2. Full conversion was achieved in all cases except entries 1 and 2; [b]The CCDC number of compound **3** (X-ray) is 1549252; [c]Yield was determined by ^1H NMR analysis ofthe reactionmixture in 0.6 mL $CDCl_3$ (See the Figure S1 and S2 in the Supporting Information);[d]1.3 g of **2** was carried out.

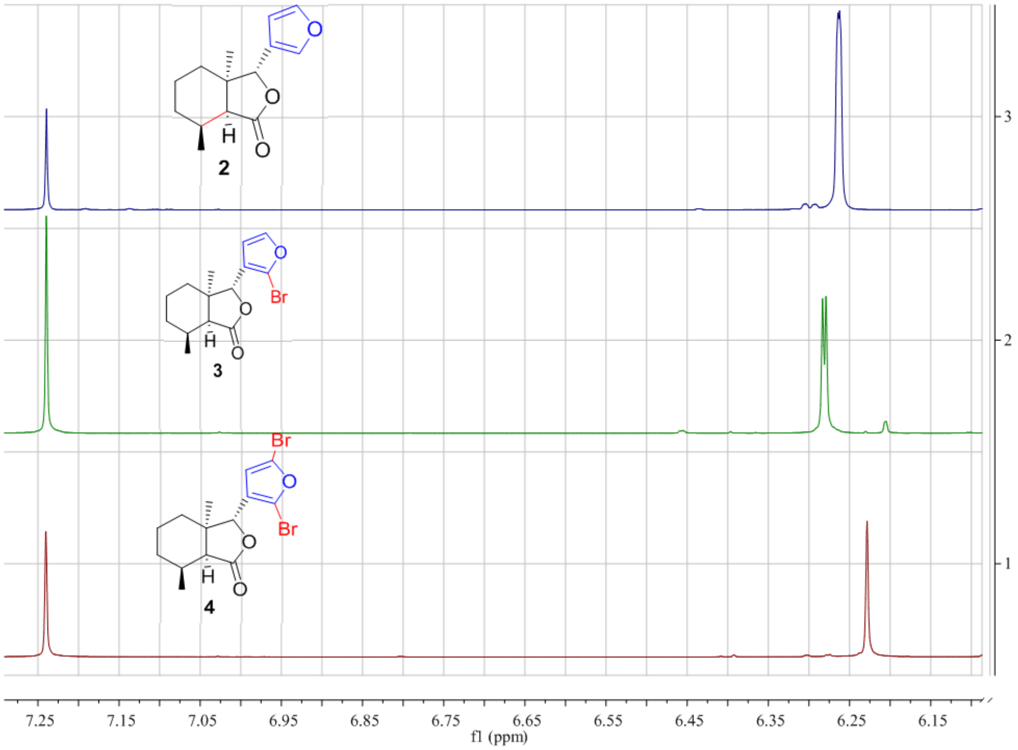

Figure S1. ^1H NMR spectra of compounds **2**, **3** and **4** in $CDCl_3$

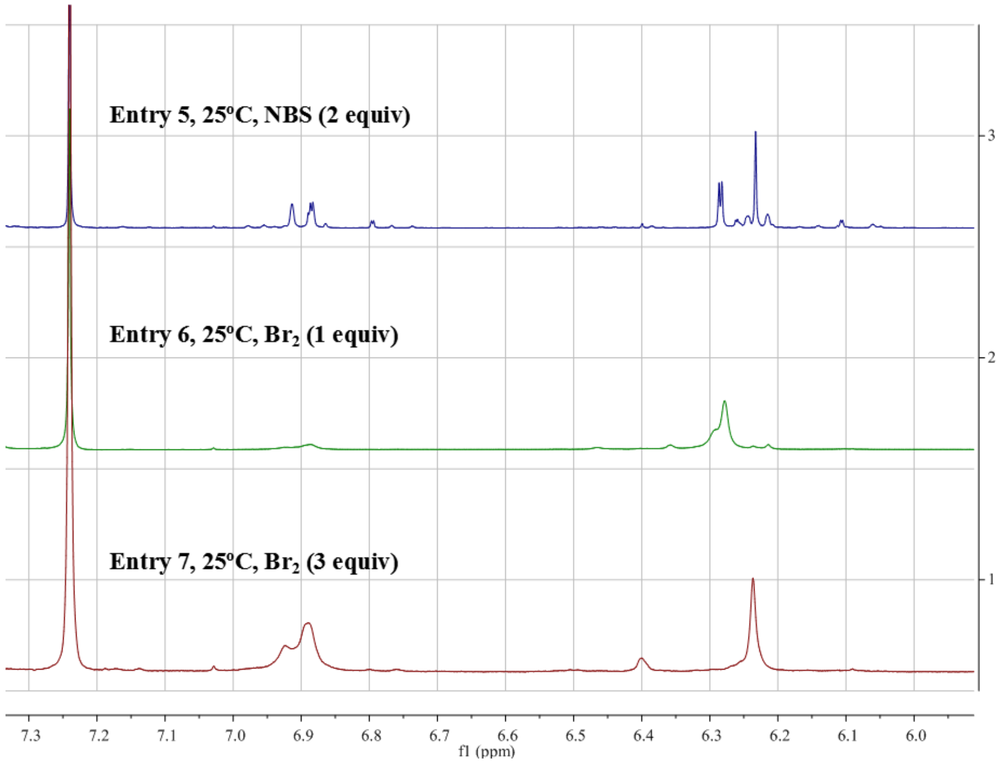

Figure S2. Investigation of bromination of **2** with NBS or Br_2: crude ^{1}H NMR spectra of reaction mixture in 0.6 mL $CDCl_3$. (The yields were listed in Table S1, entries 5-7)

2). Investigation of bromination of 1

A Schlenk tube was charged with fraxinellone (**1**) (0.02 mmol, 1.0 equiv) and NBS (1.05/1.4/2 equiv). Then the DCM (0.5 mL) were added to this tube. The reaction mixture was stirred at corresponding temperature (0/25/40 ^{o}C). After the reaction was completed, 2.0 mL of H_2O was added. The mixture was extracted with DCM (5 mL × 4) and evaporated under reduced pressure. The crude reaction mixture was diluted with 0.6 mL of $CDCl_3$ and analyzed by ^{1}H NMR (Table 2 and Figure S4, entries 3-6).

The other fractions were prepared by the same procedure, but the reaction mixture was kept in the dark (Table S2 and Figure S5, entries 7-11).

Table S2. Investigation of Bromination of Fraxinellone (1)

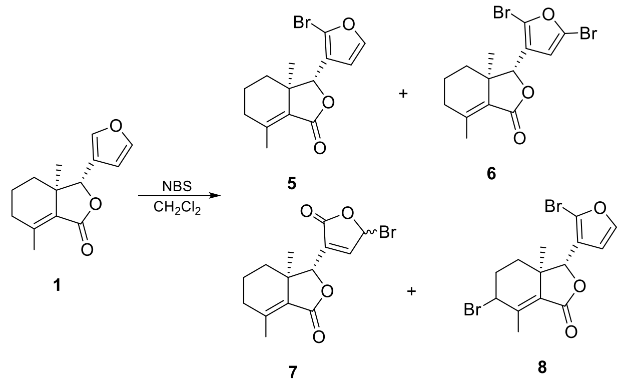

Entry	T (℃)	NBS (equiv)	Br_2 (equiv)	t (h)	yield (%)[a]			
					**5**[b]	**6**	**7**	**8**
1[c]	RT	1.05	0.4	1	47	5	25	-
2[c]	RT	1.2	0.2	1	50	11	11	-
3	RT	1.05	0	5	35	3	19	13
4	RT	1.4	0	5	23	5	8	20
5	RT	2	0	2	13	2	9	59
6	0	1.4	0	5	22	6	55	3
7	0	1.05	0	2	30	5	34	-
8	0	1.4	0	5	27	9	55	-
9	0	2	0	5	-	16	47	-
10	RT	1.4	0	5	41	22	32	-
11	40	1.4	0	3	62	38	-	-
12[d]	40	1.4	0	3	60	34	-	-

[a]Reactions were performed using 0.02 mmol of **1** in CH_2Cl_2. Yield was determined by ^1H NMR analysis of the reaction mixture in 0.6 mL $CDCl_3$ (See the Figure S3, S4 and S5 in the Supporting Information). Entries 1-6 were carried out under CFL (household compact fluorescent lamp, 28 W), and entries 7-12 were carried out in the dark; [b]The CCDC number of compound **5** (X-ray) is 1549253; [c]Isolated yield; [d]1.1 g of compound **1** was carried out.

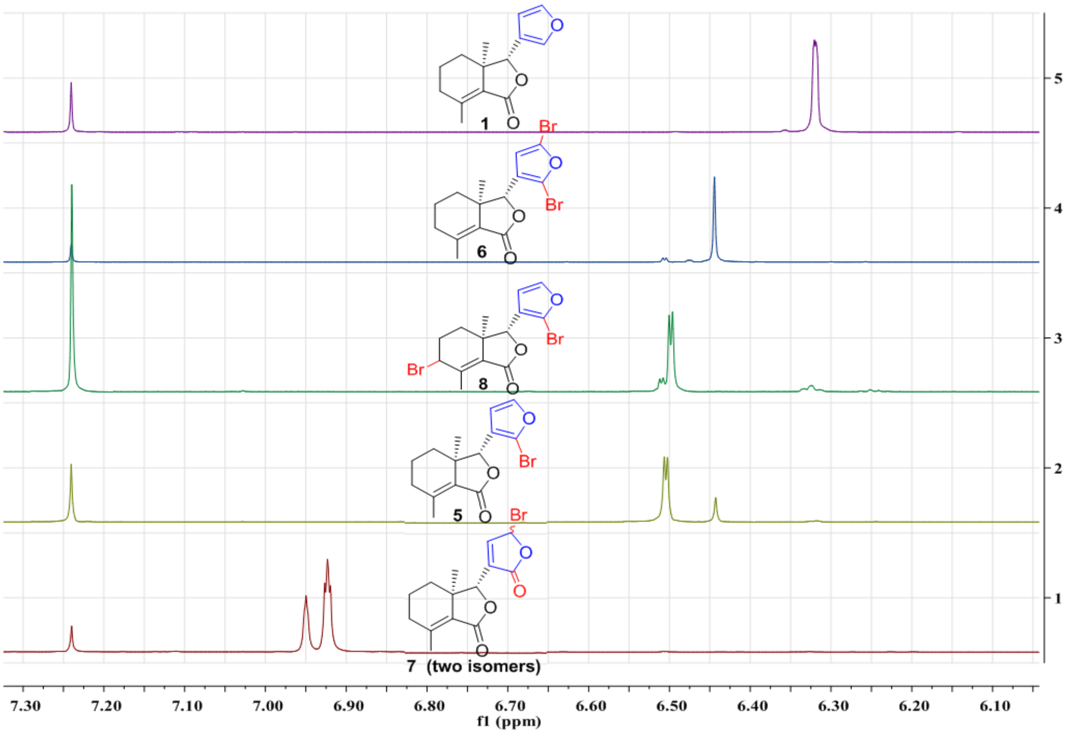

Figure S3. ^{1}H NMR spectra of compounds **1**, **5**, **6**, **7** and **8** in $CDCl_3$

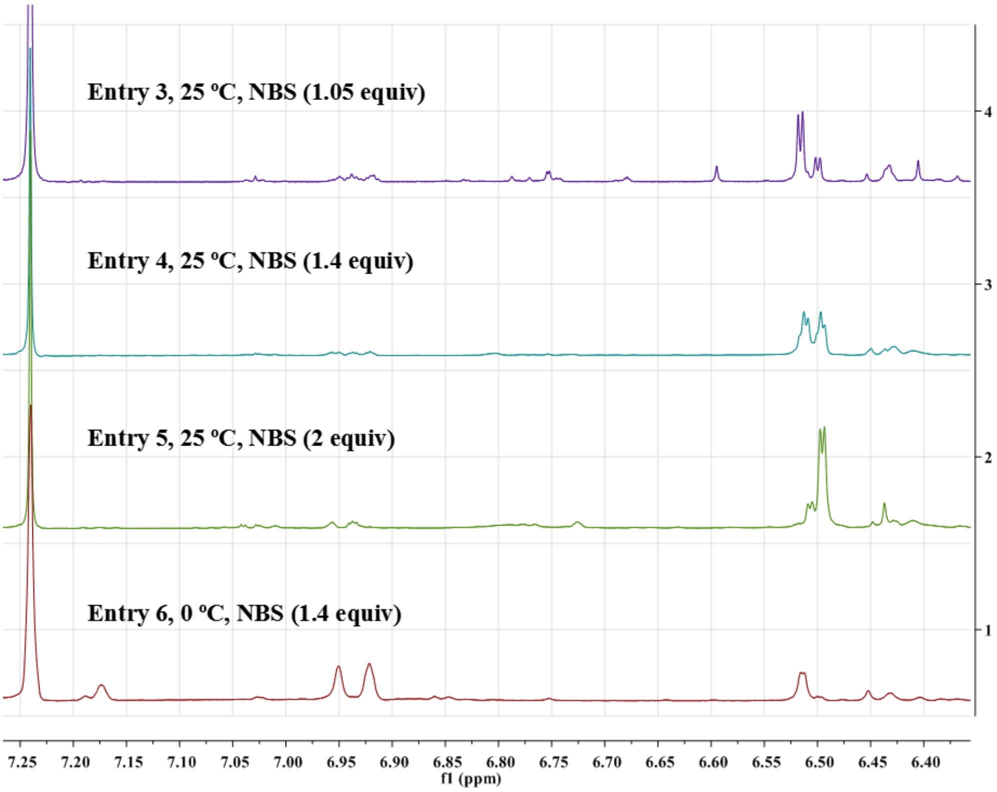

Figure S4. Investigation of bromination of fraxinellone with the NBS: crude ^{1}H NMR spectra of reaction mixture in 0.6 mL $CDCl_3$ (The yields were listed in Table S2, entries 3-6).

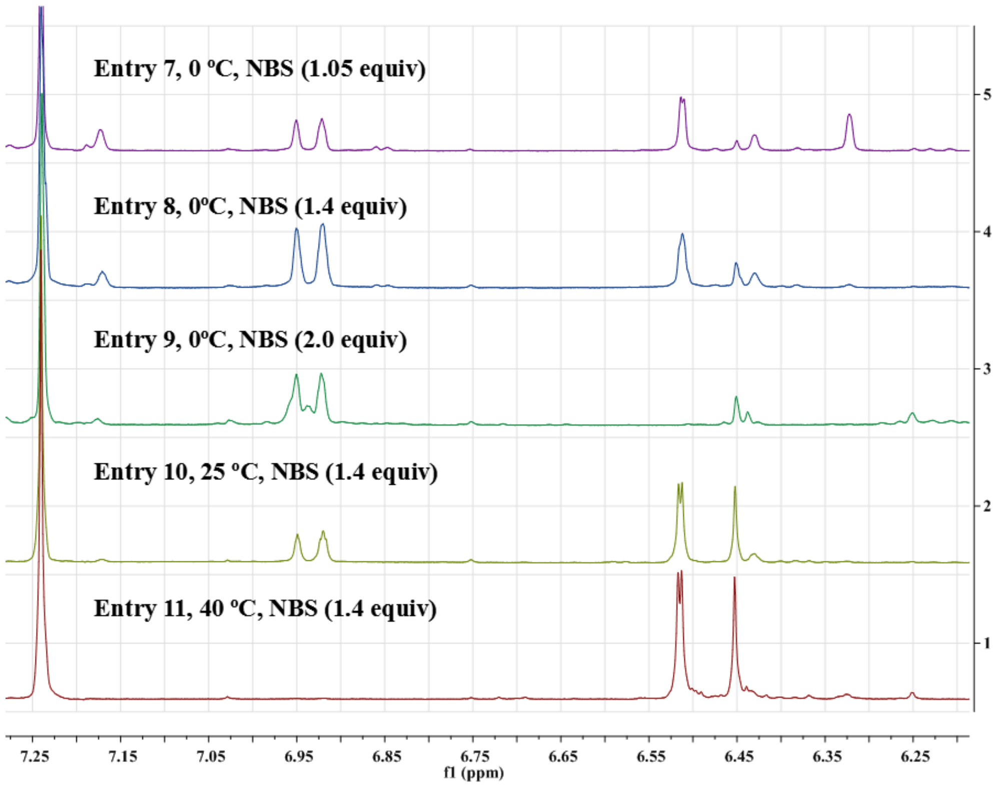

Figure S5. Investigation of bromination of fraxinellone with the NBS in the dark: crude ^{1}H NMR spectra of reaction mixture in 0.6 mL $CDCl_3$ (The yields were listed in Table S2, entries 7-11).

3). Investigation of bromination of fraxinellone (1) with different brominating agents

A Schlenk tube was charged with fraxinellone (**1**) (0.02 mmol, 1.0 equiv) and bromination agents. Then the DCM (0.5 mL) were added to this tube. The reaction mixture was stirred at 40 ^{o}C and kept in the dark. After the reaction was completed, 2.0 mL of H_2O was added. The mixture was extracted with DCM (5 mL × 4) and evaporated under reduced pressure. The crude reaction mixture was diluted with 0.6 mL of $CDCl_3$ and analyzed by ^{1}H NMR (Table S2 and Figure S6, entries 1-5). In gram-scale, 800 mg of **1** was carried out.

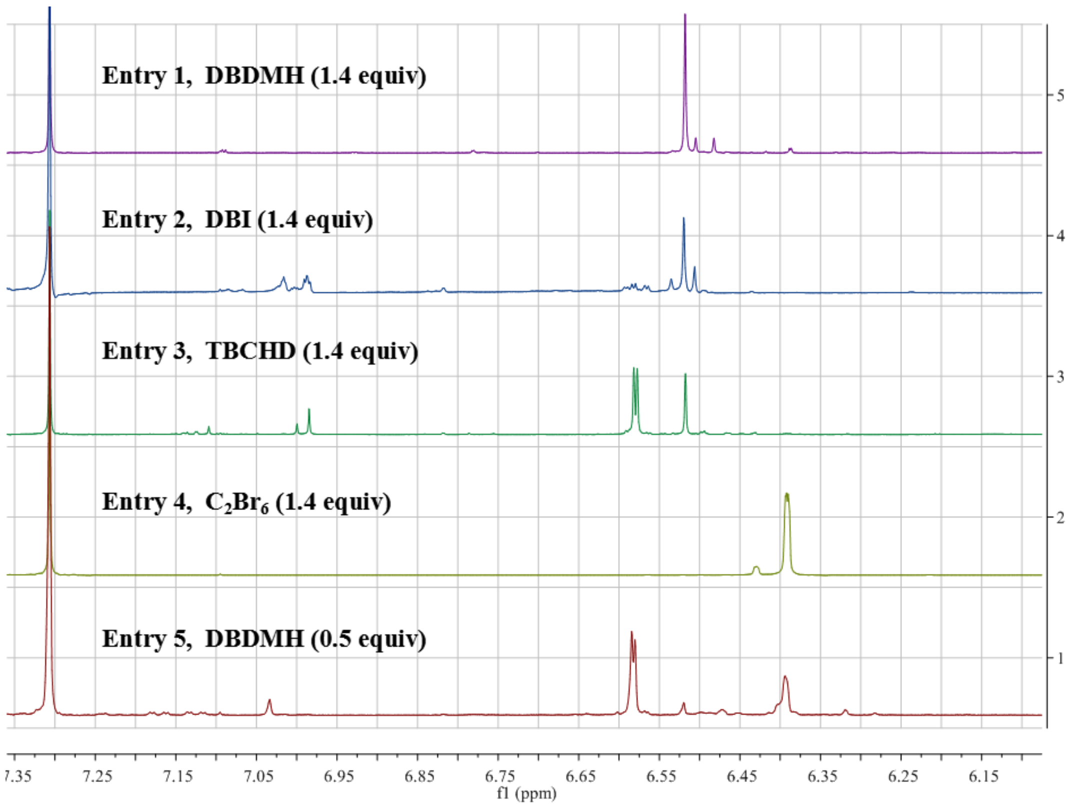

Figure S6. Investigation of bromination of **1** with different reagents: crude ^{1}H NMR spectra of reaction mixture in 0.6 mL $CDCl_3$ (The yields were listed in Table S2, entries 1-5).

4). Investigation of bromination of limonin (9)

A Schlenk tube was charged with limonin (**9**) (0.02 mmol, 1.0 equiv) and NBS or 1,3-dibromo-5,5-dimethylhydantoin (DBDMH). Then DCM (0.5 mL) were added to this tube. The reaction mixture was stirred at 40 ^{o}C and kept in the dark. After the reaction was completed, 2.0 mL of H_2O was added. The mixture was extracted with DCM (5 mL × 4) and evaporated under reduced pressure. The crude reaction mixture was diluted with 0.6 mL of $CDCl_3$ and analyzed by ^{1}H NMR (Table 3, entries 1-3).

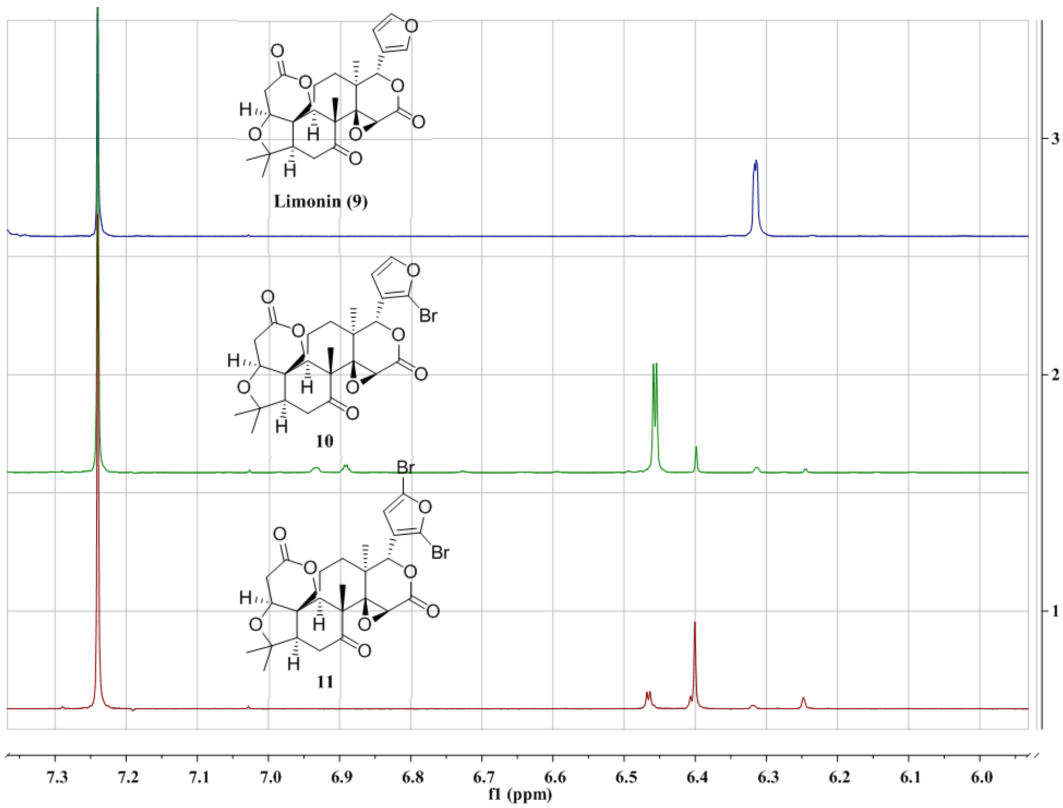

Figure S7. ^{1}H NMR spectra of compounds **9, 10** and **11** in $CDCl_3$

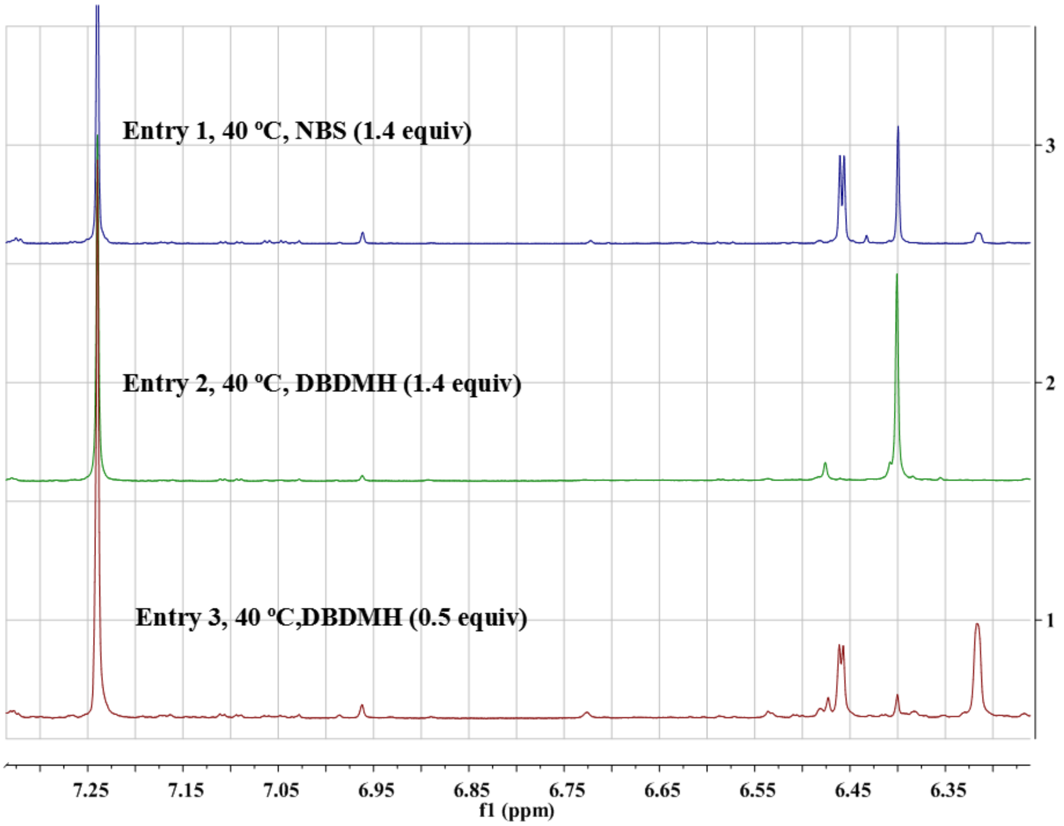

Figure S8. Investigation of bromination of limonin (**9**): crude ^{1}H NMR spectra of reaction mixture in 0.6 mL $CDCl_3$ (The yields were listed in Table 3, entries 1-3).

2. Water stability

Representative compounds (**1**, **2**, **3g** and **5g**) were prepared in DMSO (20 mM) as stock solutions. For water stability study, a stirred 950 μL solution of PBS (10 mM, pH 7.4)-acetonitrile (ACN) in an open vial was added 50 μL solution of sample stock solution. Resulting reaction mixture was monitored in indicating time by analytical HPLC. HPLC was performed on a Waters 1525 series with an Agilent TC-C18 column and UV (PDA) detection at the wavelength of compounds.

Representative compounds:

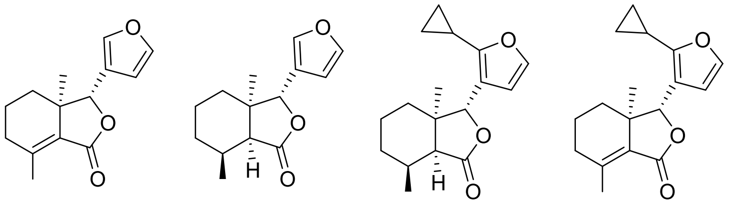

1 **2** **3g** **5g**

Absorption Peak of Compound **1** at 254 nm Wavelength at Different Time Points

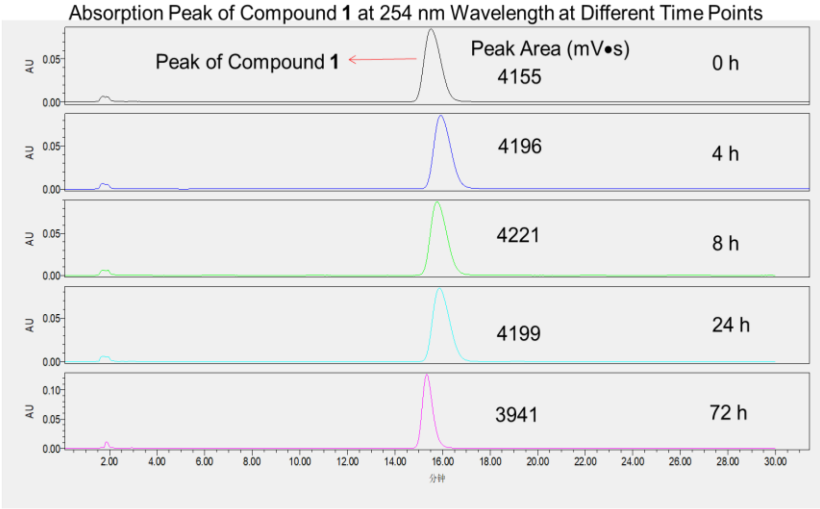

Absorption Peak of Compound **2** at 210 nm Wavelength at Different Time Points

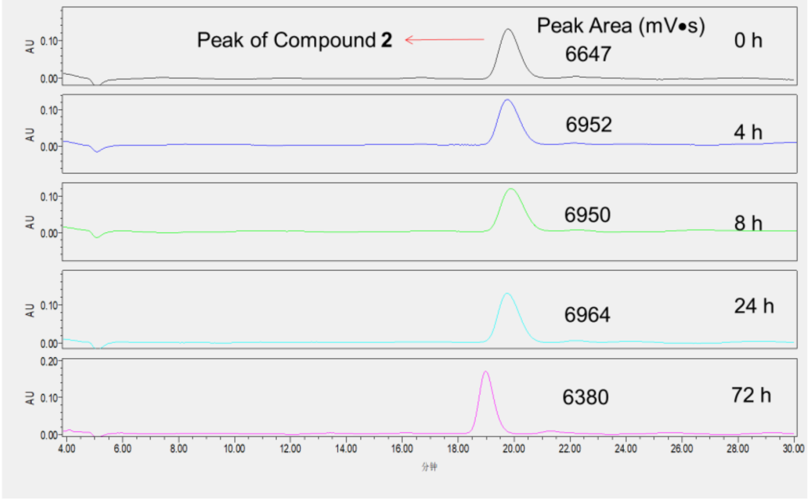

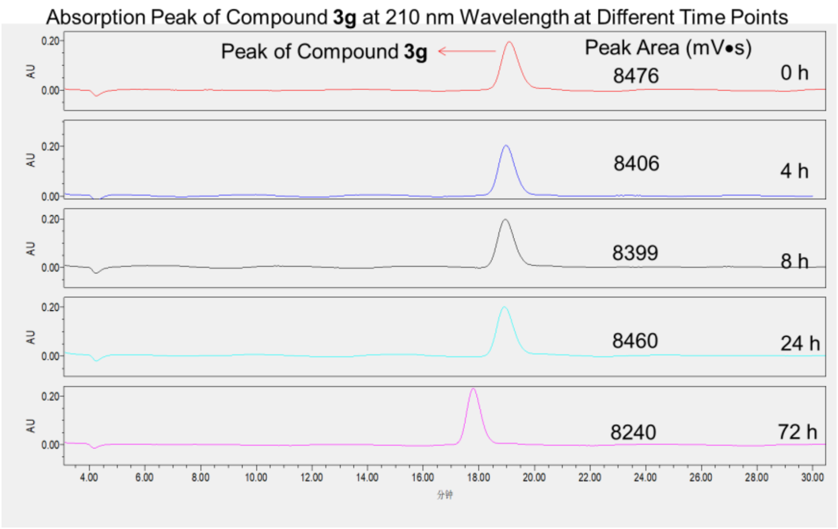

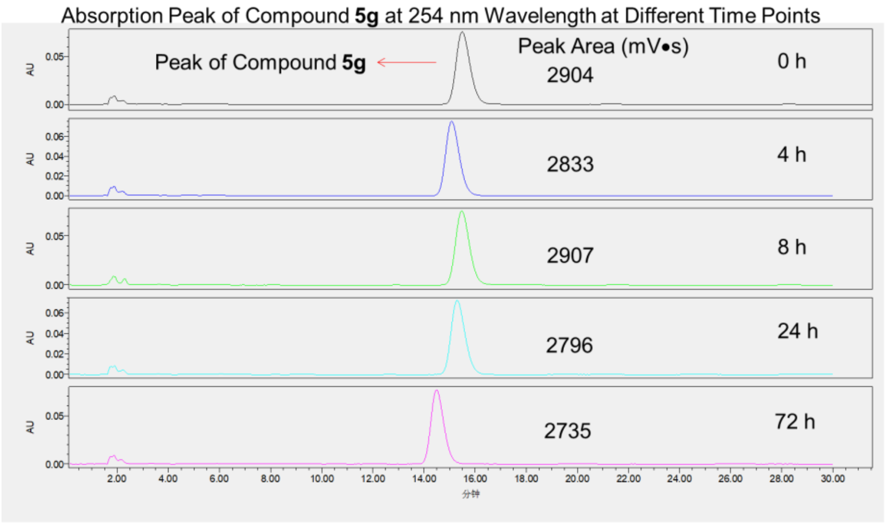

Figure S9. Water stability of representative compounds (**1**, **2**, **3g** and **5g**) in a solution of PBS/ACN (2:1) at room temperature using HPLC.

3. 1D/2D NMR spectra

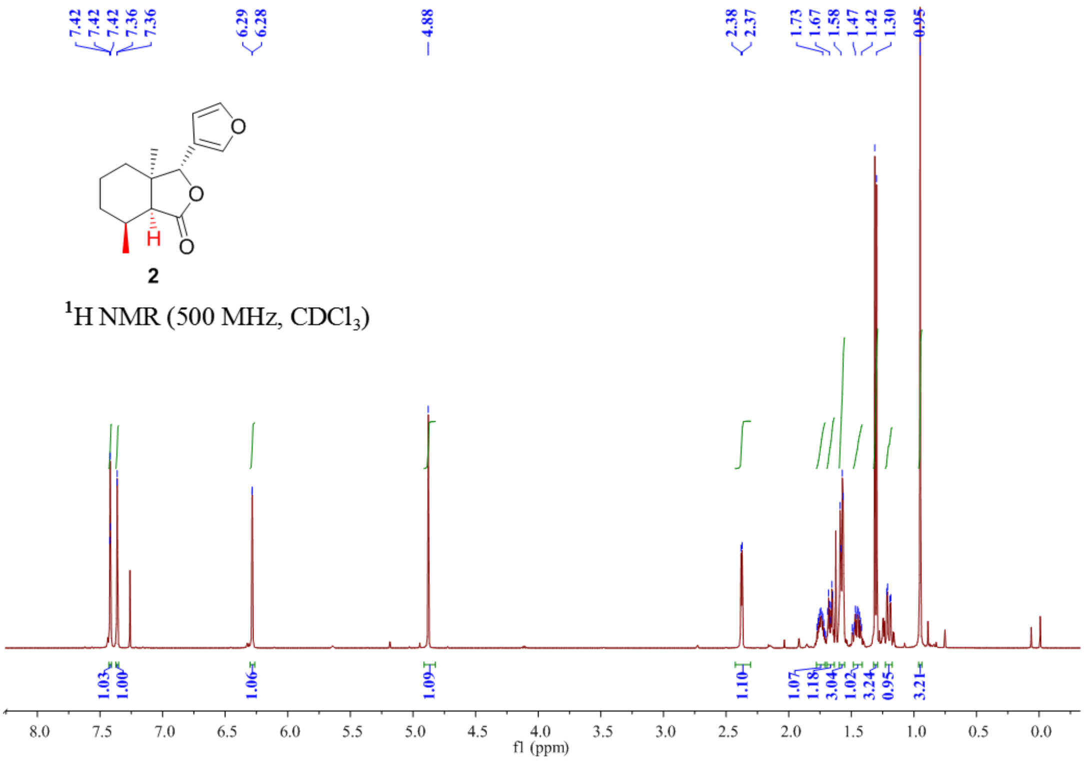

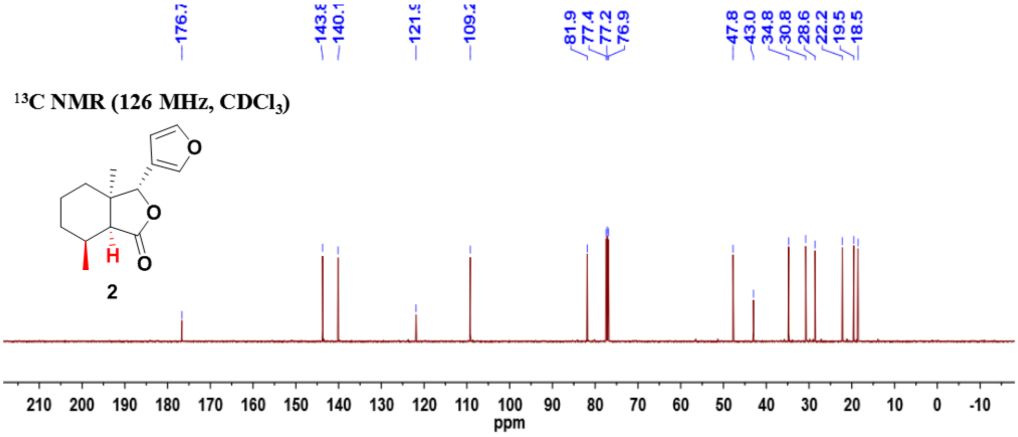

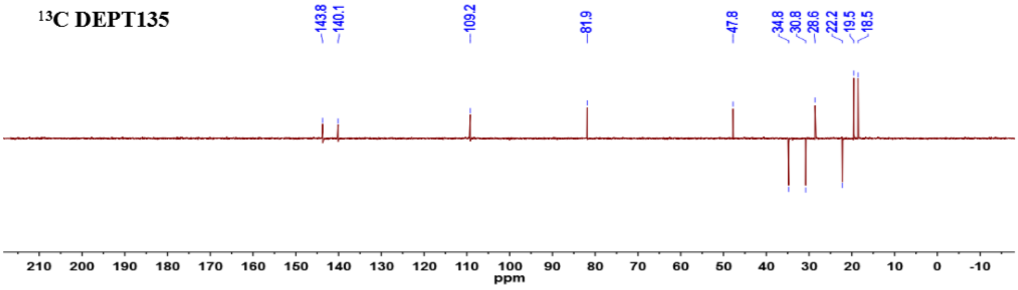

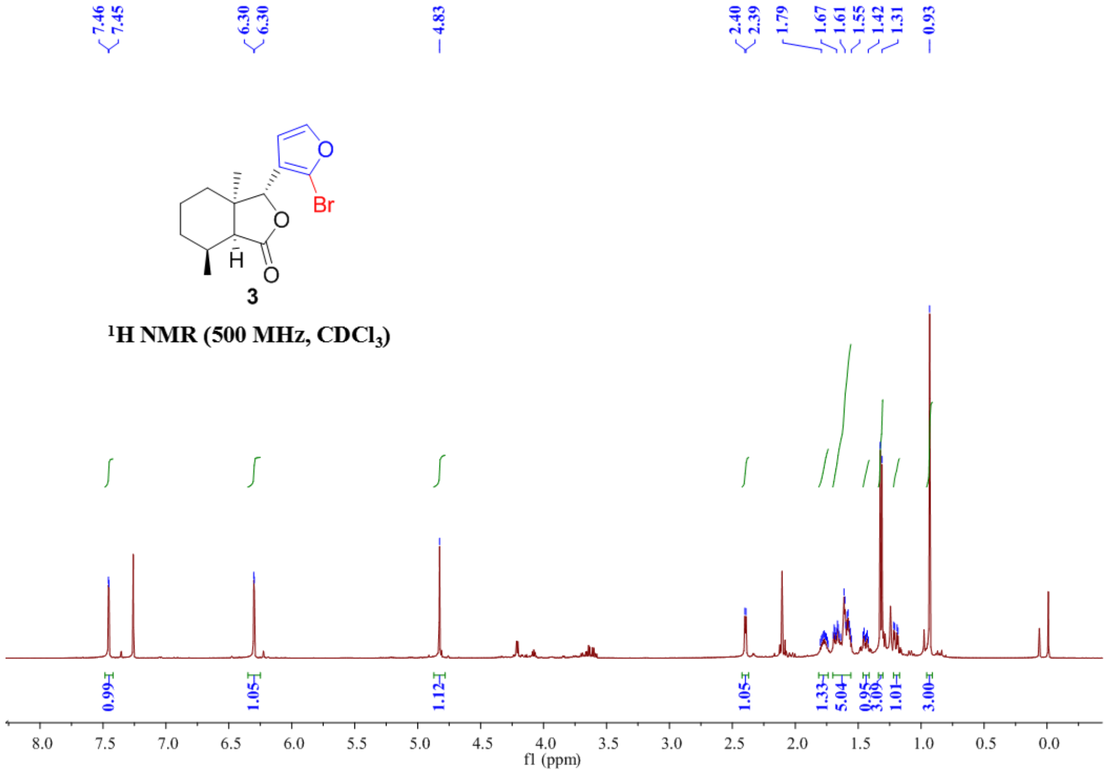
7.46
7.45
6.30
6.30
4.83
2.40
2.39
1.79
1.67
1.61
1.55
1.42
1.31
0.93
Br
O
H
3
1H NMR (500 MHz, CDCl3)
0.99
1.05
1.12
1.05
1.33
5.04
0.95
3.09
1.01
3.00
8.0
7.5
7.0
6.5
6.0
5.5
5.0
4.5
4.0
3.5
3.0
2.5
2.0
1.5
1.0
0.5
0.0
f1 (ppm)

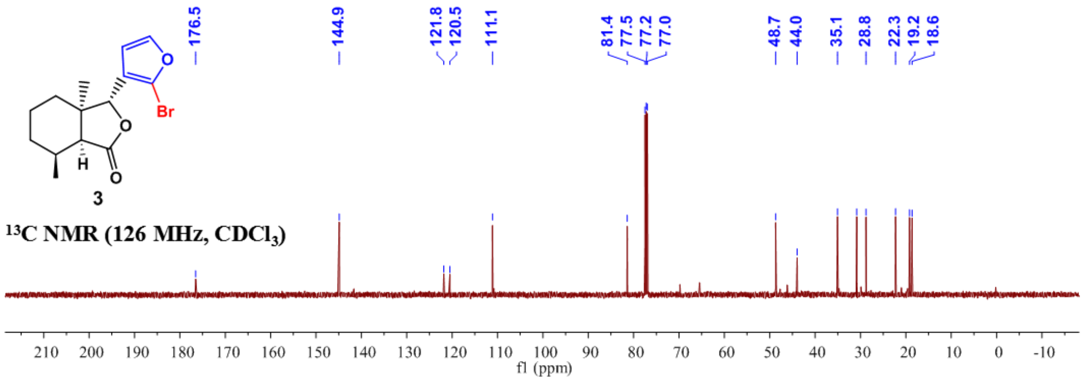
176.5
144.9
121.8
120.5
111.1
81.4
77.5
77.2
77.0
48.7
44.0
35.1
28.8
22.3
19.2
18.6
Br
O
H
3
13C NMR (126 MHz, CDCl3)
210
200
190
180
170
160
150
140
130
120
110
100
90
80
70
60
50
40
30
20
10
0
-10
f1 (ppm)

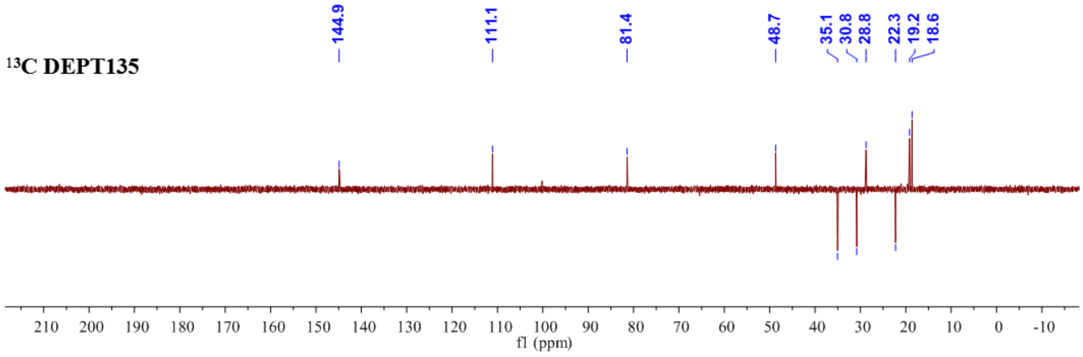
144.9
111.1
81.4
48.7
35.1
30.8
28.8
22.3
19.2
18.6
13C DEPT135
210
200
190
180
170
160
150
140
130
120
110
100
90
80
70
60
50
40
30
20
10
0
-10
f1 (ppm)

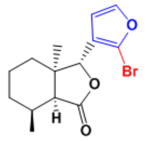
O
Br
O
H
O

3

HMBC

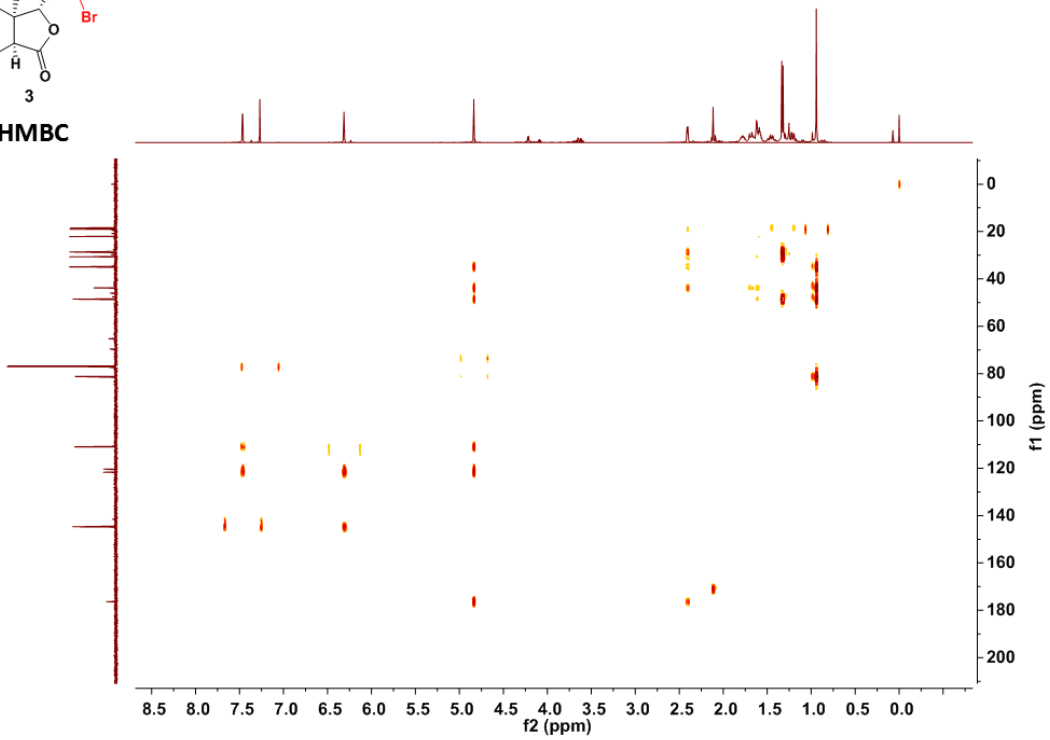
f2 (ppm)
f1 (ppm)
8.5 8.0 7.5 7.0 6.5 6.0 5.5 5.0 4.5 4.0 3.5 3.0 2.5 2.0 1.5 1.0 0.5 0.0
0 20 40 60 80 100 120 140 160 180 200

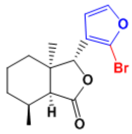
O
Br
O
H
O

3

HSQC

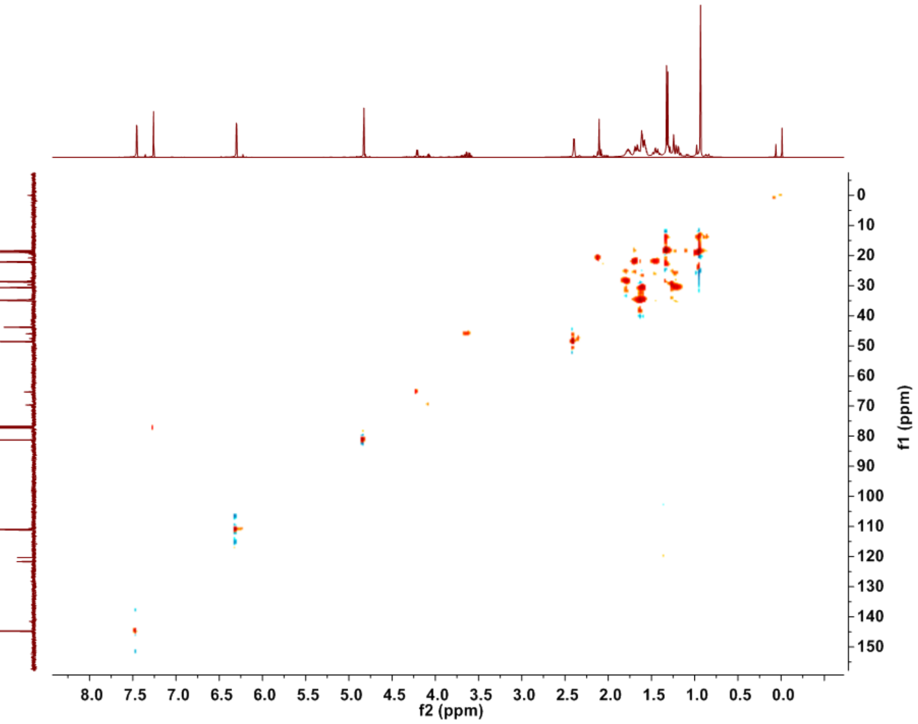
f2 (ppm)
f1 (ppm)
8.0 7.5 7.0 6.5 6.0 5.5 5.0 4.5 4.0 3.5 3.0 2.5 2.0 1.5 1.0 0.5 0.0
0 10 20 30 40 50 60 70 80 90 100 110 120 130 140 150

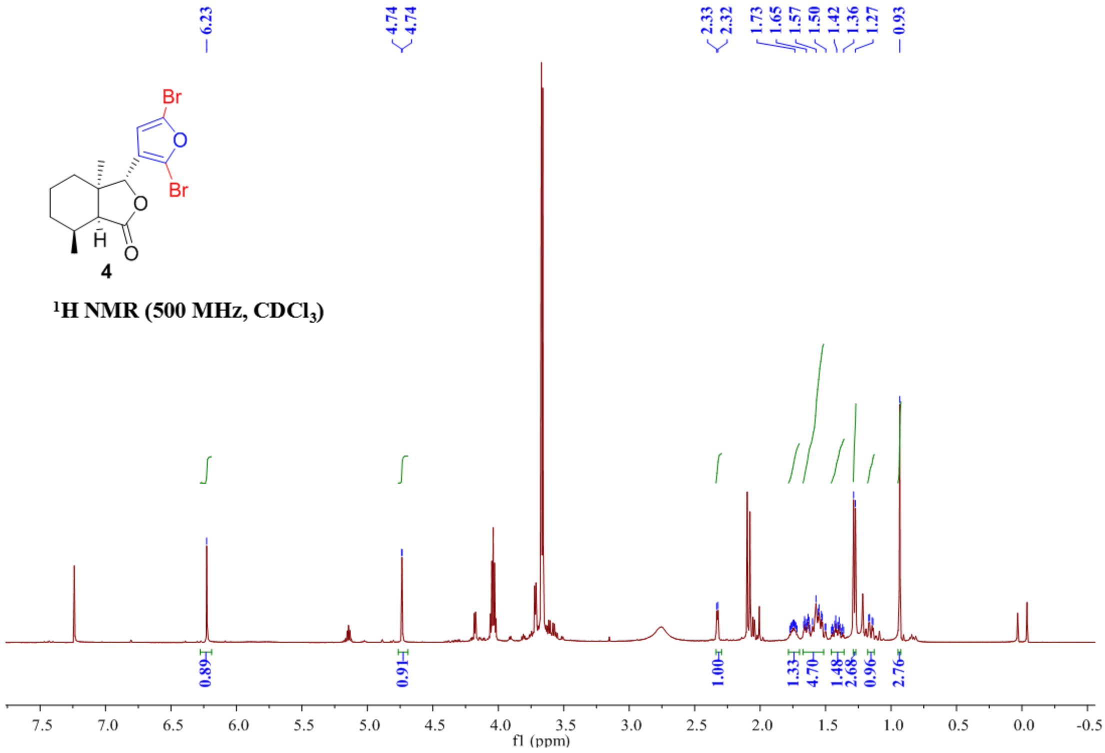

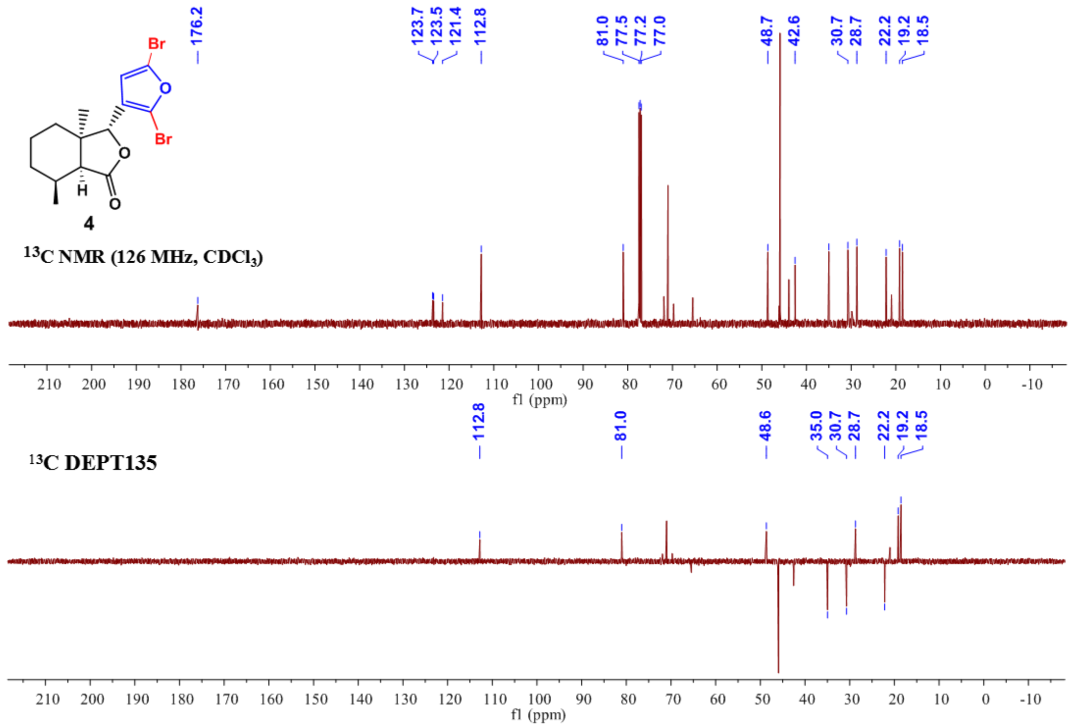

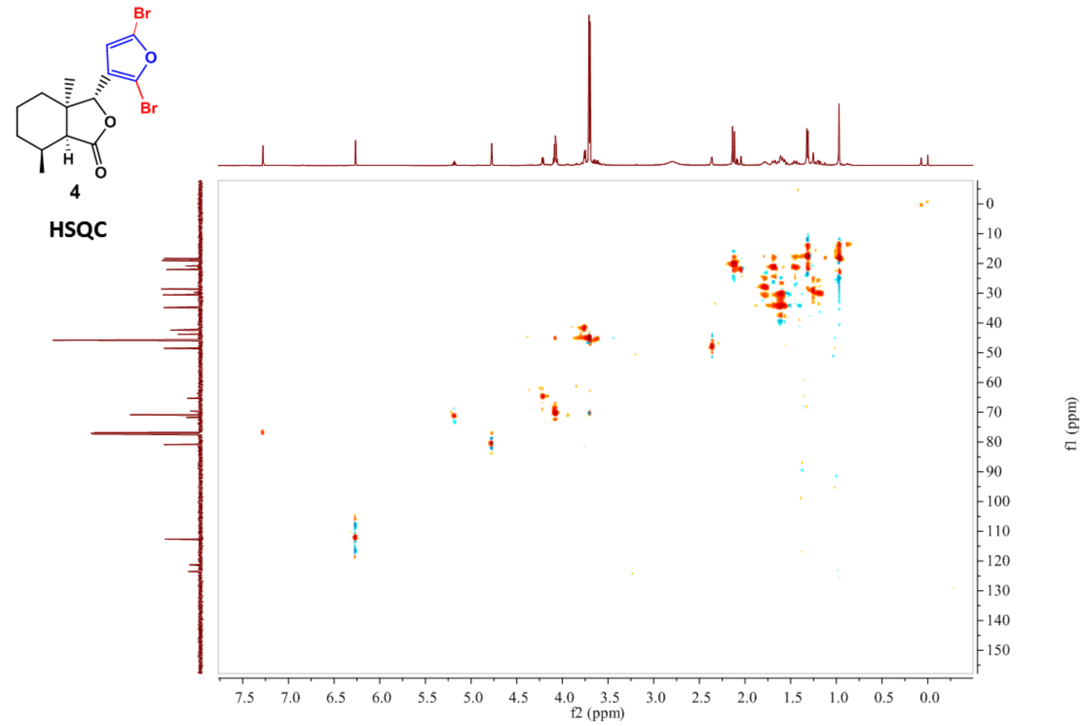

4
HSQC
f2 (ppm)
f1 (ppm)

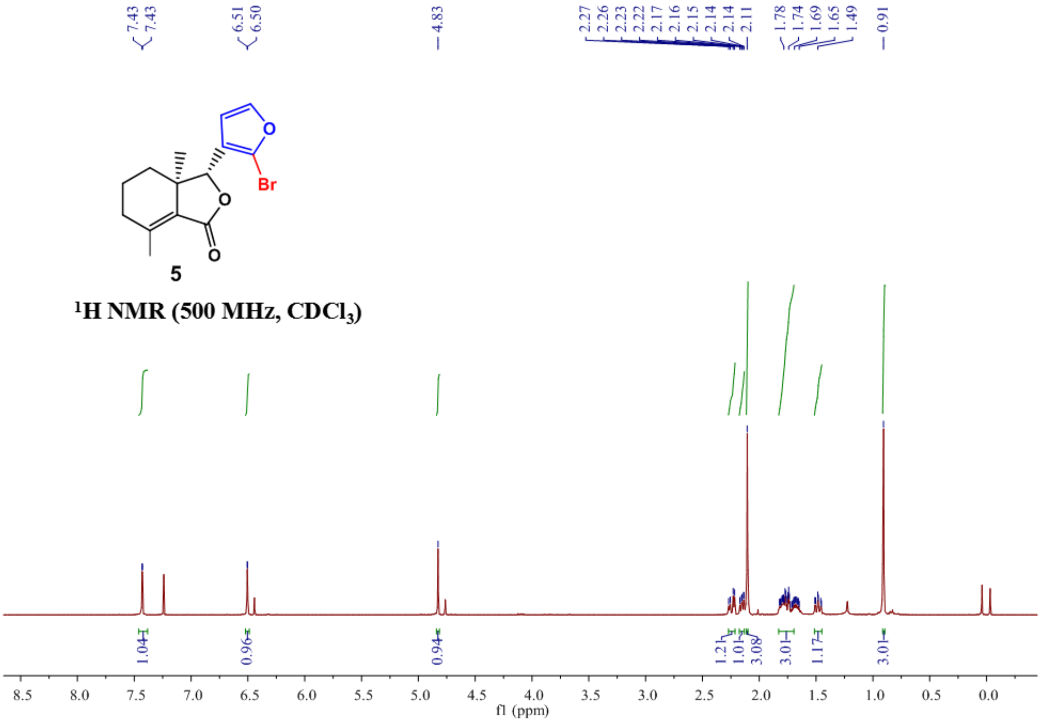

7.43
7.43
6.51
6.50
4.83
2.27
2.26
2.23
2.22
2.17
2.16
2.15
2.14
2.14
2.11
1.78
1.74
1.69
1.65
1.49
0.91
5
¹H NMR (500 MHz, CDCl₃)
1.04
0.96
0.94
1.21
1.01
3.08
3.01
1.17
3.01
f1 (ppm)

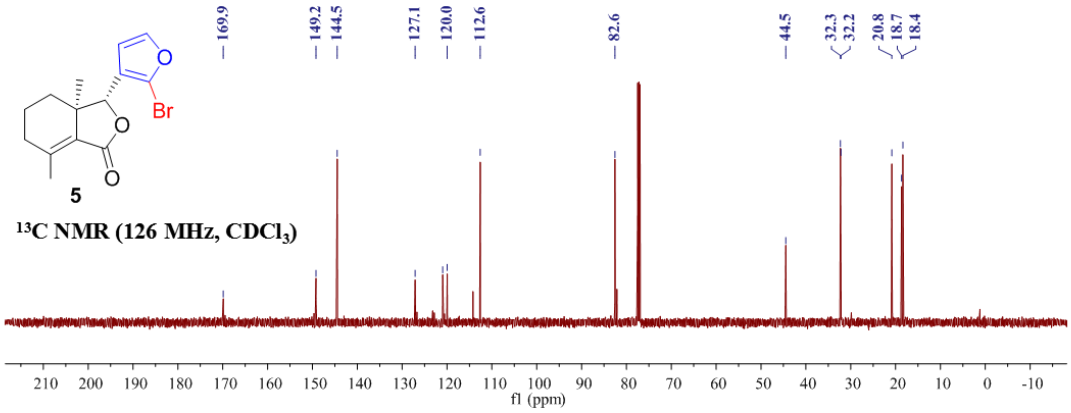

5
13C NMR (126 MHz, CDCl3)
169.9
149.2
144.5
127.1
120.0
112.6
82.6
44.5
32.3
32.2
20.8
18.7
18.4
f1 (ppm)

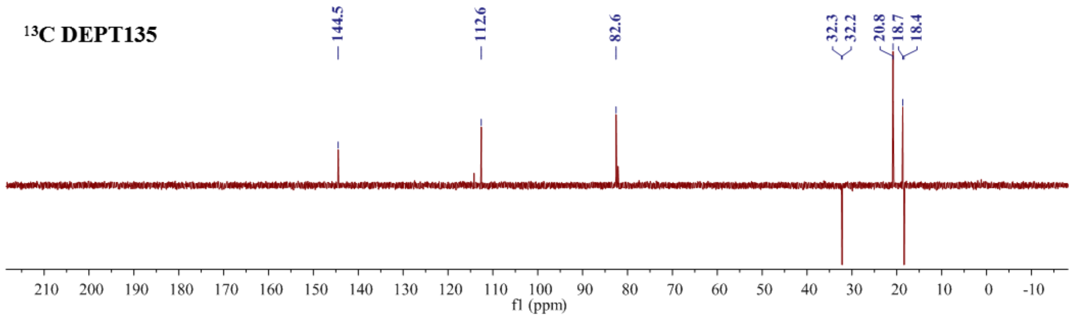

13C DEPT135
144.5
112.6
82.6
32.3
32.2
20.8
18.7
18.4
f1 (ppm)

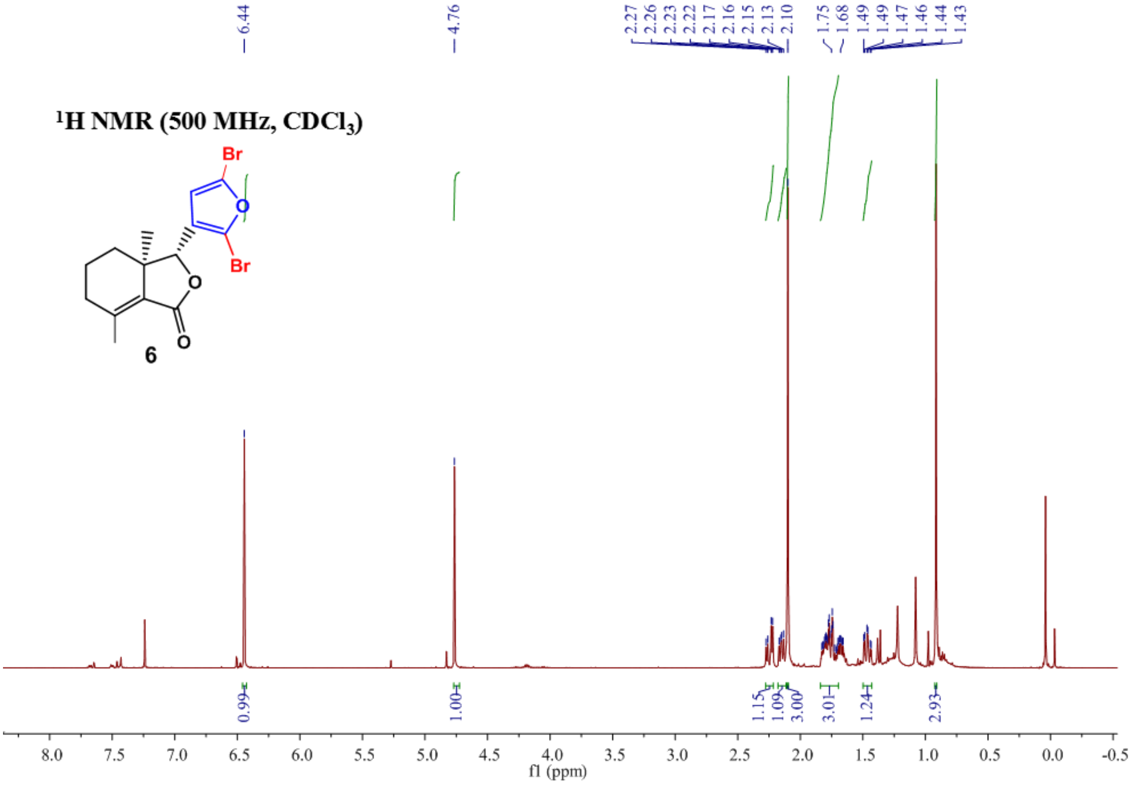

1H NMR (500 MHz, CDCl3)
6
6.44
4.76
2.27
2.26
2.23
2.22
2.17
2.16
2.15
2.13
2.10
1.75
1.68
1.49
1.49
1.47
1.46
1.44
1.43
0.99
1.00
1.15
1.09
3.00
3.01
1.24
2.93
f1 (ppm)

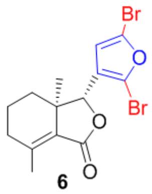

6

^{13}C NMR (126 MHz, CDCl$_3$)

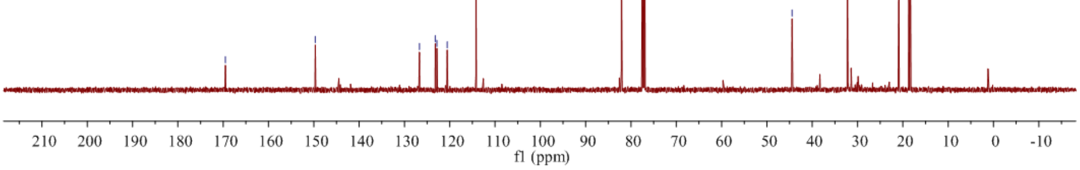

^{13}C DEPT135

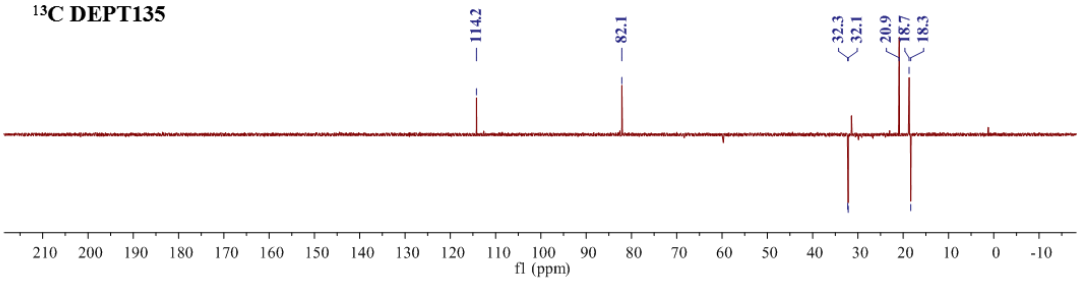

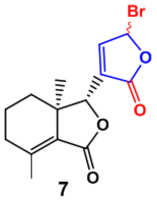

7

^{1}H NMR (500 MHz, CDCl$_3$)

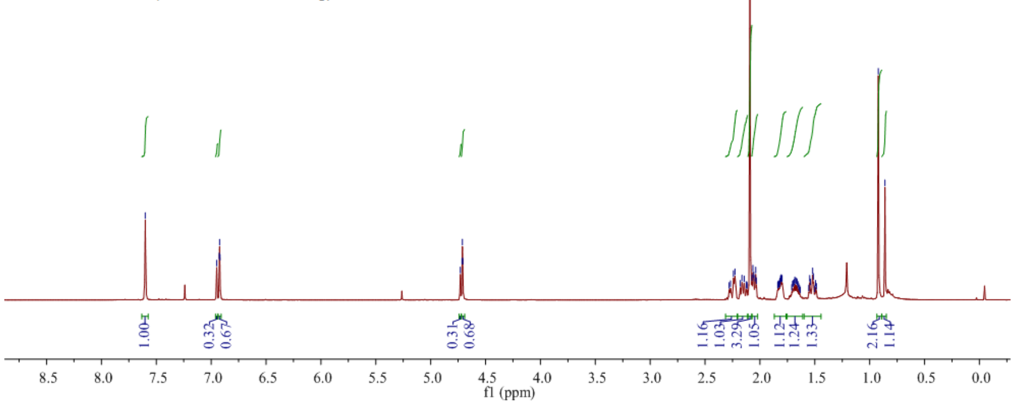

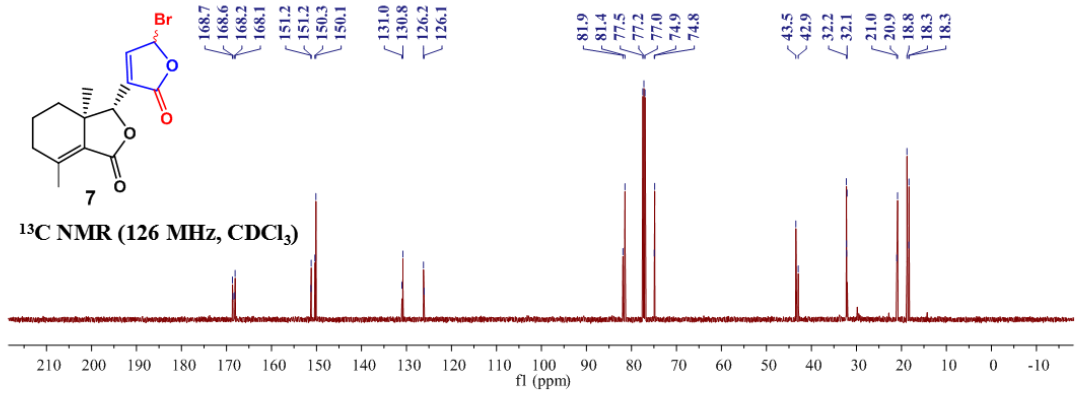
Br
O
O
O
O
7
13C NMR (126 MHz, CDCl3)
168.7
168.6
168.2
168.1
151.2
151.2
150.3
150.1
131.0
130.8
126.2
126.1
81.9
81.4
77.5
77.2
77.0
74.9
74.8
43.5
42.9
32.2
32.1
21.0
20.9
18.8
18.3
18.3
fl (ppm)

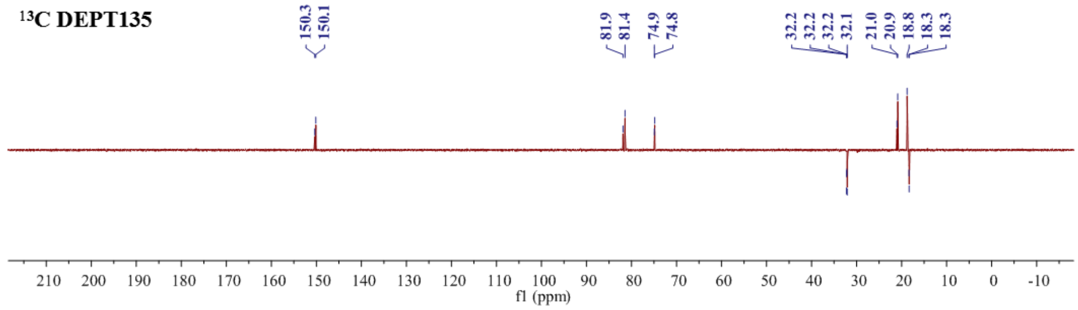
13C DEPT135
150.3
150.1
81.9
81.4
74.9
74.8
32.2
32.2
32.2
32.1
21.0
20.9
18.8
18.3
18.3
fl (ppm)

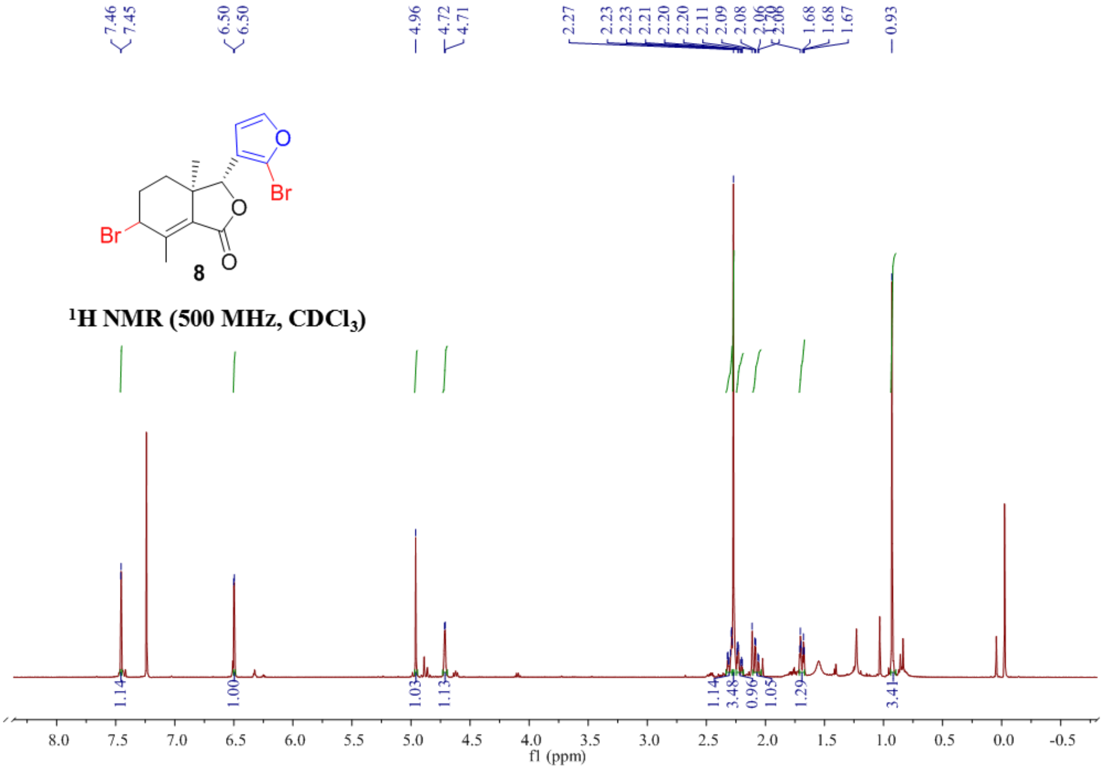
7.46
7.45
6.50
6.50
4.96
4.72
4.71
2.27
2.23
2.23
2.21
2.20
2.20
2.11
2.09
2.08
1.68
1.68
1.67
0.93
Br
O
Br
O
O
8
1H NMR (500 MHz, CDCl3)
1.14
1.00
1.03
1.13
1.14
3.48
0.96
1.05
1.29
3.41
fl (ppm)

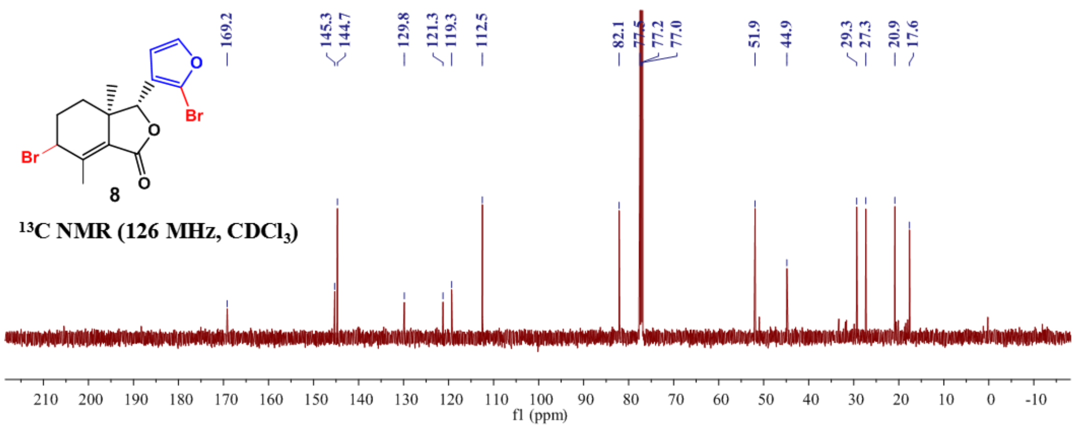

169.2
145.3
144.7
129.8
121.3
119.3
112.5
82.1
77.5
77.2
77.0
51.9
44.9
29.3
27.3
20.9
17.6
Br
O
Br
O
O
8
¹³C NMR (126 MHz, CDCl₃)
fl (ppm)

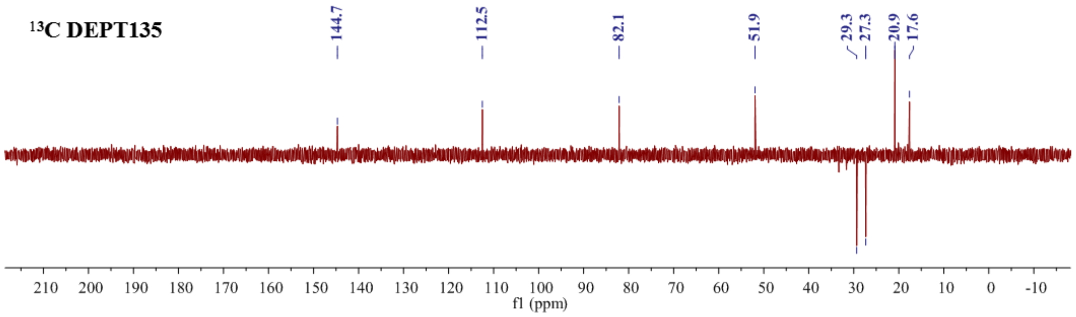

¹³C DEPT135
144.7
112.5
82.1
51.9
29.3
27.3
20.9
17.6
fl (ppm)

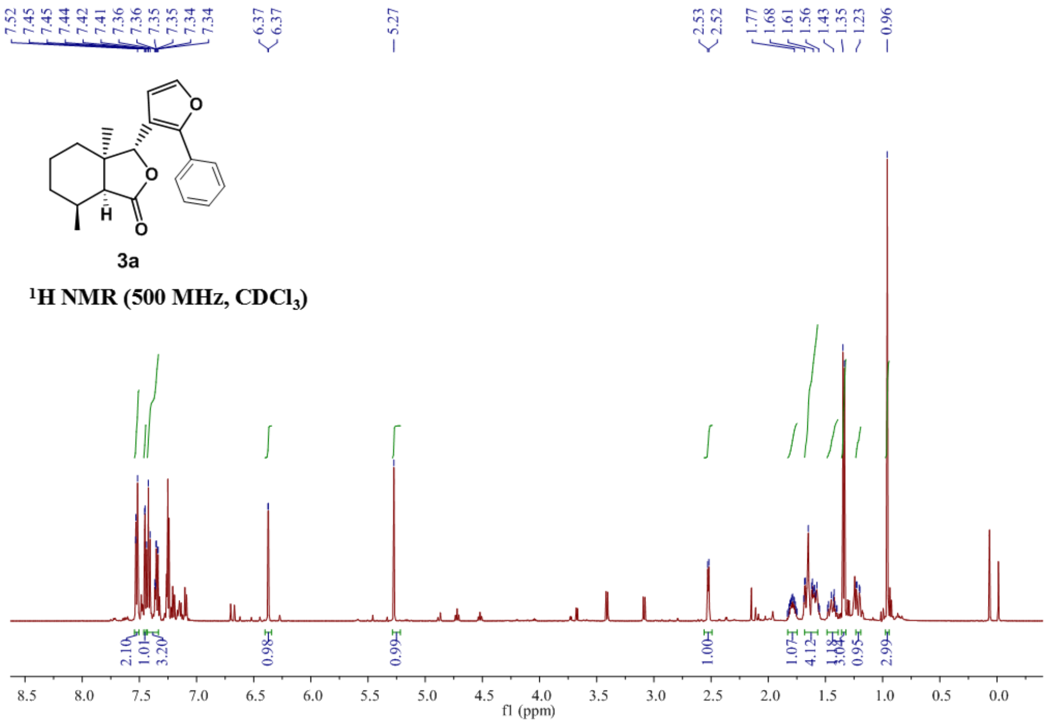

7.52
7.45
7.45
7.44
7.42
7.41
7.36
7.36
7.35
7.35
7.34
7.34
6.37
6.37
5.27
2.53
2.52
1.77
1.68
1.61
1.56
1.43
1.35
1.23
0.96
O
O
H
O
3a
¹H NMR (500 MHz, CDCl₃)
2.10
1.01
3.20
0.98
0.99
1.00
1.07
4.12
1.18
3.04
0.95
2.99
fl (ppm)

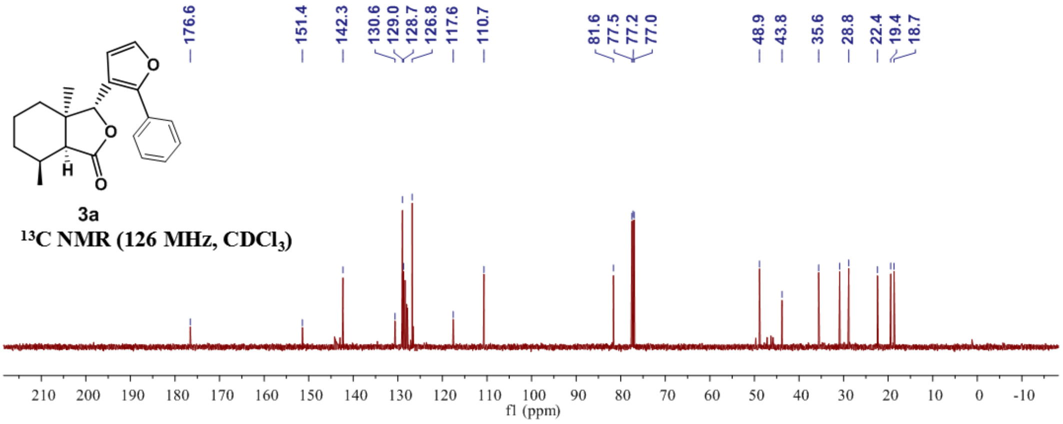
176.6
151.4
142.3
130.6
129.0
128.7
126.8
117.6
110.7
81.6
77.5
77.2
77.0
48.9
43.8
35.6
28.8
22.4
19.4
18.7
3a
13C NMR (126 MHz, CDCl3)
f1 (ppm)

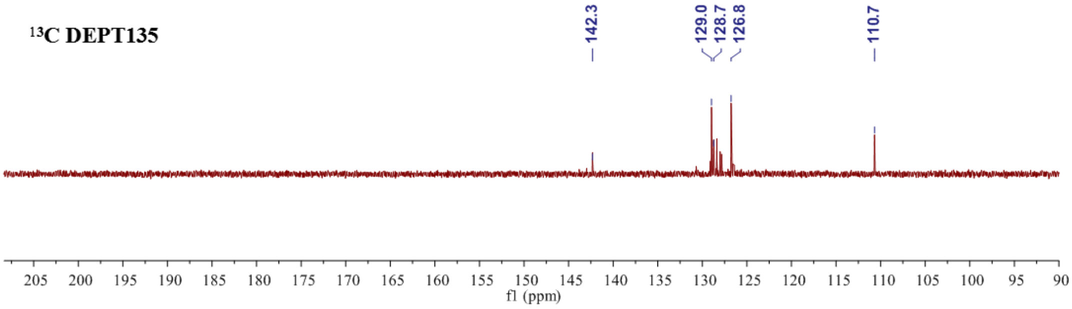
13C DEPT135
142.3
129.0
128.7
126.8
110.7
f1 (ppm)

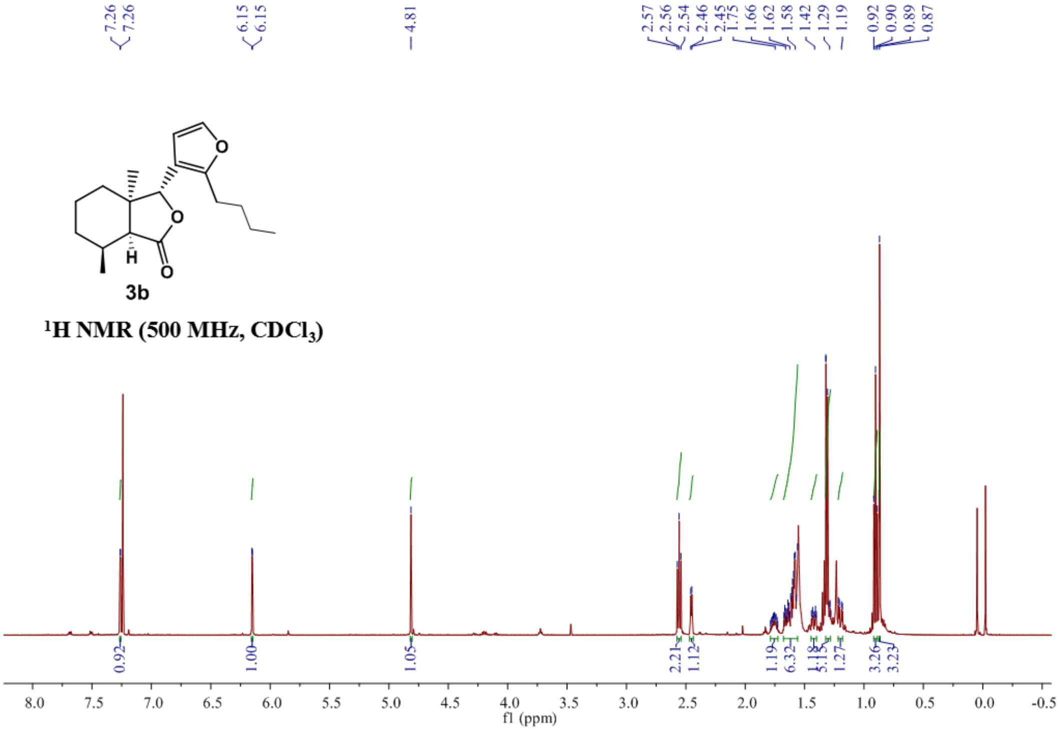
7.26
7.26
6.15
6.15
4.81
2.57
2.56
2.54
2.46
2.45
1.75
1.66
1.62
1.58
1.42
1.29
1.19
0.92
0.90
0.89
0.87
3b
1H NMR (500 MHz, CDCl3)
0.92
1.00
1.05
2.21
1.12
1.19
6.32
1.18
5.15
1.27
3.26
3.23
f1 (ppm)

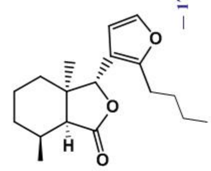

3b

^{13}C NMR (126 MHz, $CDCl_3$)

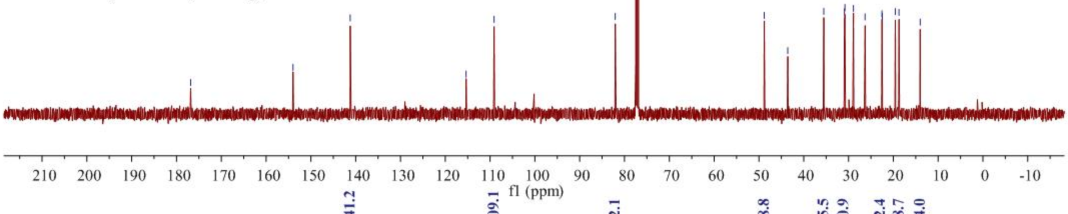

^{13}C DEPT135

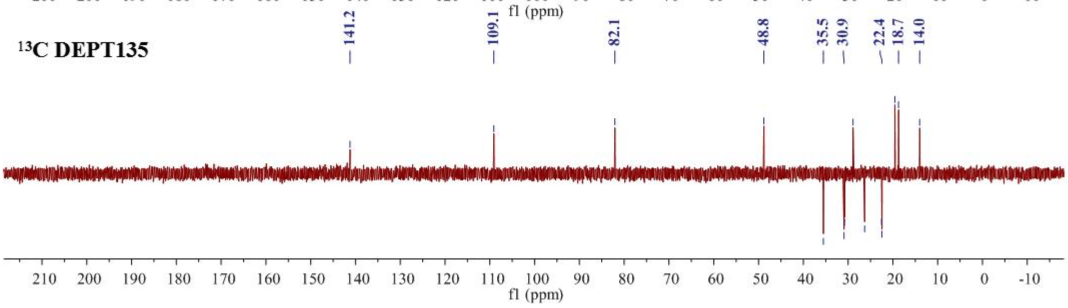

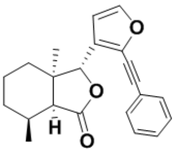

3c

^{1}H NMR (500 MHz, $CDCl_3$)

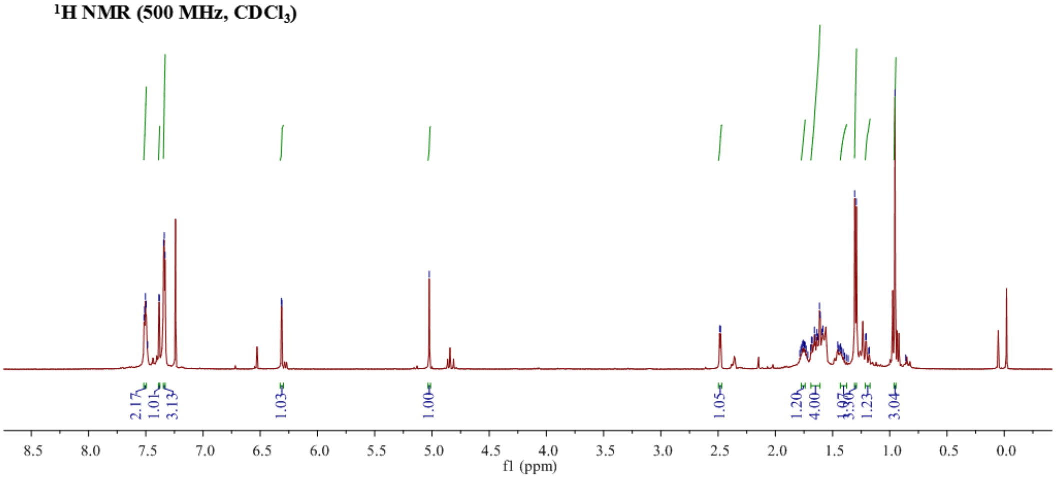

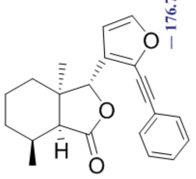

3c

^{13}C NMR (126 MHz, $CDCl_3$)

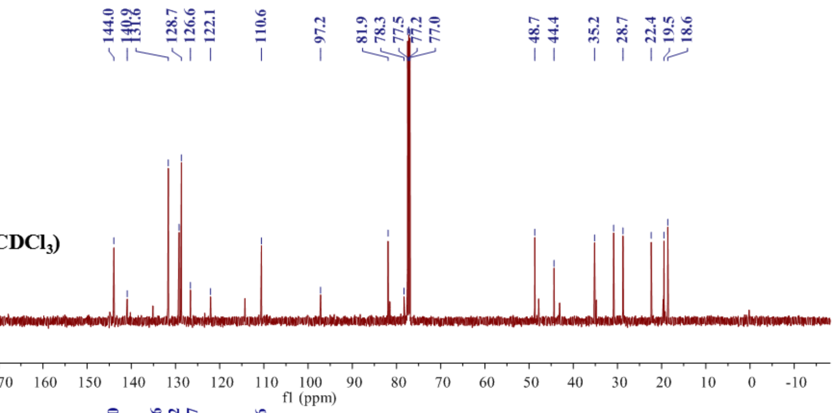

^{13}C DEPT135

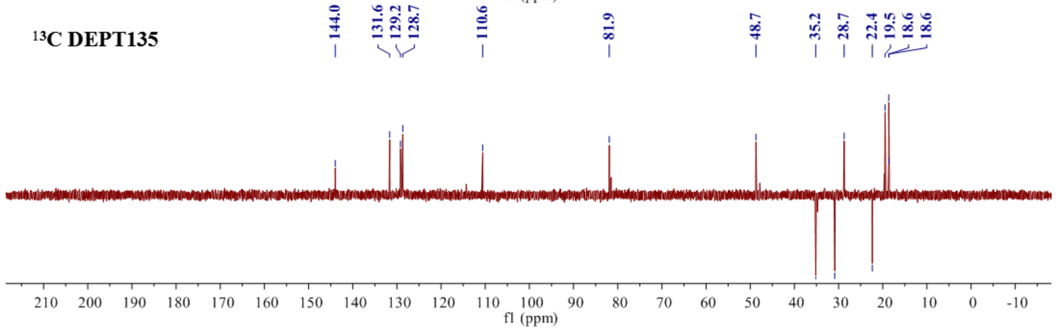

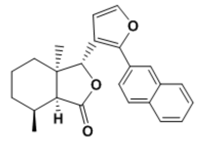

3d

^{1}H NMR (500 MHz, $CDCl_3$)

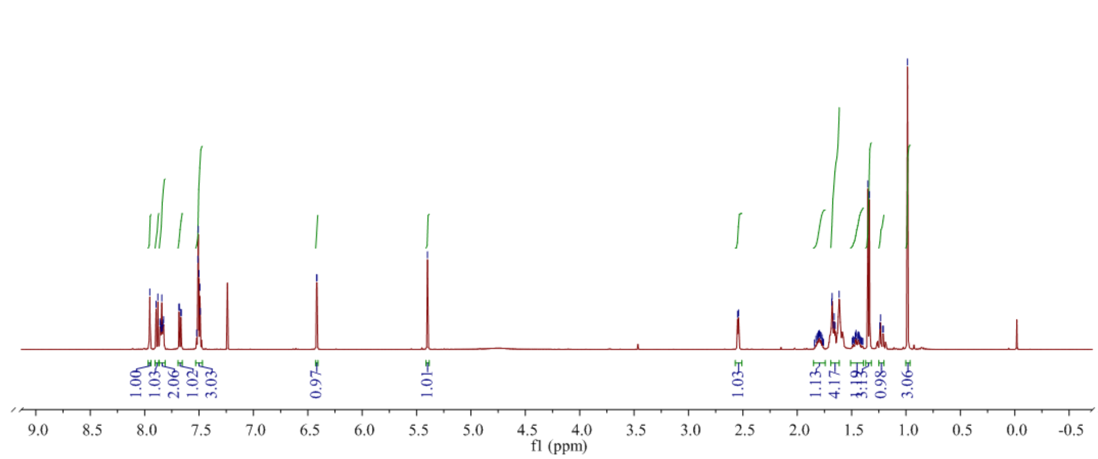

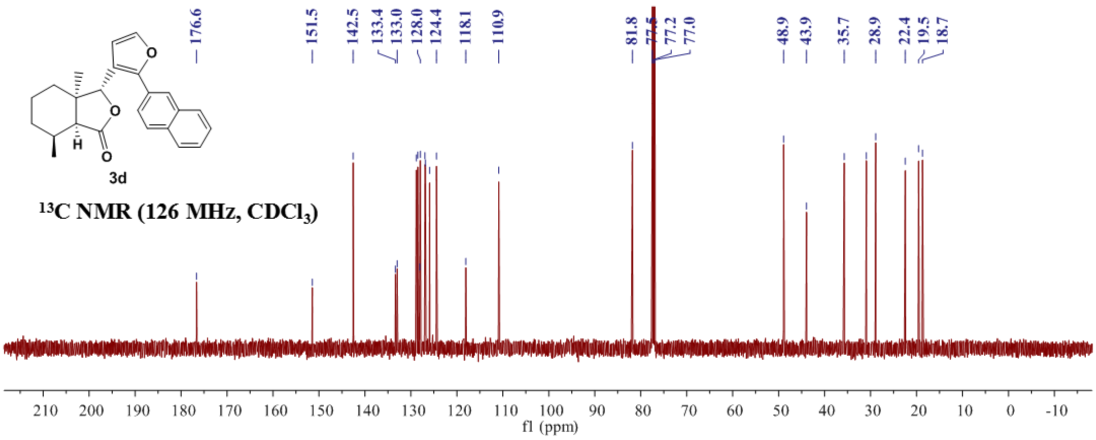
176.6
151.5
142.5
133.4
133.0
128.0
124.4
118.1
110.9
81.8
77.5
77.2
77.0
48.9
43.9
35.7
28.9
22.4
19.5
18.7
3d
^{13}C NMR (126 MHz, $CDCl_3$)
f1 (ppm)

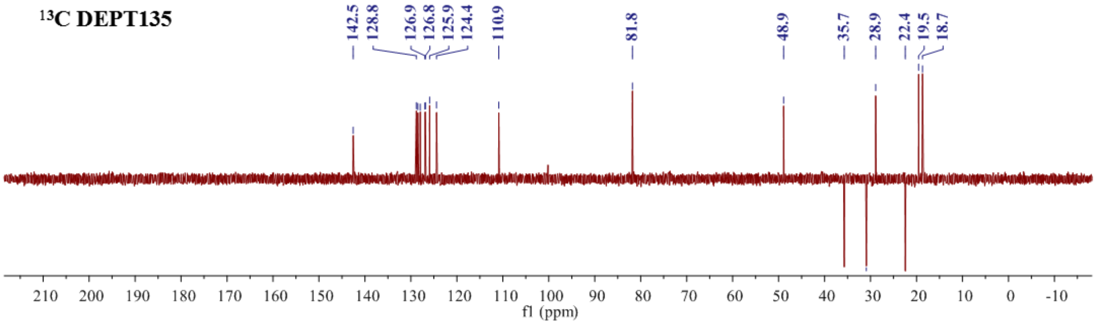
^{13}C DEPT135
142.5
128.8
126.9
126.8
125.9
124.4
110.9
81.8
48.9
35.7
28.9
22.4
19.5
18.7
f1 (ppm)

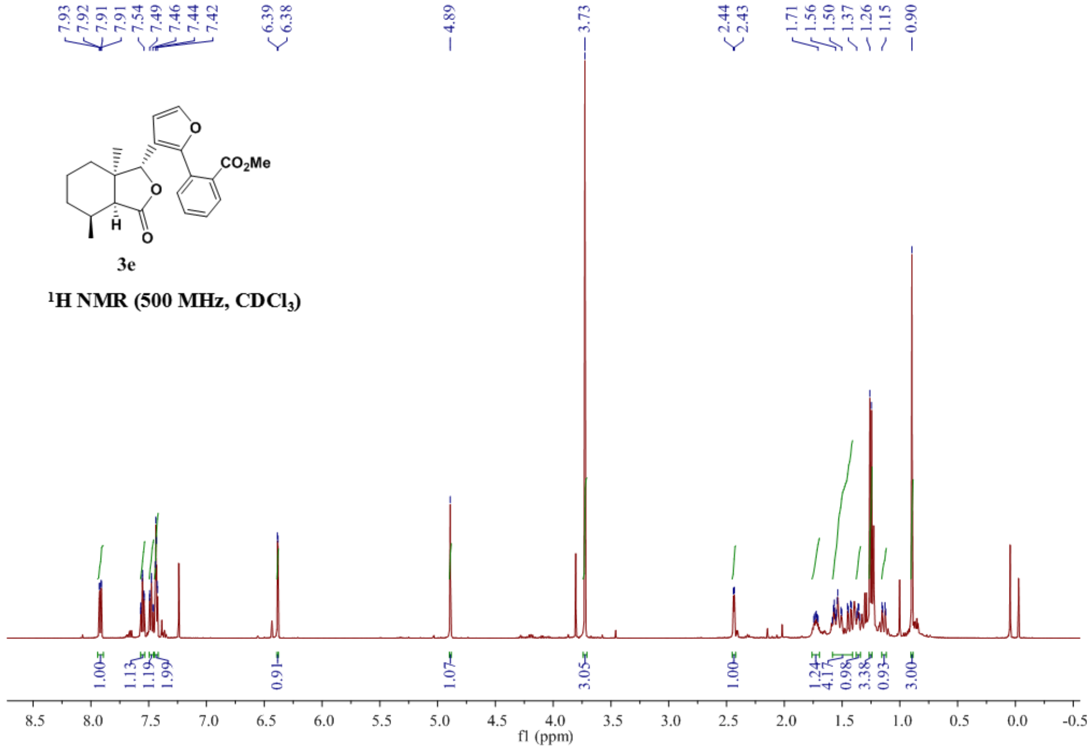
7.93
7.92
7.91
7.91
7.54
7.49
7.46
7.44
7.42
6.39
6.38
4.89
3.73
2.44
2.43
1.71
1.56
1.50
1.37
1.26
1.15
0.90
CO_2Me
3e
^1H NMR (500 MHz, $CDCl_3$)
1.00
1.13
1.19
1.99
0.91
1.07
3.05
1.00
1.24
4.17
0.98
3.38
0.93
3.00
f1 (ppm)

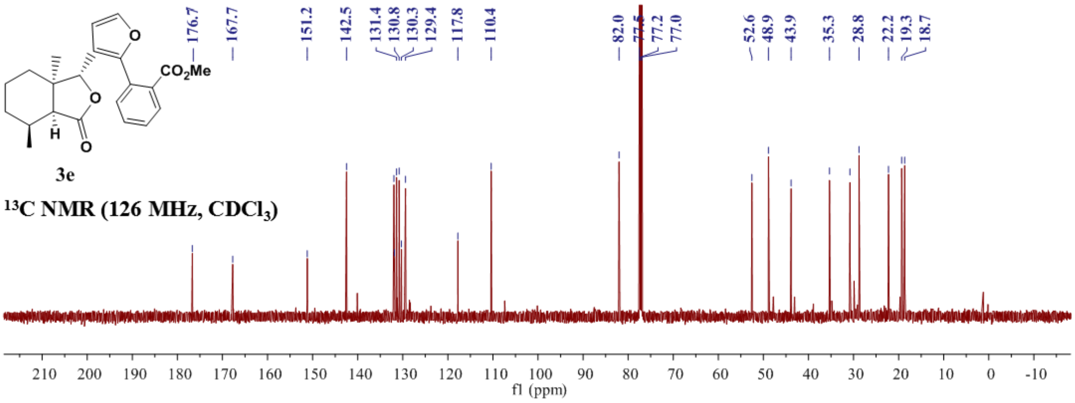
CO2Me
3e
13C NMR (126 MHz, CDCl3)
176.7
167.7
151.2
142.5
131.4
130.8
130.3
129.4
117.8
110.4
82.0
77.5
77.2
77.0
52.6
48.9
43.9
35.3
28.8
22.2
19.3
18.7
f1 (ppm)

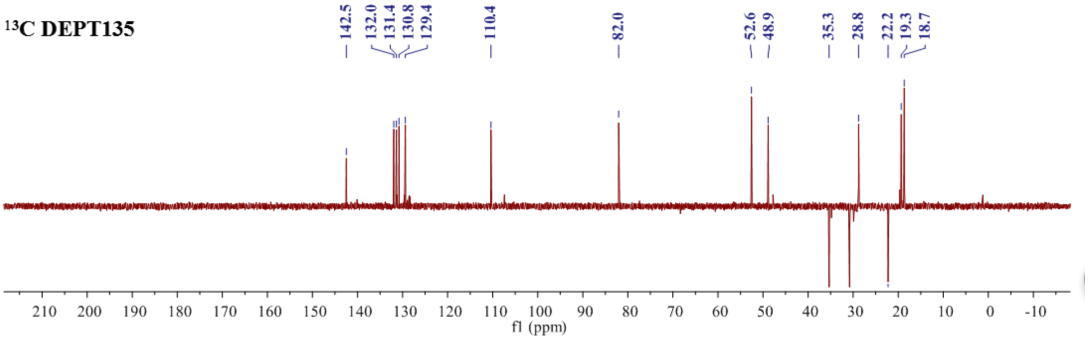
13C DEPT135
142.5
132.0
131.4
130.8
129.4
110.4
82.0
52.6
48.9
35.3
28.8
22.2
19.3
18.7
f1 (ppm)

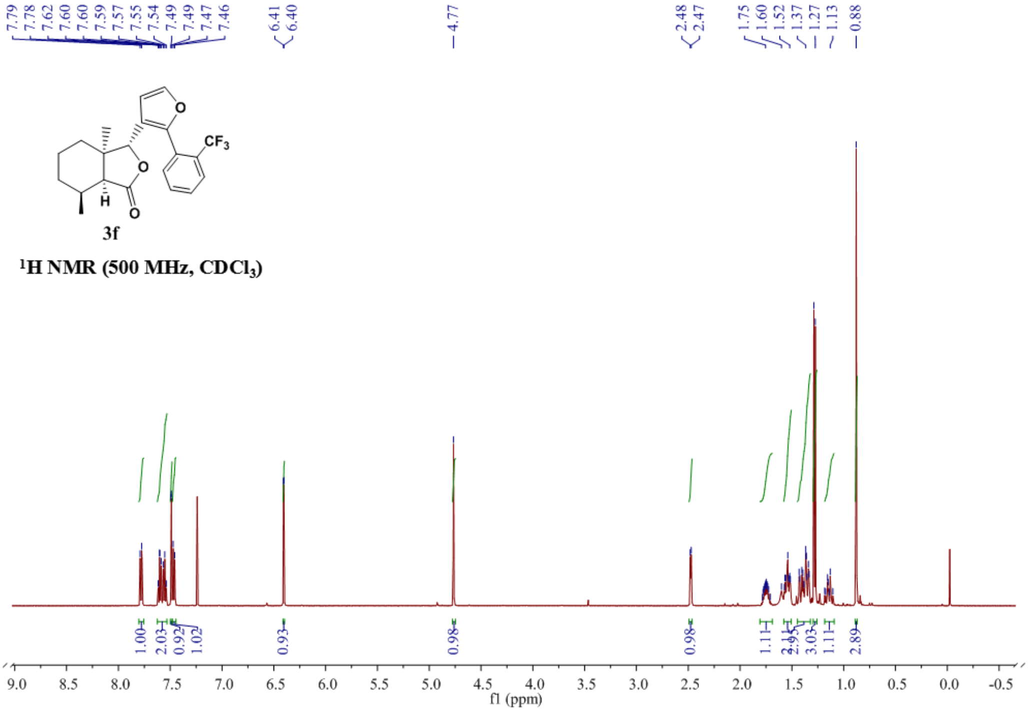
7.79
7.78
7.62
7.60
7.60
7.59
7.57
7.55
7.54
7.49
7.49
7.47
7.46
6.41
6.40
4.77
2.48
2.47
1.75
1.60
1.52
1.37
1.27
1.13
0.88
CF3
3f
1H NMR (500 MHz, CDCl3)
1.00
2.03
0.92
1.02
0.93
0.98
0.98
1.11
2.11
2.95
3.03
1.11
2.89
f1 (ppm)

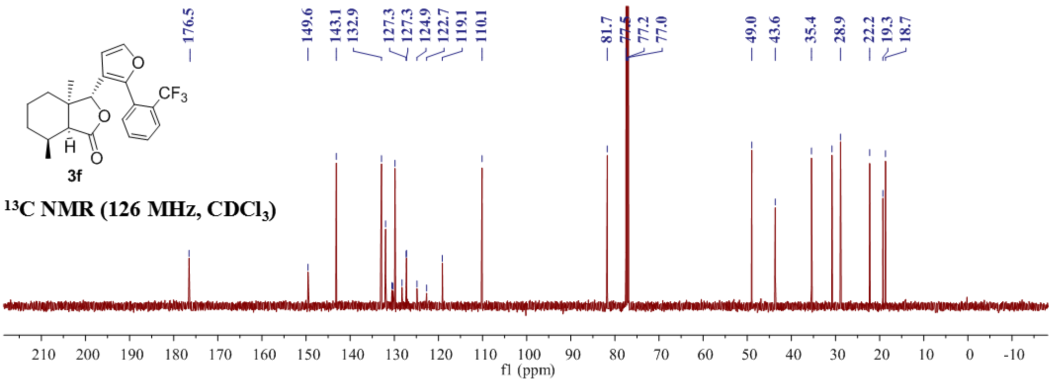
176.5
149.6
143.1
132.9
127.3
127.3
124.9
122.7
119.1
110.1
81.7
77.5
77.2
77.0
49.0
43.6
35.4
28.9
22.2
19.3
18.7
CF3
O
H
3f
13C NMR (126 MHz, CDCl3)
f1 (ppm)

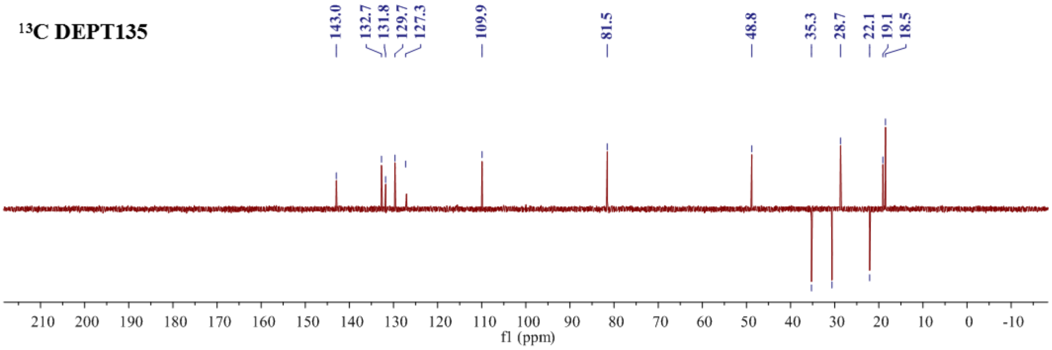
13C DEPT135
143.0
132.7
131.8
129.7
127.3
109.9
81.5
48.8
35.3
28.7
22.1
19.1
18.5
f1 (ppm)

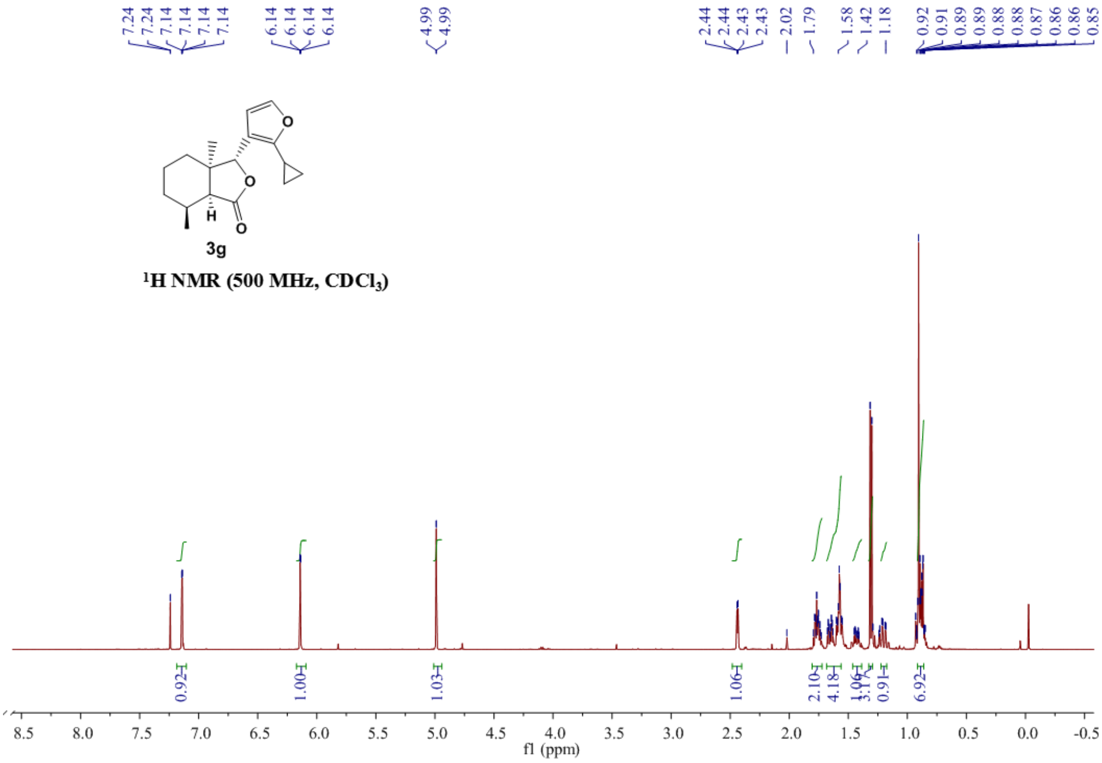
7.24
7.24
7.14
7.14
7.14
7.14
6.14
6.14
6.14
6.14
4.99
4.99
2.44
2.44
2.43
2.43
2.02
1.79
1.58
1.42
1.18
0.92
0.91
0.89
0.89
0.88
0.88
0.87
0.86
0.86
0.85
O
H
3g
1H NMR (500 MHz, CDCl3)
0.92
1.00
1.03
1.06
2.10
4.18
1.06
3.17
0.91
6.92
f1 (ppm)

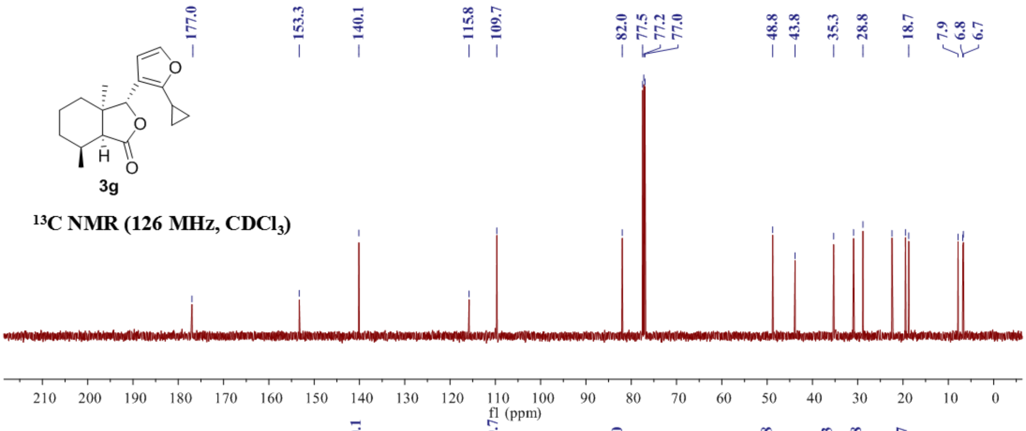
177.0
153.3
140.1
115.8
109.7
82.0
77.5
77.2
77.0
48.8
43.8
35.3
28.8
18.7
7.9
6.8
6.7
O
O
H
O
3g
13C NMR (126 MHz, CDCl3)
210 200 190 180 170 160 150 140 130 120 110 100 90 80 70 60 50 40 30 20 10 0
f1 (ppm)

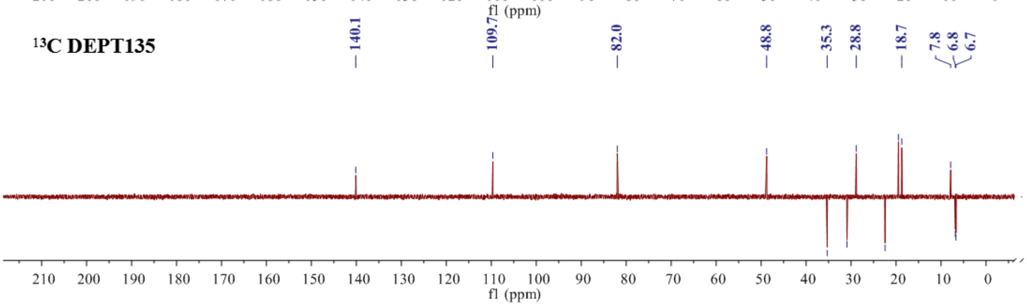
13C DEPT135
140.1
109.7
82.0
48.8
35.3
28.8
18.7
7.8
6.8
6.7
210 200 190 180 170 160 150 140 130 120 110 100 90 80 70 60 50 40 30 20 10 0
f1 (ppm)

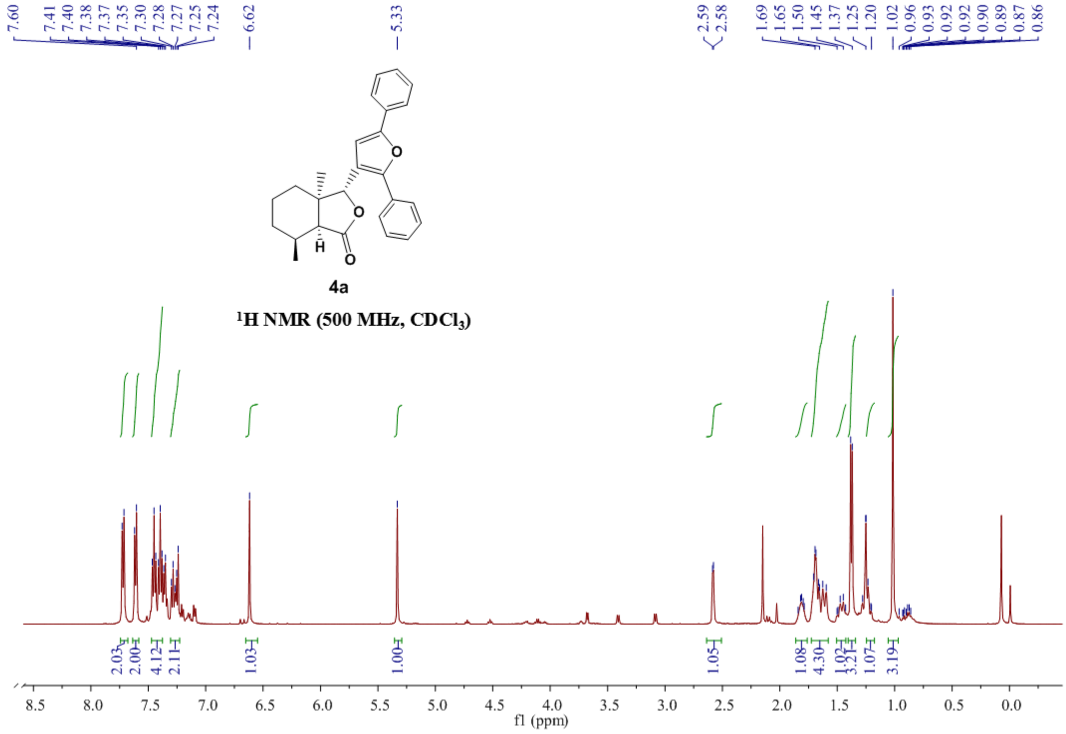
7.60
7.41
7.40
7.38
7.37
7.35
7.30
7.28
7.27
7.25
7.24
6.62
5.33
2.59
2.58
1.69
1.65
1.50
1.45
1.37
1.25
1.20
1.02
0.96
0.93
0.92
0.92
0.90
0.89
0.87
0.86
O
O
H
O
4a
1H NMR (500 MHz, CDCl3)
2.03
2.00
4.12
2.11
1.03
1.00
1.05
1.08
4.30
1.02
3.21
1.07
3.19
8.5 8.0 7.5 7.0 6.5 6.0 5.5 5.0 4.5 4.0 3.5 3.0 2.5 2.0 1.5 1.0 0.5 0.0
f1 (ppm)

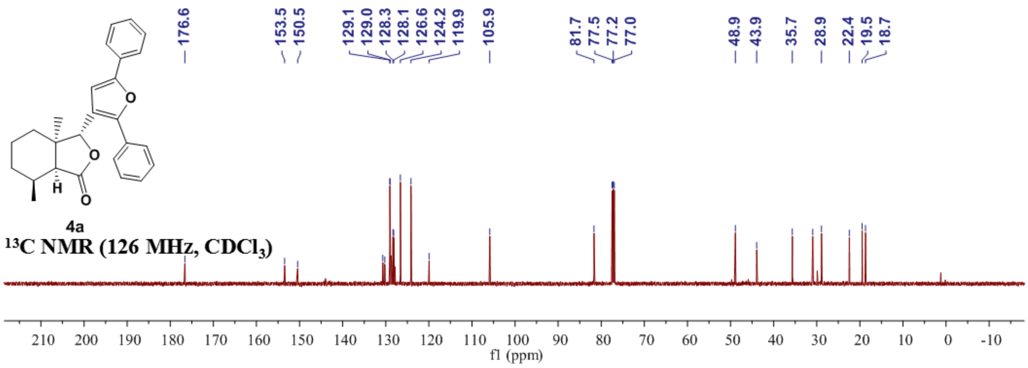
176.6
153.5
150.5
129.1
129.0
128.3
128.1
126.6
124.2
119.9
105.9
81.7
77.5
77.2
77.0
48.9
43.9
35.7
28.9
22.4
19.5
18.7
4a
^{13}C NMR (126 MHz, $CDCl_3$)
f1 (ppm)

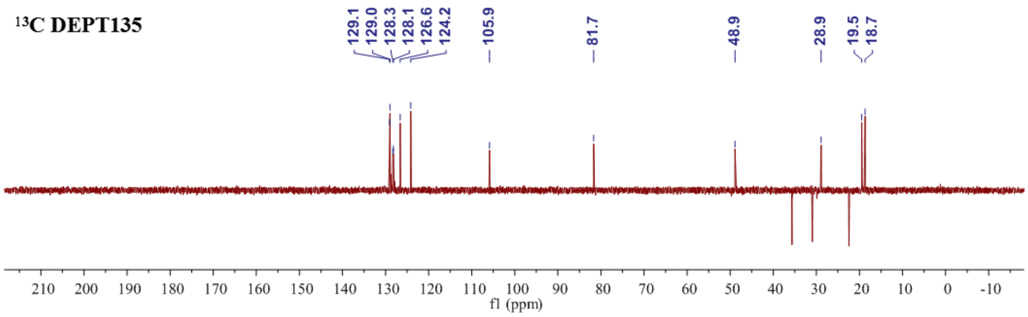
^{13}C DEPT135
129.1
129.0
128.3
128.1
126.6
124.2
105.9
81.7
48.9
28.9
19.5
18.7
f1 (ppm)

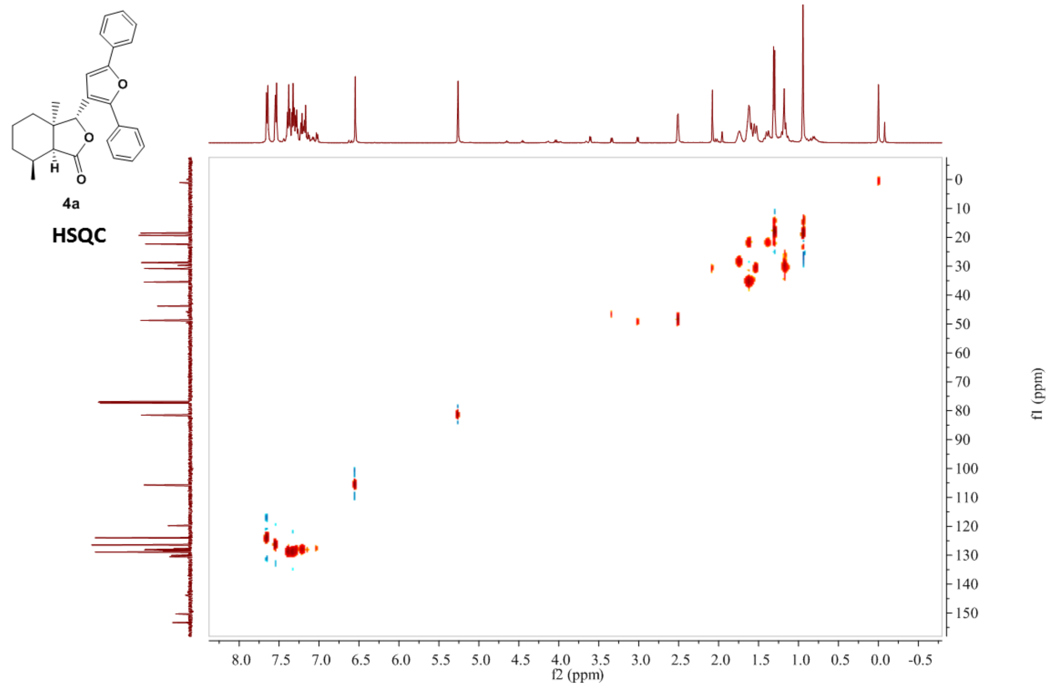
4a
HSQC
f2 (ppm)
f1 (ppm)

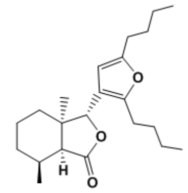

4b

^{1}H NMR (500 MHz, CDCl$_3$)

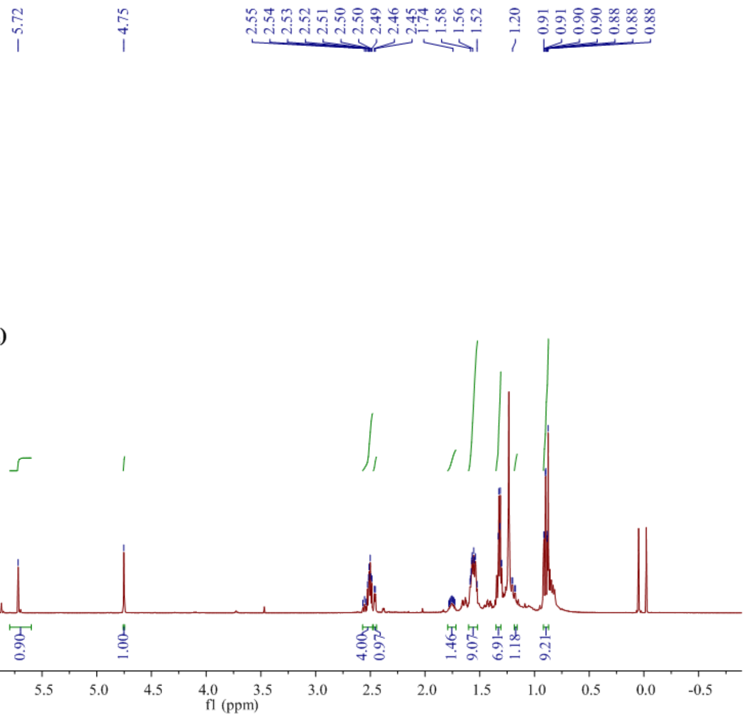

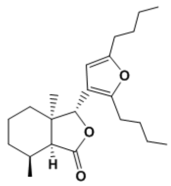

4b

^{13}C NMR (126 MHz, CDCl$_3$)

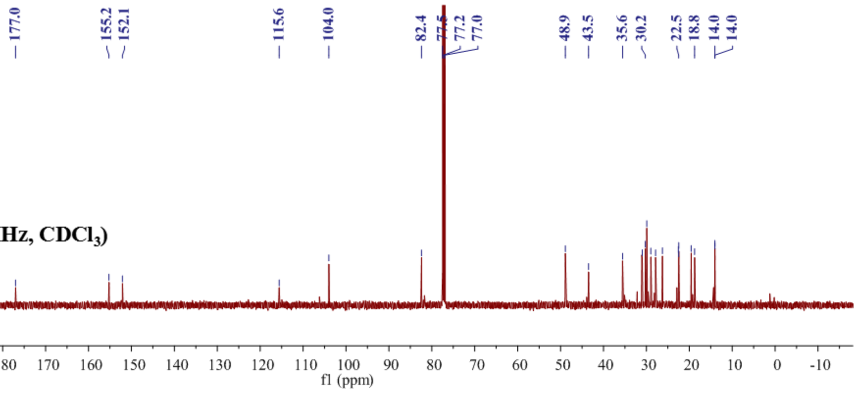

^{13}C DEPT135

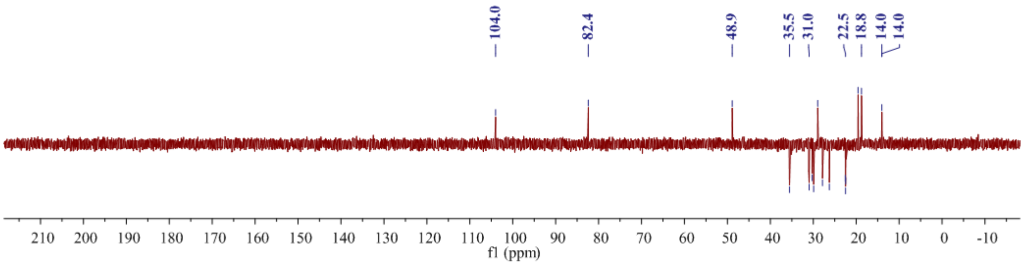

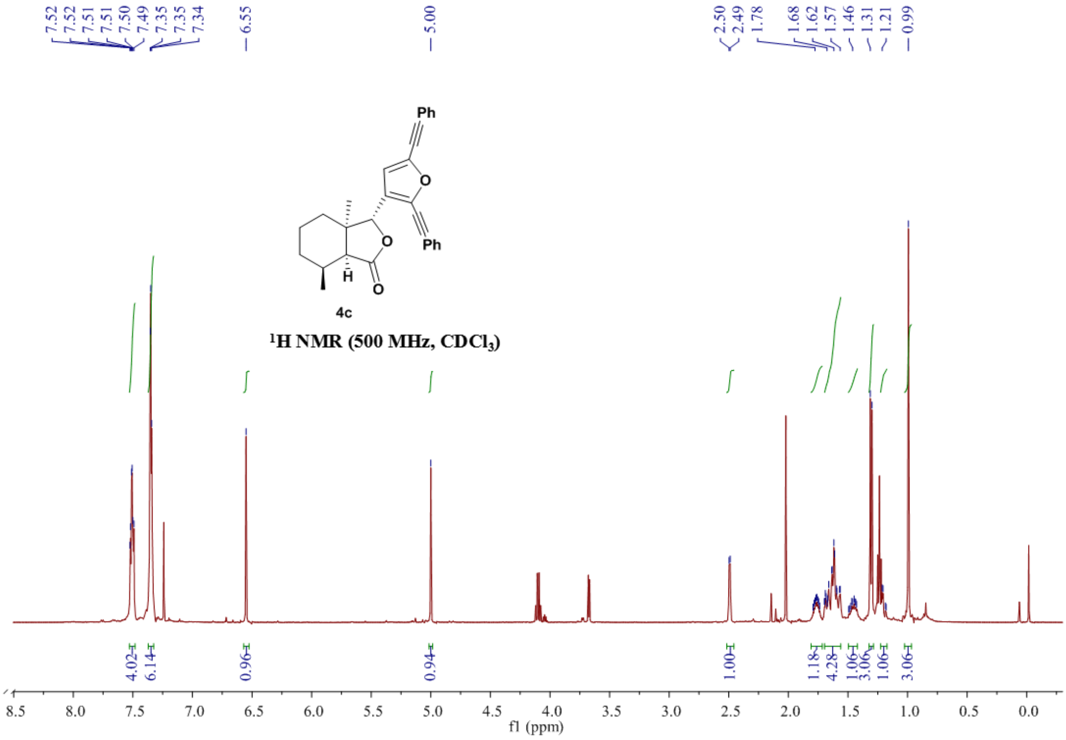

7.52 7.52 7.51 7.51 7.50 7.49 7.35 7.35 7.34
6.55
5.00
2.50 2.49 1.78 1.68 1.62 1.57 1.46 1.31 1.21 0.99
Ph
O
Ph
O
H
O
4c
¹H NMR (500 MHz, CDCl₃)
4.02 6.14 0.96 0.94 1.00 1.18 4.28 1.06 3.06 1.06 3.06
8.5 8.0 7.5 7.0 6.5 6.0 5.5 5.0 4.5 4.0 3.5 3.0 2.5 2.0 1.5 1.0 0.5 0.0
f1 (ppm)

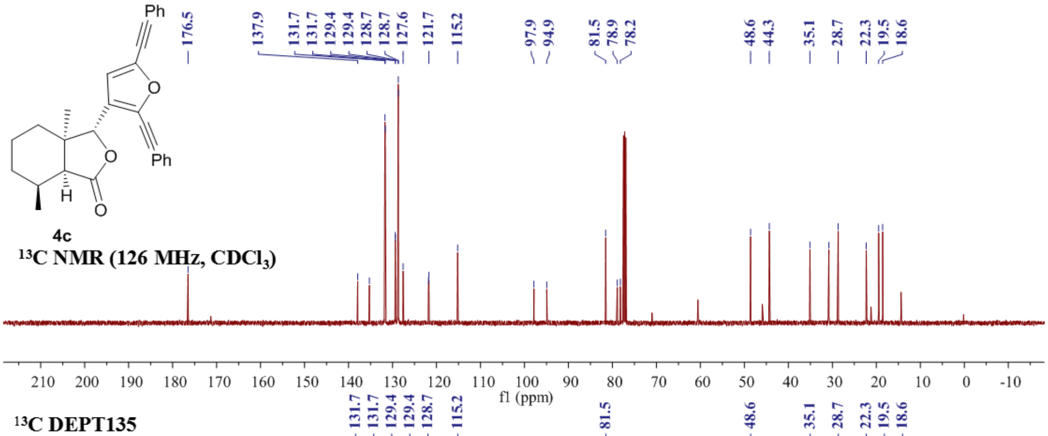

176.5
137.9 131.7 131.7 129.4 129.4 128.7 128.7 127.6 121.7 115.2
97.9 94.9 81.5 78.9 78.2
48.6 44.3 35.1 28.7 22.3 19.5 18.6
Ph
O
Ph
O
H
O
4c
¹³C NMR (126 MHz, CDCl₃)
210 200 190 180 170 160 150 140 130 120 110 100 90 80 70 60 50 40 30 20 10 0 -10
f1 (ppm)

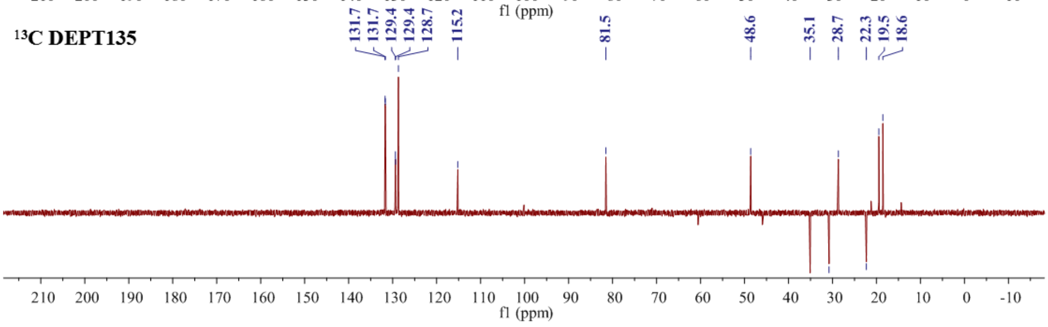

¹³C DEPT135
131.7 131.7 129.4 129.4 128.7 115.2
81.5
48.6 35.1 28.7 22.3 19.5 18.6
210 200 190 180 170 160 150 140 130 120 110 100 90 80 70 60 50 40 30 20 10 0 -10
f1 (ppm)

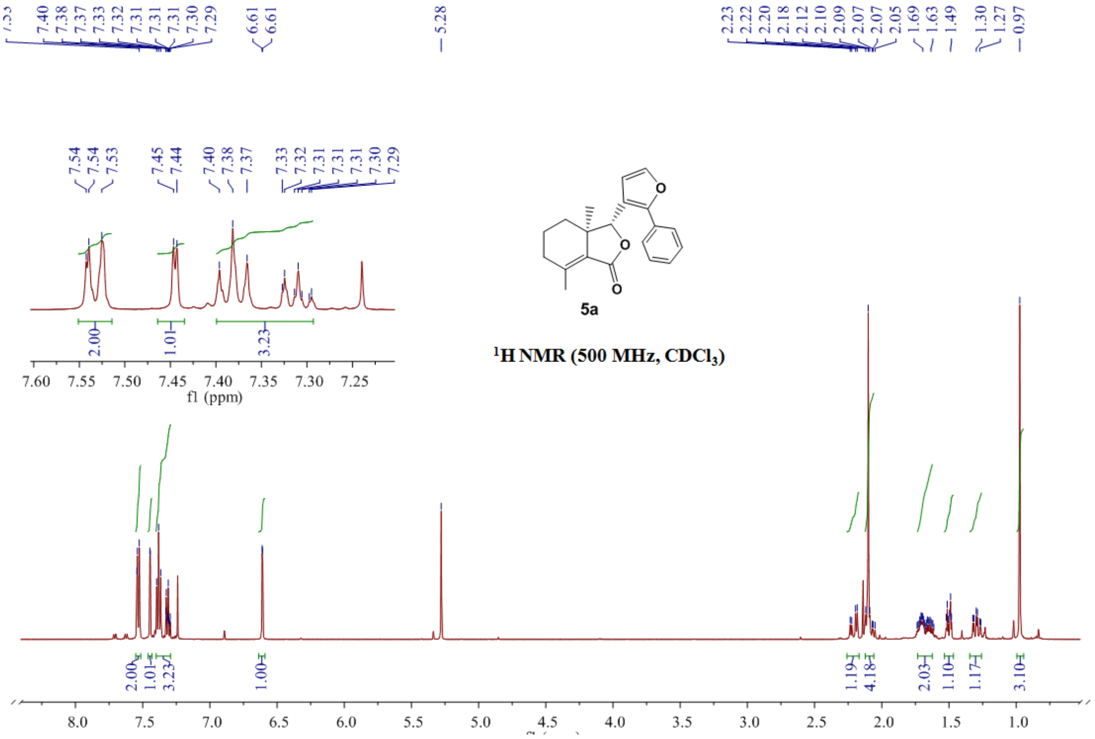

5a
1H NMR (500 MHz, CDCl3)
5.28
6.61
2.00
1.01
3.23
1.00
1.19
4.18
2.03
1.10
1.17
3.10
fl (ppm)

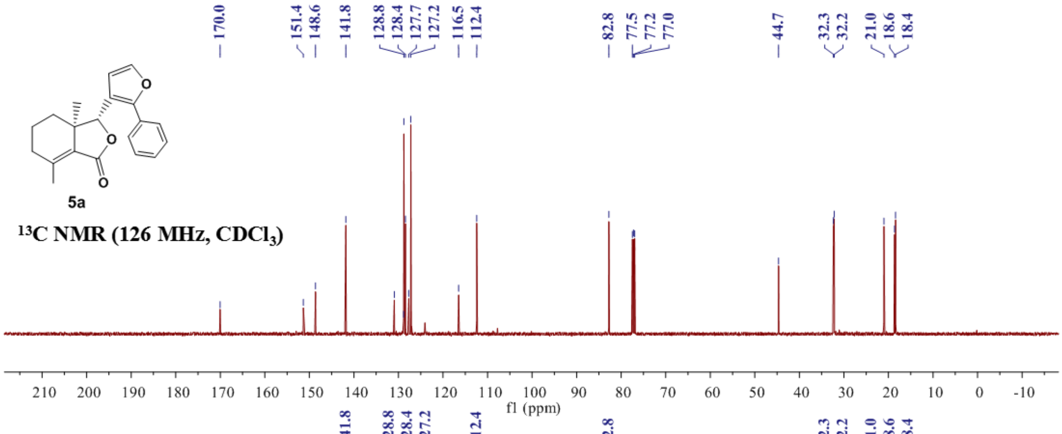

170.0
151.4
148.6
141.8
128.8
128.4
127.7
127.2
116.5
112.4
82.8
77.5
77.2
77.0
44.7
32.3
32.2
21.0
18.6
18.4
5a
13C NMR (126 MHz, CDCl3)
fl (ppm)

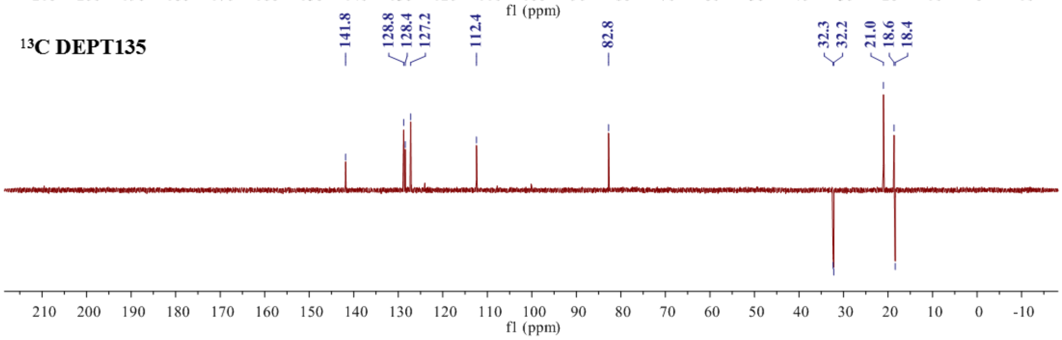

13C DEPT135
141.8
128.8
128.4
127.2
112.4
82.8
32.3
32.2
21.0
18.6
18.4
fl (ppm)

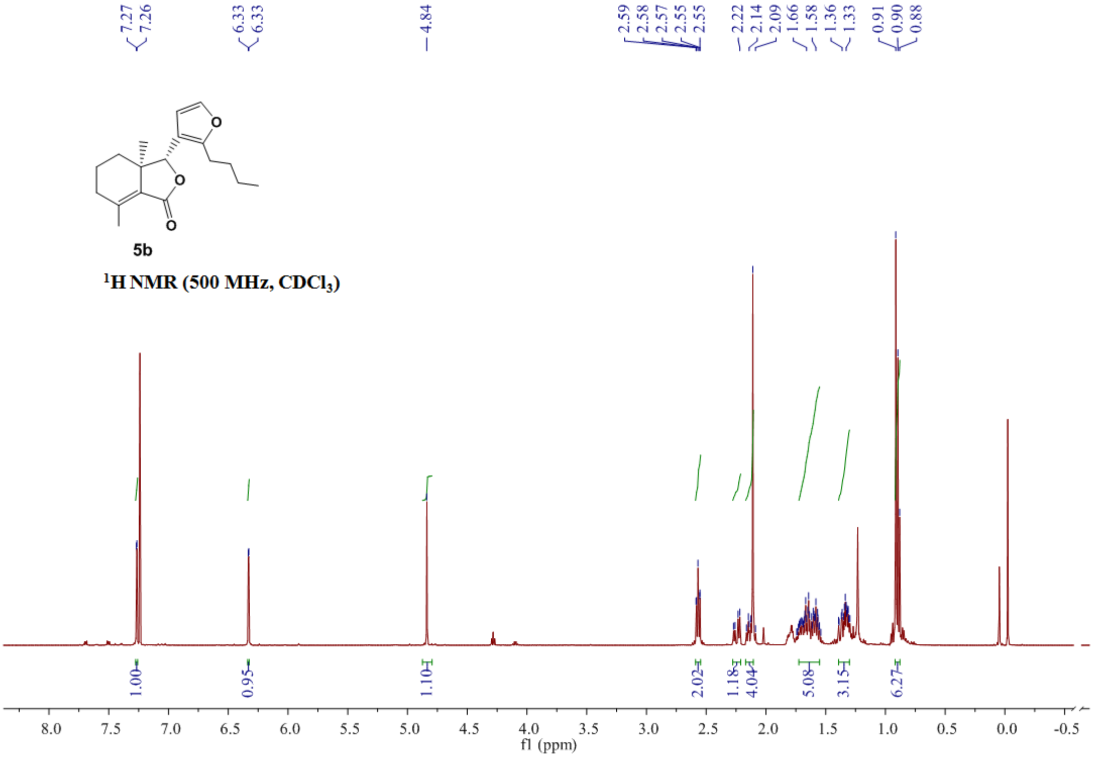

7.27
7.26
6.33
6.33
4.84
2.59
2.58
2.57
2.55
2.55
2.22
2.14
2.09
1.66
1.58
1.36
1.33
0.91
0.90
0.88
O
O
O
5b
1H NMR (500 MHz, CDCl3)
1.00
0.95
1.10
2.02
1.18
4.04
5.08
3.15
6.27
f1 (ppm)

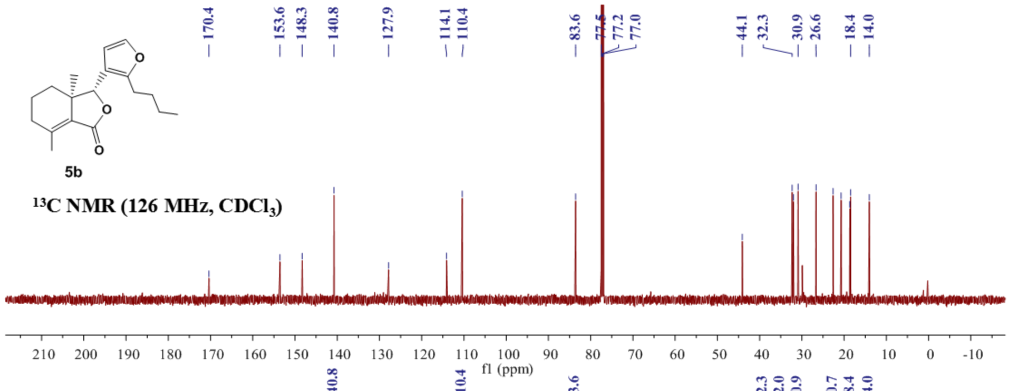

170.4
153.6
148.3
140.8
127.9
114.1
110.4
83.6
77.5
77.2
77.0
44.1
32.3
30.9
26.6
18.4
14.0
5b
13C NMR (126 MHz, CDCl3)
f1 (ppm)

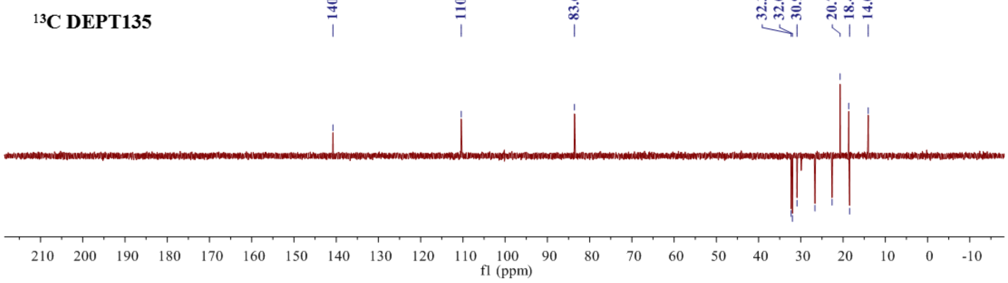

13C DEPT135
140.8
110.4
83.6
32.3
32.0
30.9
20.7
18.4
14.0
f1 (ppm)

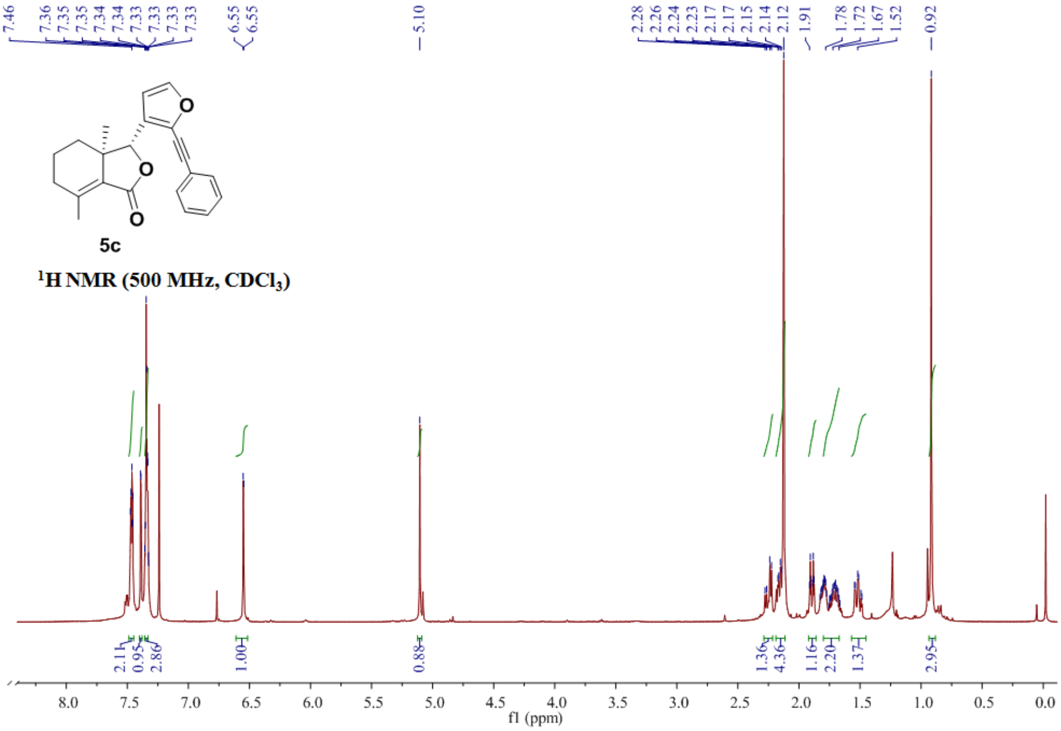
5c
¹H NMR (500 MHz, CDCl₃)
fl (ppm)

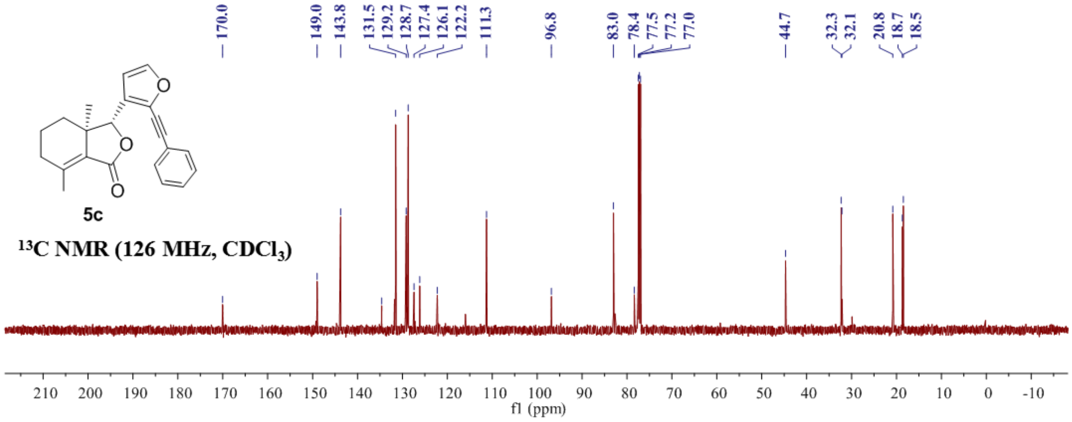
5c
¹³C NMR (126 MHz, CDCl₃)
fl (ppm)

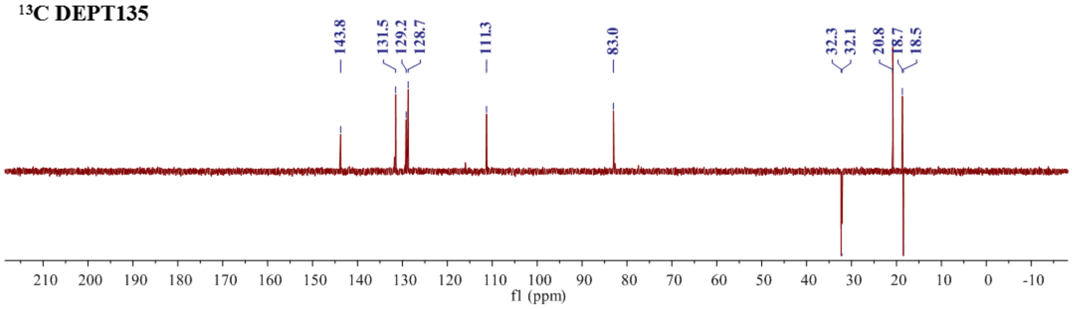
¹³C DEPT135
fl (ppm)

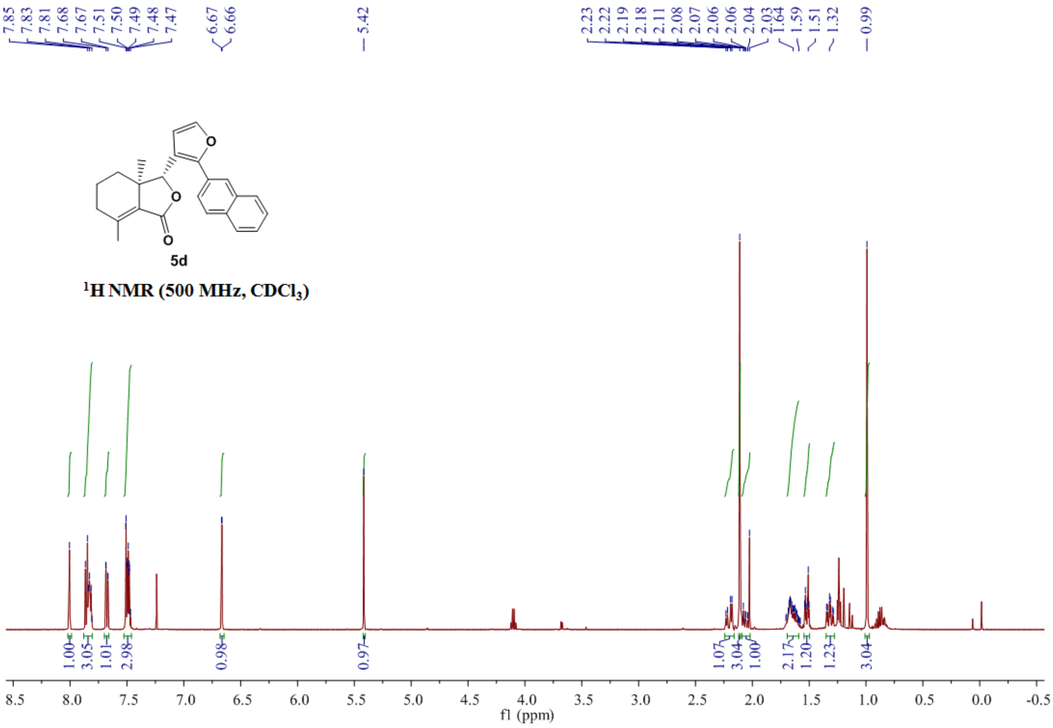
7.85 7.83 7.81 7.68 7.67 7.51 7.50 7.49 7.48 7.47
6.67 6.66
5.42
2.23 2.22 2.19 2.18 2.11 2.08 2.07 2.06 2.06 2.04 2.03 1.64 1.59 1.51 1.32
0.99
5d
^{1}H NMR (500 MHz, CDCl$_3$)
1.00 3.05 1.01 2.98 0.98 0.97 1.07 3.04 1.00 2.17 1.20 1.23 3.04
fl (ppm)

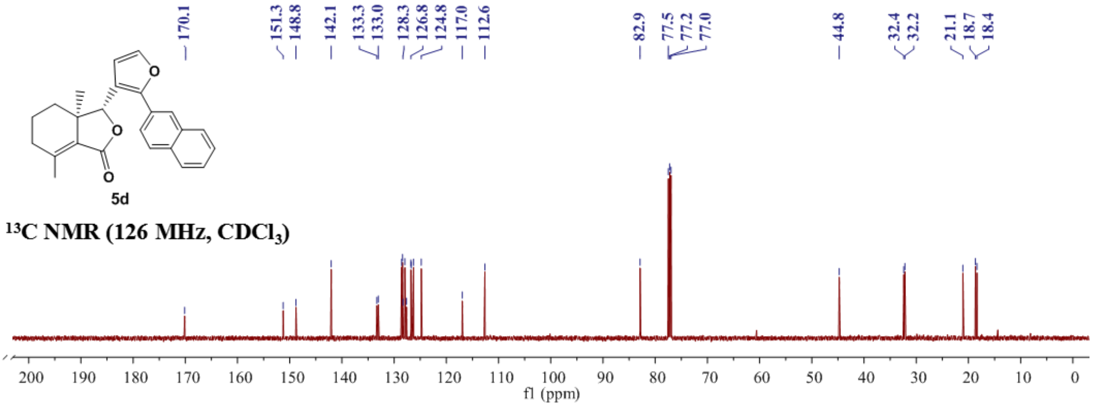
170.1
151.3 148.8
142.1
133.3 133.0
128.3 126.8 124.8
117.0
112.6
82.9
77.5 77.2 77.0
44.8
32.4 32.2
21.1 18.7 18.4
5d
^{13}C NMR (126 MHz, CDCl$_3$)
fl (ppm)

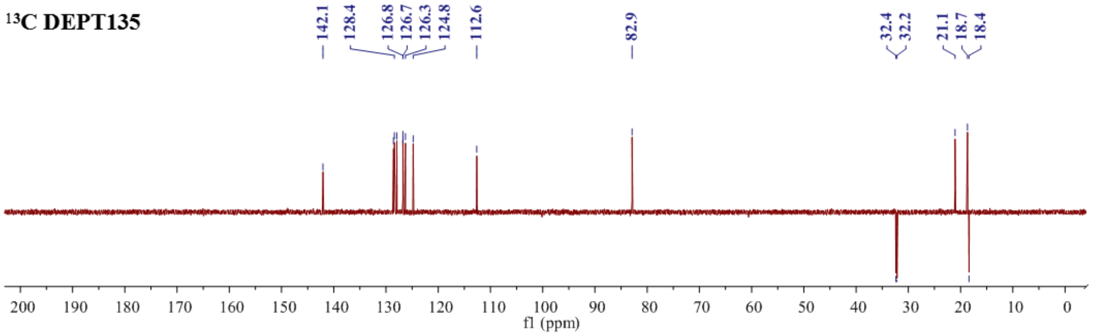
^{13}C DEPT135
142.1 128.4 126.8 126.7 126.3 124.8 112.6
82.9
32.4 32.2
21.1 18.7 18.4
fl (ppm)

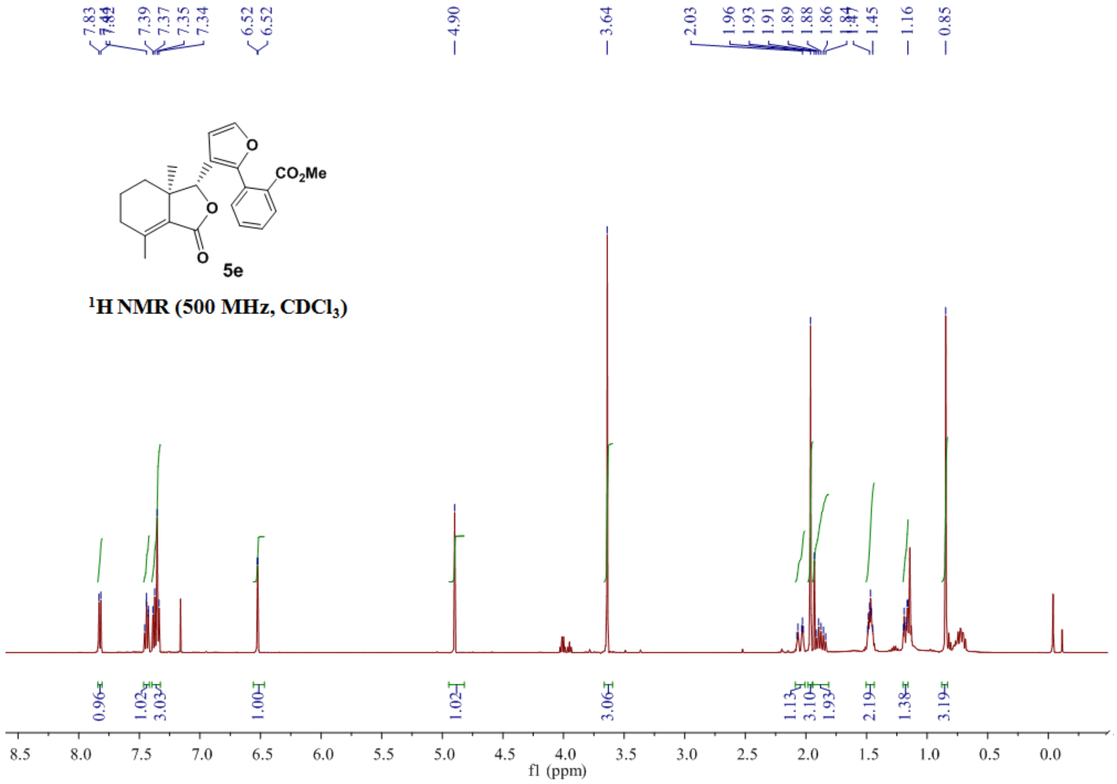
CO2Me
O
O
O
5e
1H NMR (500 MHz, CDCl3)
f1 (ppm)

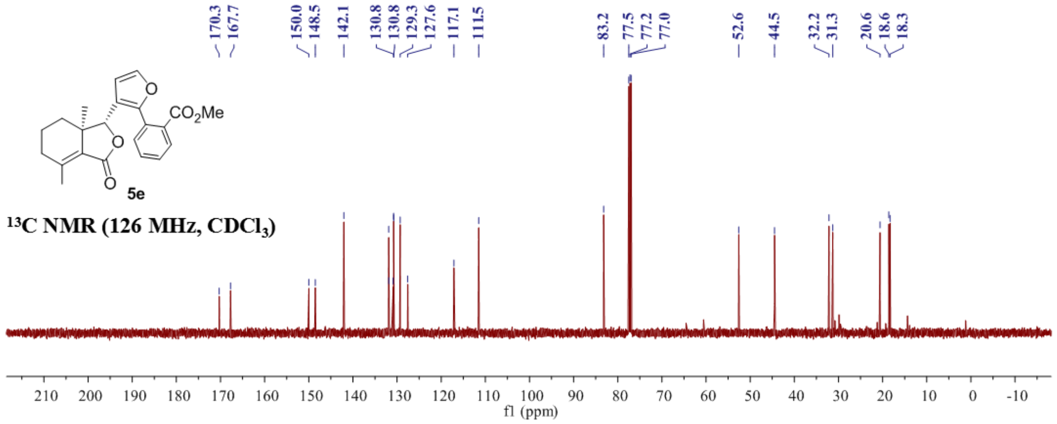
CO2Me
O
O
O
5e
13C NMR (126 MHz, CDCl3)
f1 (ppm)

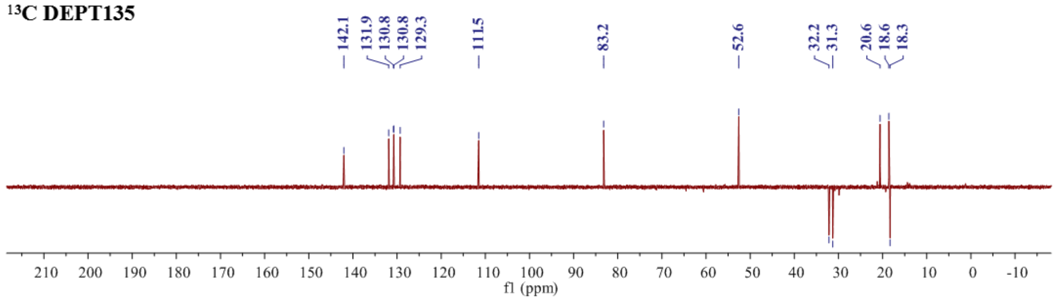
13C DEPT135
f1 (ppm)

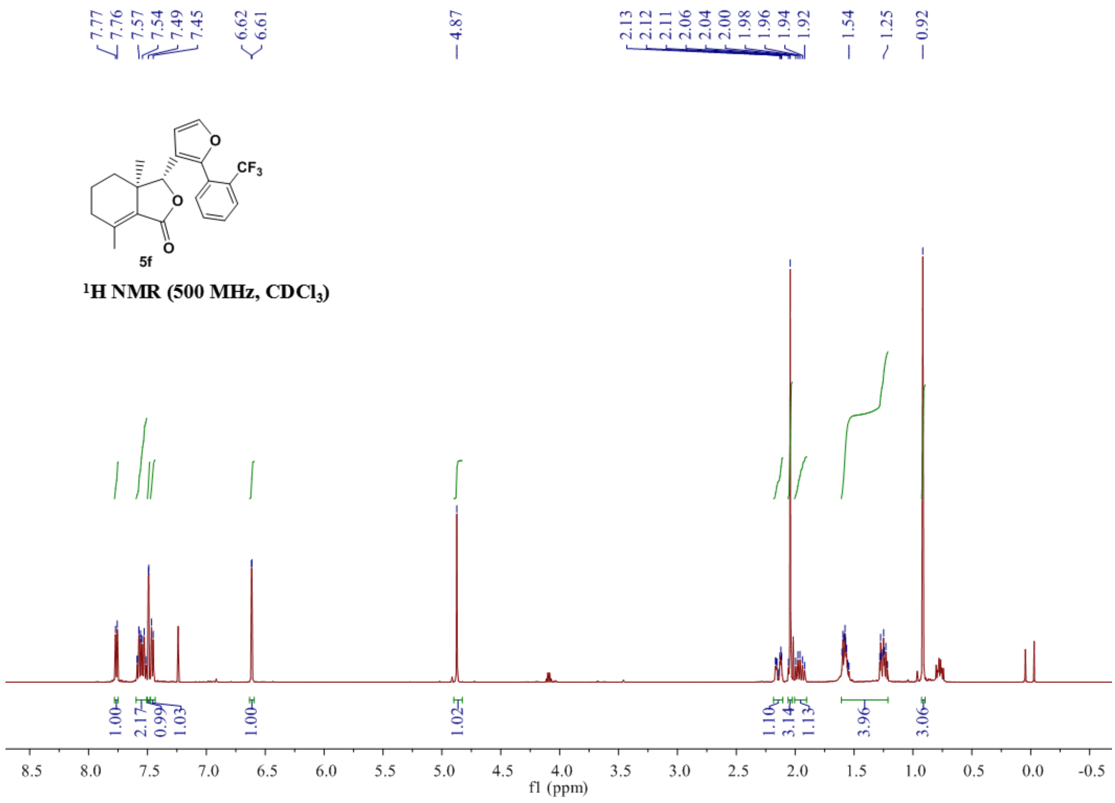

7.77
7.76
7.57
7.54
7.49
7.45
6.62
6.61
4.87
2.13
2.12
2.11
2.06
2.04
2.00
1.98
1.96
1.94
1.92
1.54
1.25
0.92
CF3
O
O
O
5f
1H NMR (500 MHz, CDCl3)
1.00
2.17
0.99
1.03
1.00
1.02
1.10
3.14
1.13
3.96
3.06
fl (ppm)

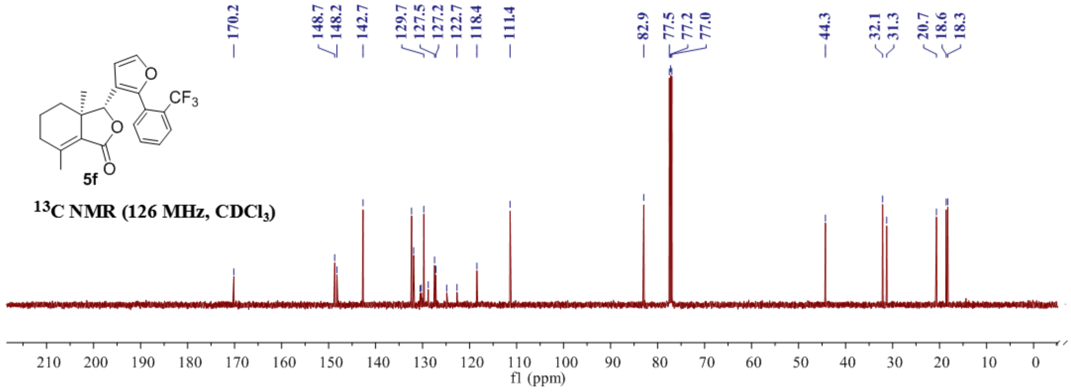

170.2
148.7
148.2
142.7
129.7
127.5
127.2
122.7
118.4
111.4
82.9
77.5
77.2
77.0
44.3
32.1
31.3
20.7
18.6
18.3
CF3
O
O
O
5f
13C NMR (126 MHz, CDCl3)
fl (ppm)

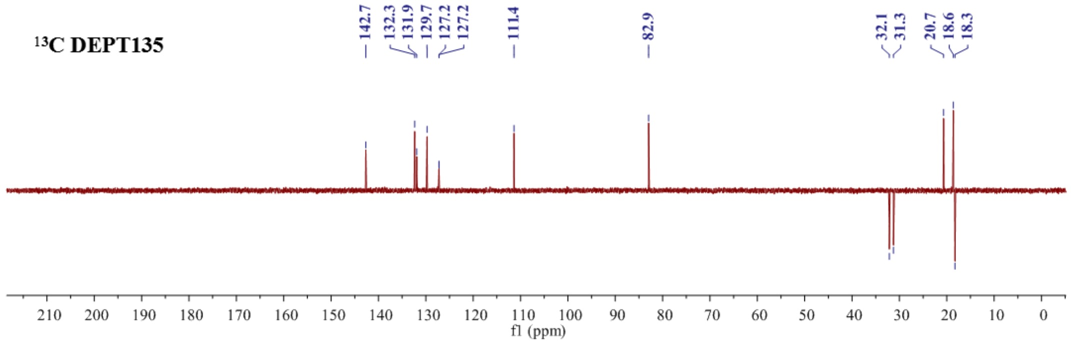

13C DEPT135
142.7
132.3
131.9
129.7
127.2
127.2
111.4
82.9
32.1
31.3
20.7
18.6
18.3
fl (ppm)

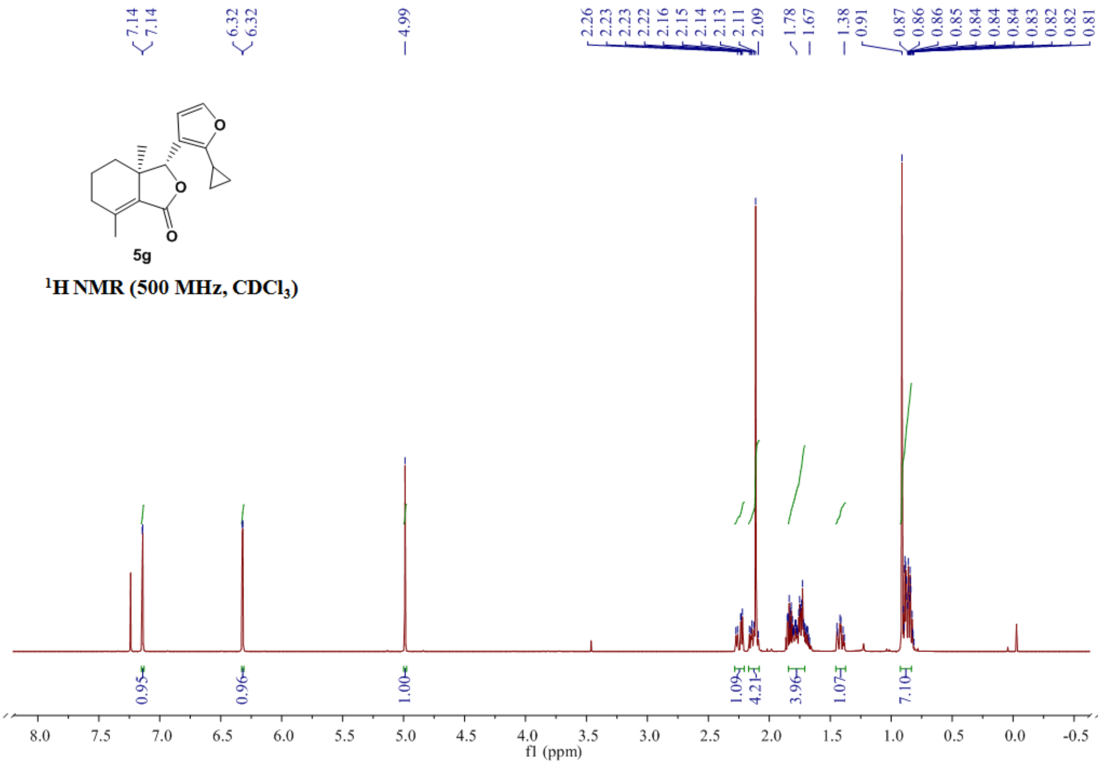
5g
1H NMR (500 MHz, CDCl3)
f1 (ppm)

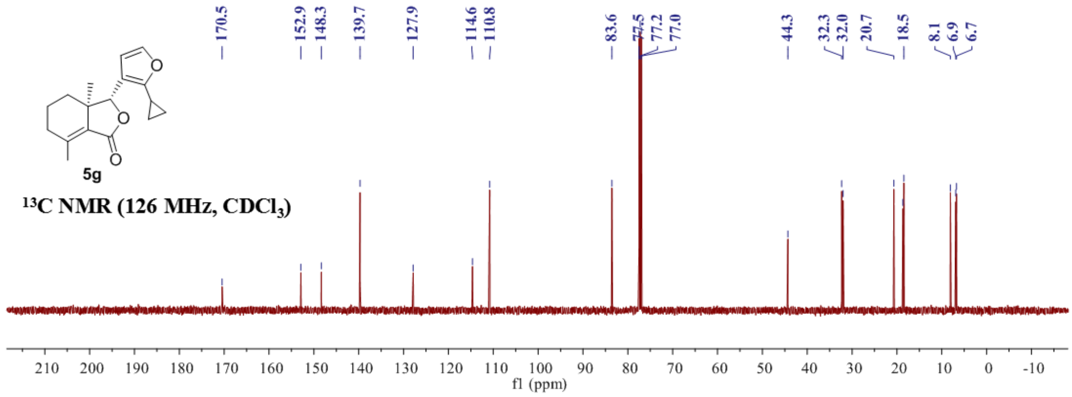
5g
13C NMR (126 MHz, CDCl3)
f1 (ppm)

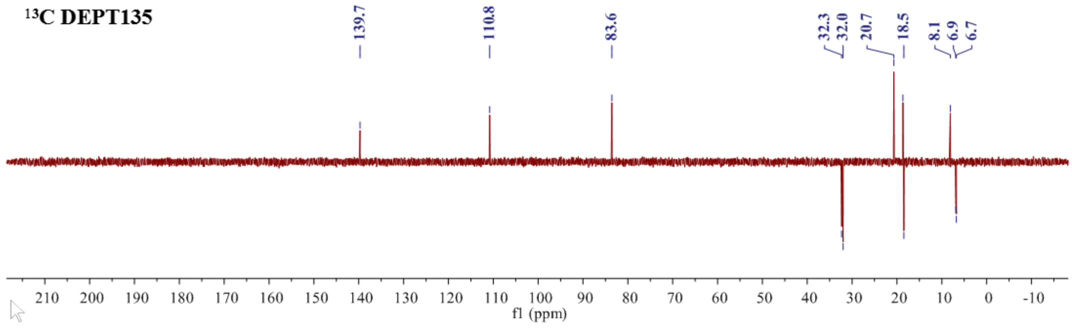
13C DEPT135
f1 (ppm)

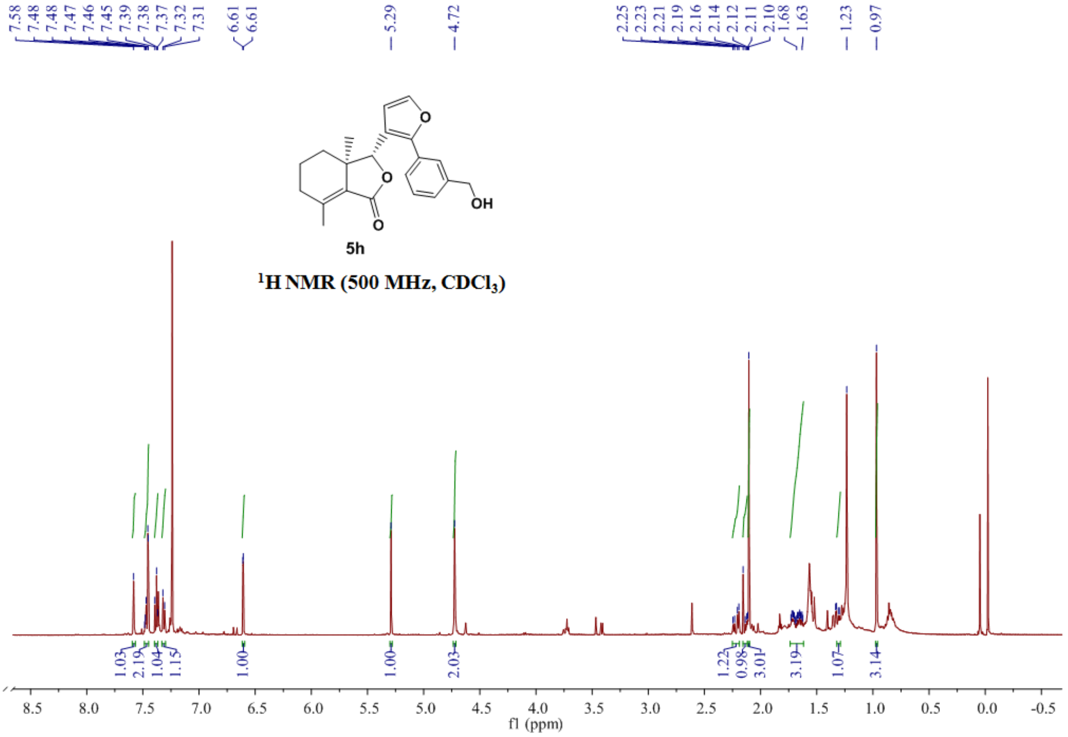
7.58 7.48 7.48 7.47 7.46 7.45 7.39 7.38 7.37 7.32 7.31
6.61 6.61
5.29
4.72
2.25 2.23 2.21 2.19 2.16 2.14 2.12 2.11 2.10 1.68 1.63
1.23
0.97
OH
O
5h
1H NMR (500 MHz, CDCl3)
1.03 2.19 1.04 1.15 1.00 1.00 2.03 1.22 0.98 3.01 3.19 1.07 3.14
f1 (ppm)

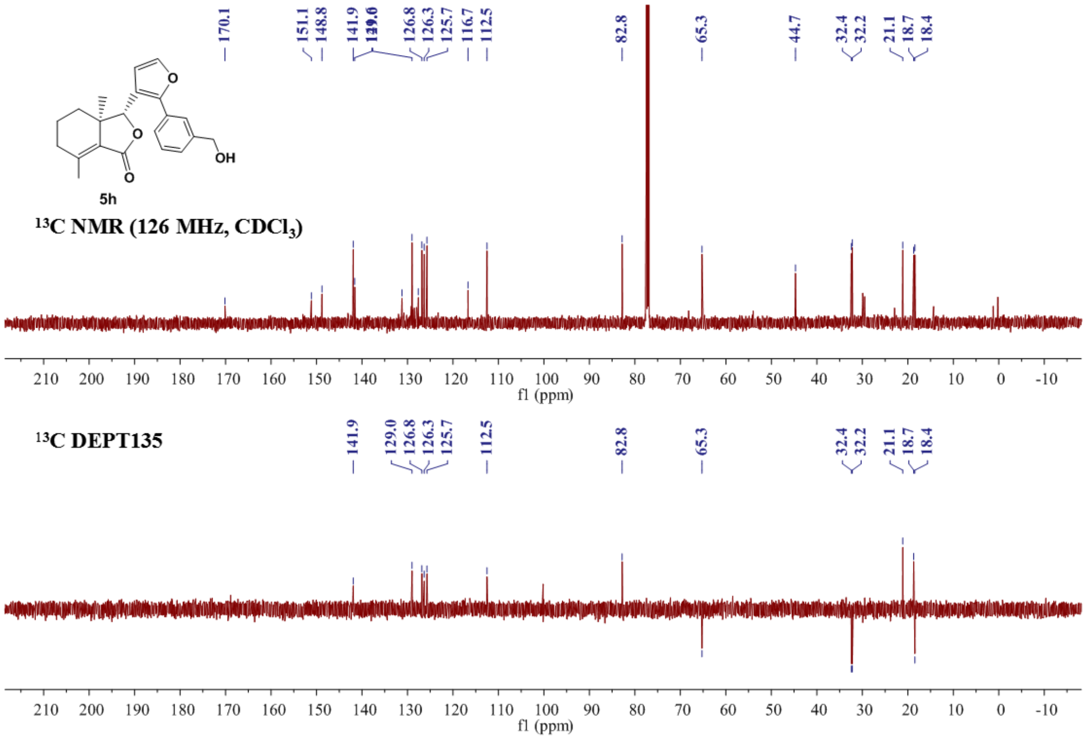
170.1
151.1 148.8
141.9 129.0 126.8 126.3 125.7 116.7 112.5
82.8
65.3
44.7
32.4 32.2
21.1 18.7 18.4
OH
O
5h
13C NMR (126 MHz, CDCl3)
f1 (ppm)
13C DEPT135
141.9
129.0 126.8 126.3 125.7
112.5
82.8
65.3
32.4 32.2
21.1 18.7 18.4
f1 (ppm)

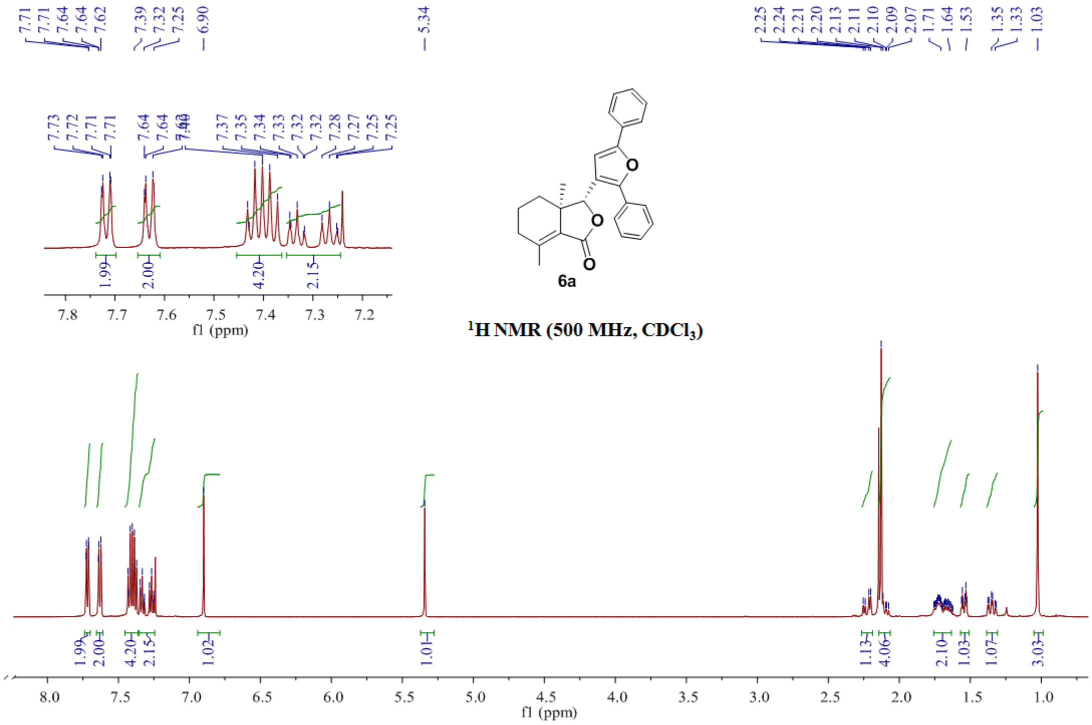
7.71 7.71 7.64 7.64 7.62 7.39 7.32 7.25 6.90
5.34
2.25 2.24 2.21 2.20 2.13 2.11 2.10 2.09 2.07 1.71 1.64 1.53 1.35 1.33 1.03
7.73 7.72 7.71 7.71 7.64 7.64 7.37 7.35 7.34 7.33 7.32 7.32 7.28 7.27 7.25 7.25
1.99 2.00 4.20 2.15
f1 (ppm)
O
O
O
6a
^{1}H NMR (500 MHz, CDCl$_3$)
1.99 2.00 4.20 2.15 1.02 1.01 1.13 4.06 2.10 1.03 1.07 3.03
f1 (ppm)

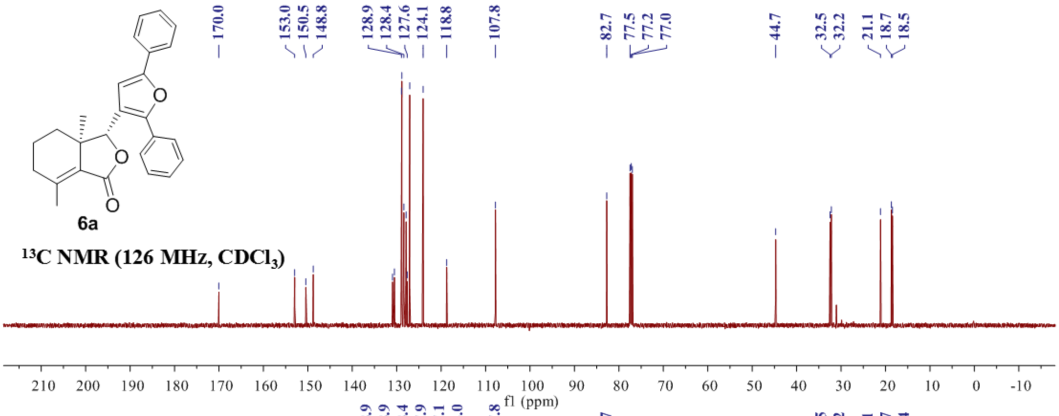
170.0
153.0 150.5 148.8
128.9 128.4 127.6 124.1 118.8
107.8
82.7 77.5 77.2 77.0
44.7
32.5 32.2 21.1 18.7 18.5
O
O
O
6a
^{13}C NMR (126 MHz, CDCl$_3$)
f1 (ppm)

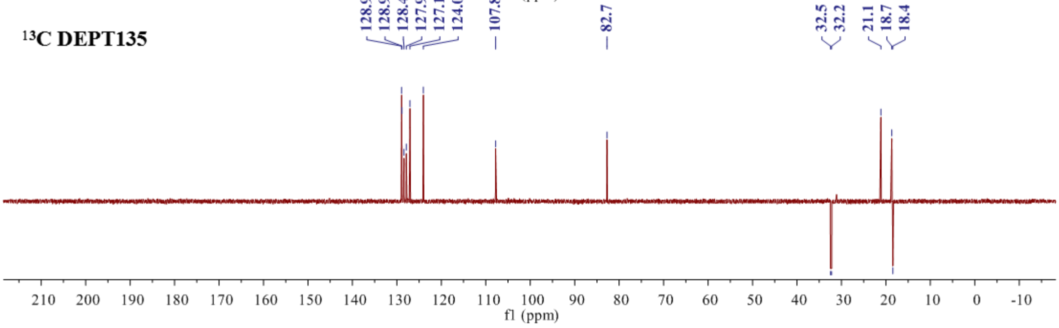
128.9 128.9 128.4 127.9 127.1 124.0
107.8
82.7
32.5 32.2 21.1 18.7 18.4
^{13}C DEPT135
f1 (ppm)

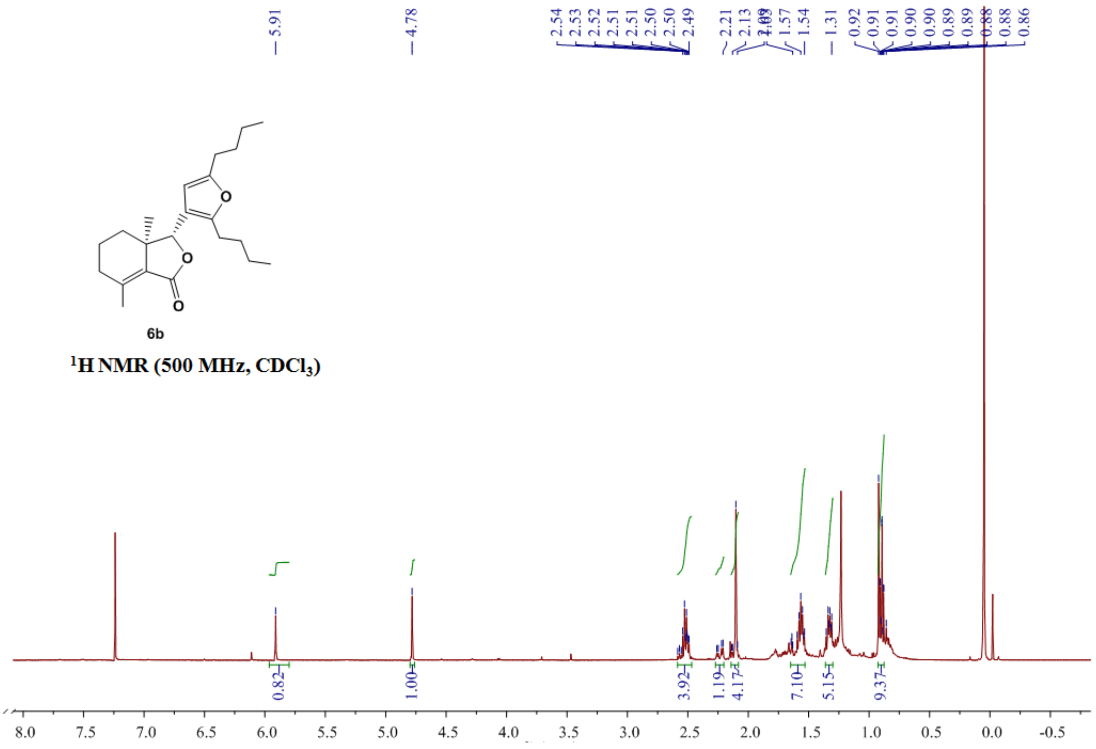

6b
¹H NMR (500 MHz, CDCl₃)

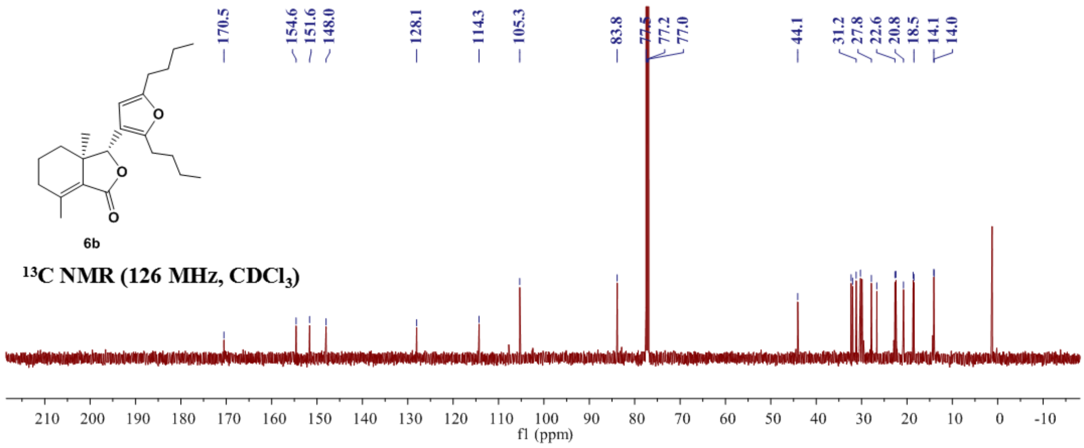

6b
¹³C NMR (126 MHz, CDCl₃)
f1 (ppm)

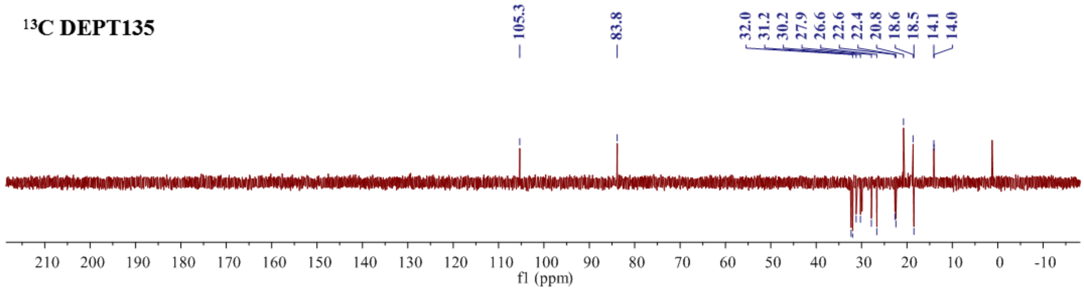

¹³C DEPT135
f1 (ppm)

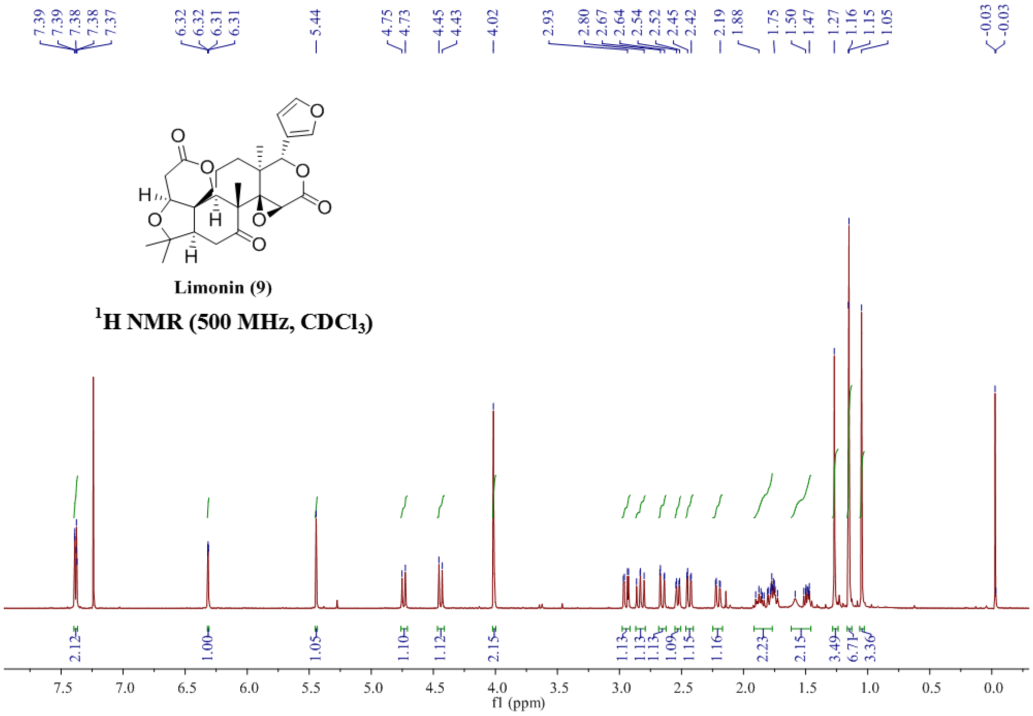

7.39
7.39
7.38
7.38
7.37
6.32
6.32
6.31
6.31
5.44
4.75
4.73
4.45
4.43
4.02
2.93
2.80
2.67
2.64
2.54
2.52
2.45
2.42
2.19
1.88
1.75
1.50
1.47
1.27
1.16
1.15
1.05
-0.03
-0.03
Limonin (9)
^{1}H NMR (500 MHz, CDCl$_3$)
2.12
1.00
1.05
1.10
1.12
2.15
1.13
1.13
1.13
1.09
1.15
1.16
2.23
2.15
3.49
6.71
3.36
f1 (ppm)

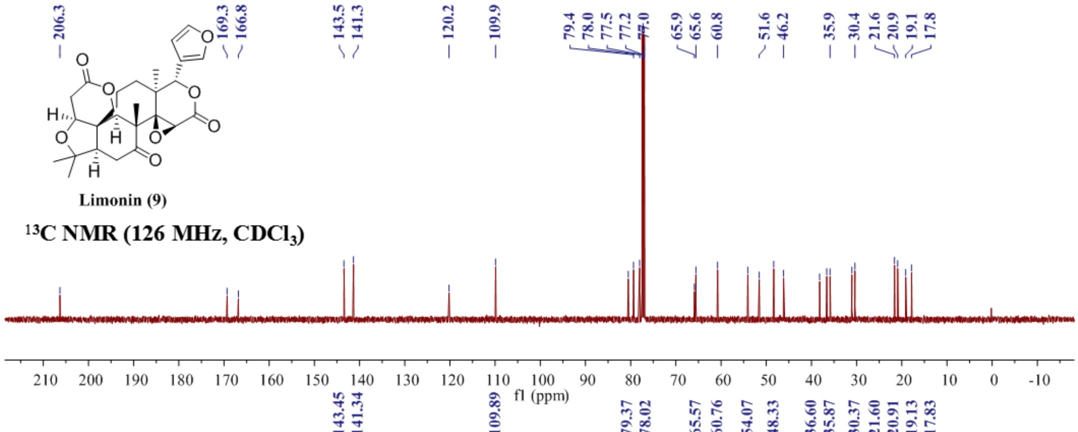

206.3
169.3
166.8
143.5
141.3
120.2
109.9
79.4
78.0
77.5
77.2
77.0
65.9
65.6
60.8
51.6
46.2
35.9
30.4
21.6
20.9
19.1
17.8
Limonin (9)
^{13}C NMR (126 MHz, CDCl$_3$)
f1 (ppm)

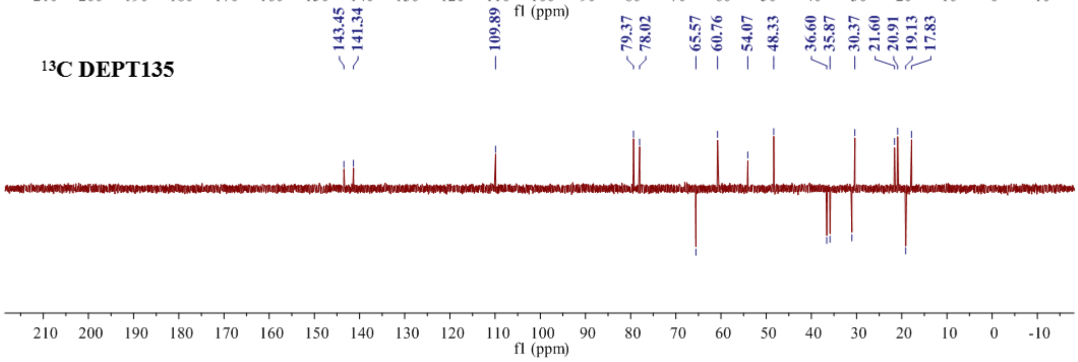

143.45
141.34
109.89
79.37
78.02
65.57
60.76
54.07
48.33
36.60
35.87
30.37
21.60
20.91
19.13
17.83
^{13}C DEPT135
f1 (ppm)

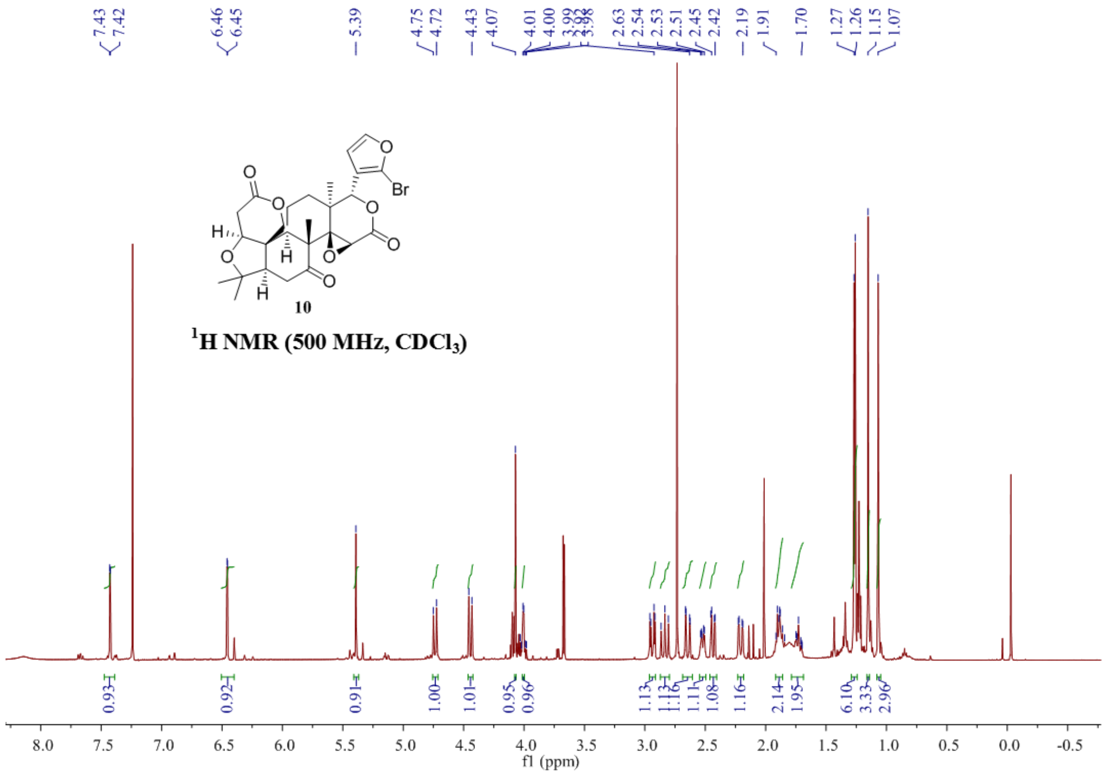
10
^{1}H NMR (500 MHz, $CDCl_3$)
f1 (ppm)

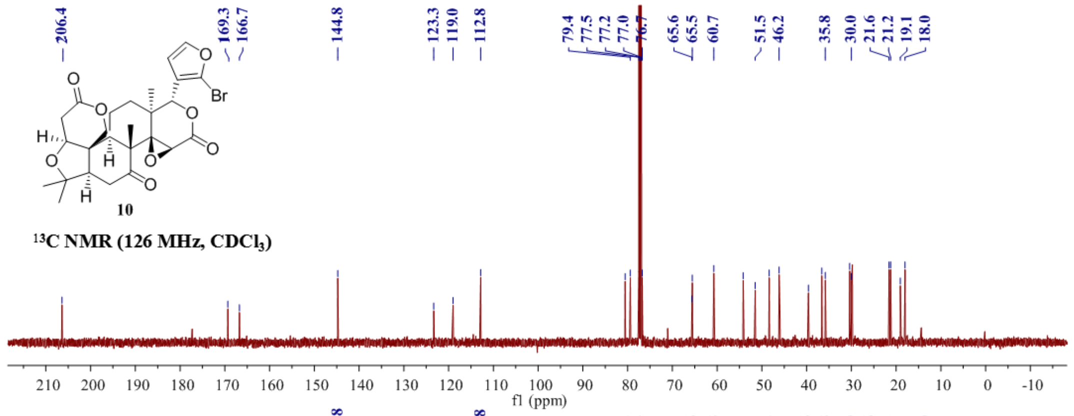
10
^{13}C NMR (126 MHz, $CDCl_3$)
f1 (ppm)

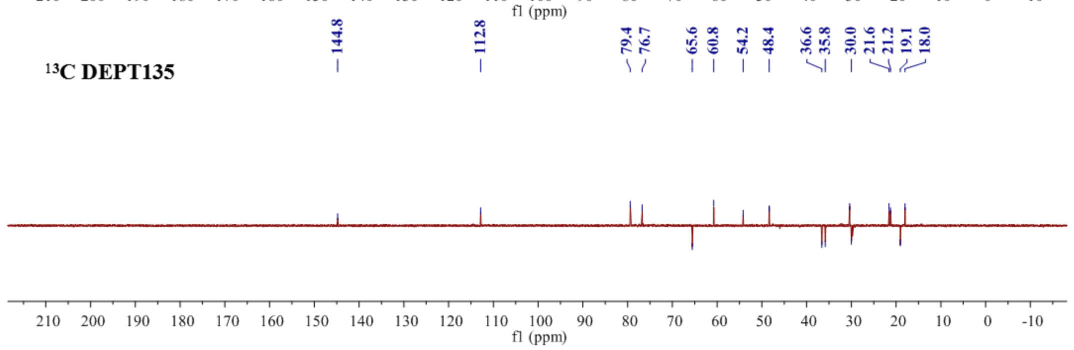
^{13}C DEPT135
f1 (ppm)

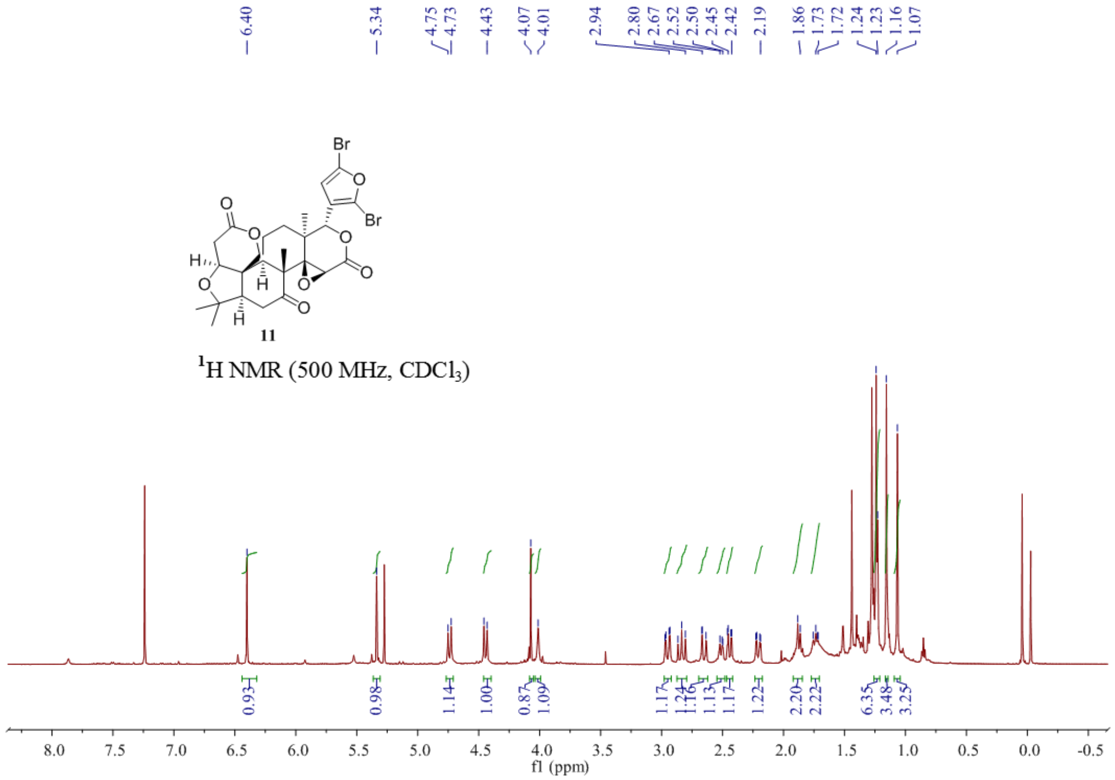

6.40
5.34
4.75
4.73
4.43
4.07
4.01
2.94
2.80
2.67
2.52
2.50
2.45
2.42
2.19
1.86
1.73
1.72
1.24
1.23
1.16
1.07
Br
O
Br
O
O
O
O
H
H
O
H
O
O
11
^{1}H NMR (500 MHz, $CDCl_3$)
0.93
0.98
1.14
1.00
0.87
1.09
1.17
1.24
1.16
1.13
1.17
1.22
2.20
2.22
6.35
3.48
3.25
8.0
7.5
7.0
6.5
6.0
5.5
5.0
4.5
4.0
3.5
3.0
2.5
2.0
1.5
1.0
0.5
0.0
-0.5
f1 (ppm)

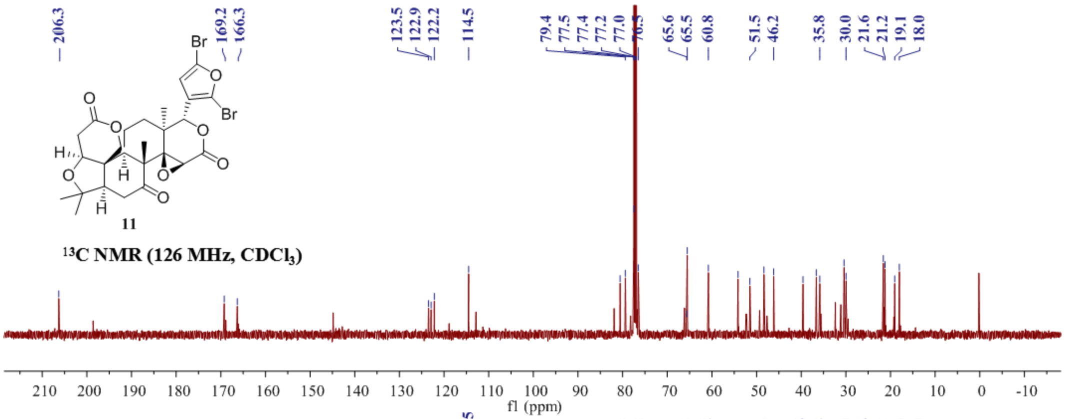

206.3
169.2
166.3
123.5
122.9
122.2
114.5
79.4
77.5
77.4
77.2
77.0
76.5
65.6
65.5
60.8
51.5
46.2
35.8
30.0
21.6
21.2
19.1
18.0
Br
O
Br
O
O
O
O
H
H
O
H
O
O
11
^{13}C NMR (126 MHz, $CDCl_3$)
210
200
190
180
170
160
150
140
130
120
110
100
90
80
70
60
50
40
30
20
10
0
-10
f1 (ppm)

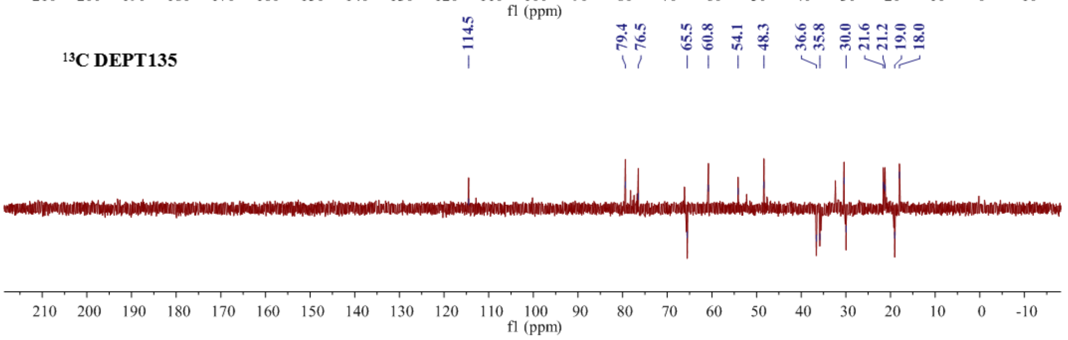

^{13}C DEPT135
114.5
79.4
76.5
65.5
60.8
54.1
48.3
36.6
35.8
30.0
21.6
21.2
19.0
18.0
210
200
190
180
170
160
150
140
130
120
110
100
90
80
70
60
50
40
30
20
10
0
-10
f1 (ppm)

4. (HR)-ESI-MS spectra

3: HRMS (ESI) m/z calcd for $C_{14}H_{18}BrO_3^+$ $(M+H)^+$ 313.04338, found 313.04340.

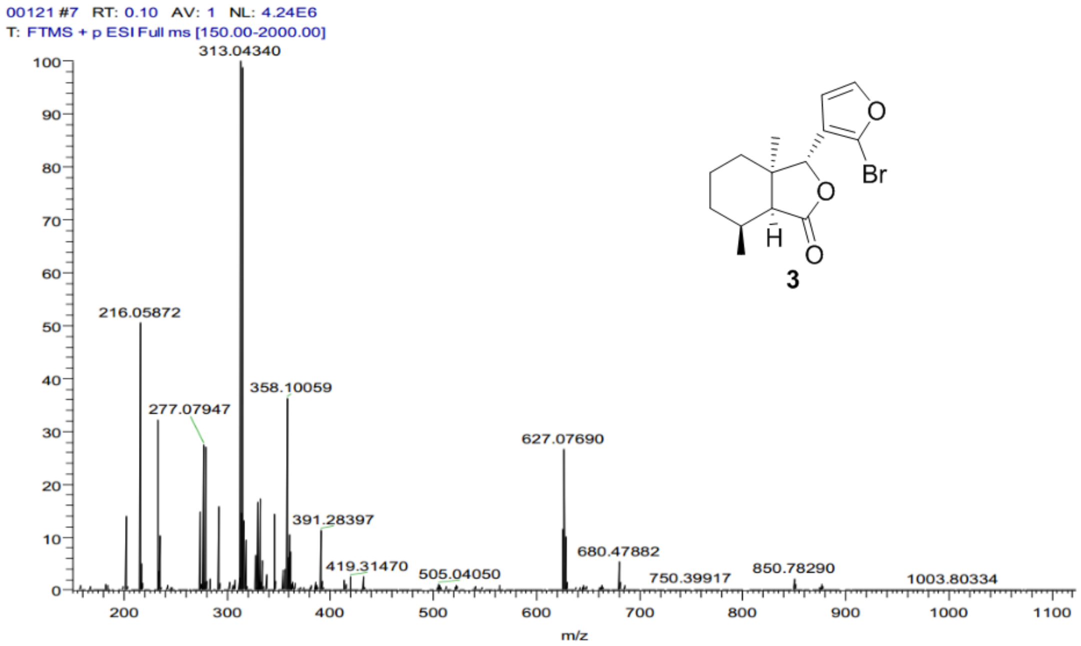

4: HRMS (ESI) m/z calcd for $C_{14}H_{17}Br_2O_3^+$ $(M+H)^+$ 390.95390, found 390.95395.

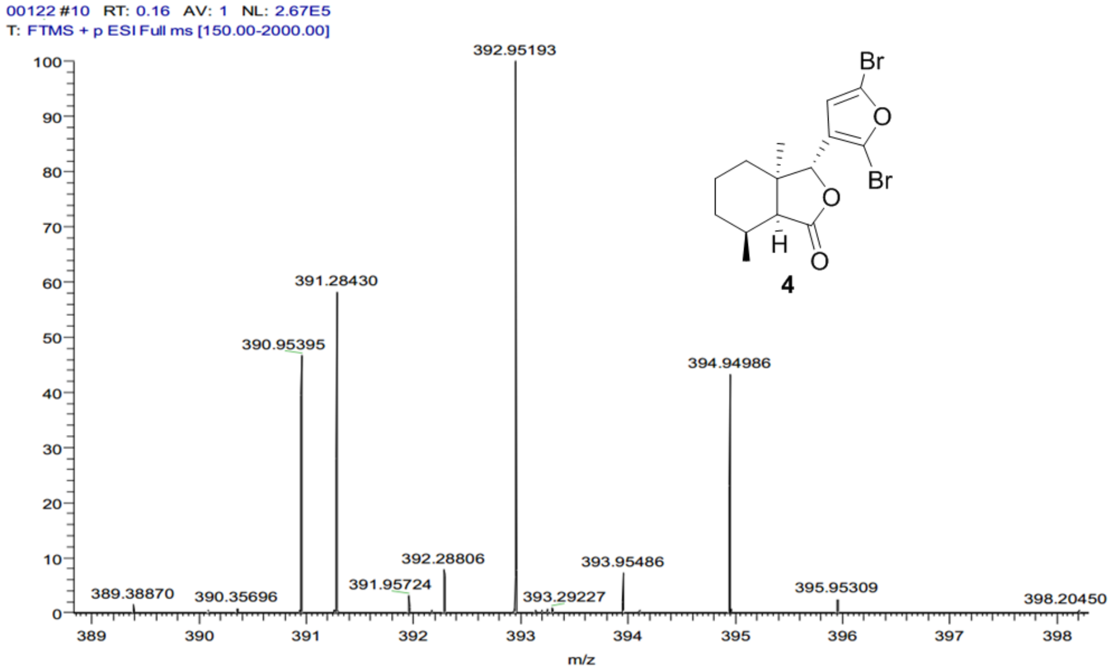

5: HRMS (ESI) m/z calcd for $C_{14}H_{16}BrO_3^+$ $(M+H)^+$ 311.02773, found 311.02747.

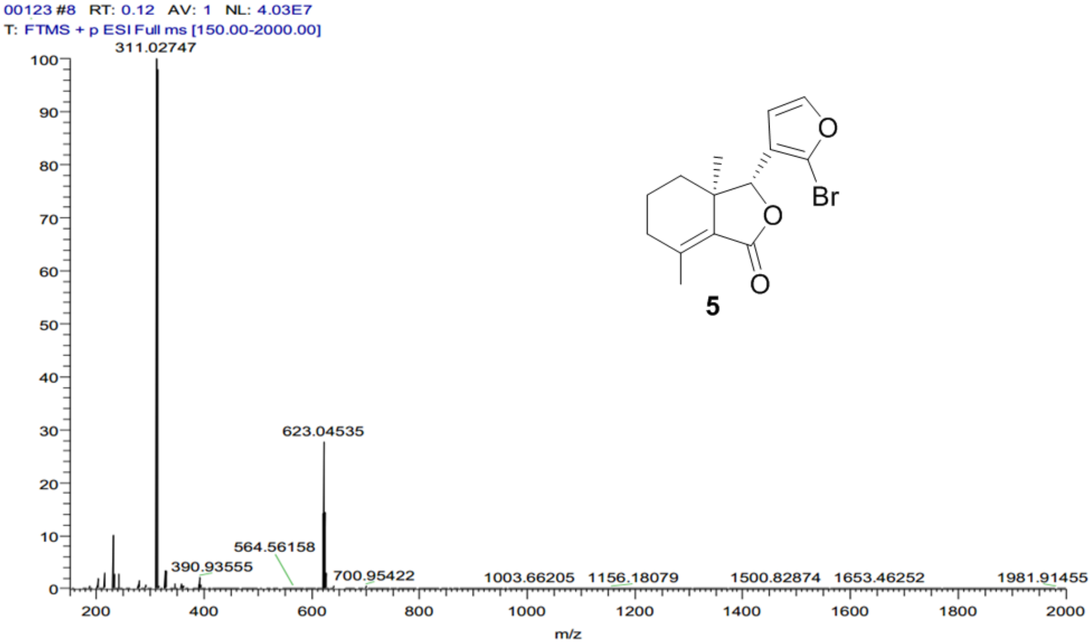

6: HRMS (ESI) m/z calcd for $C_{14}H_{15}Br_2O_3^+$ $(M+H)^+$ 388.93825, found 388.93832.

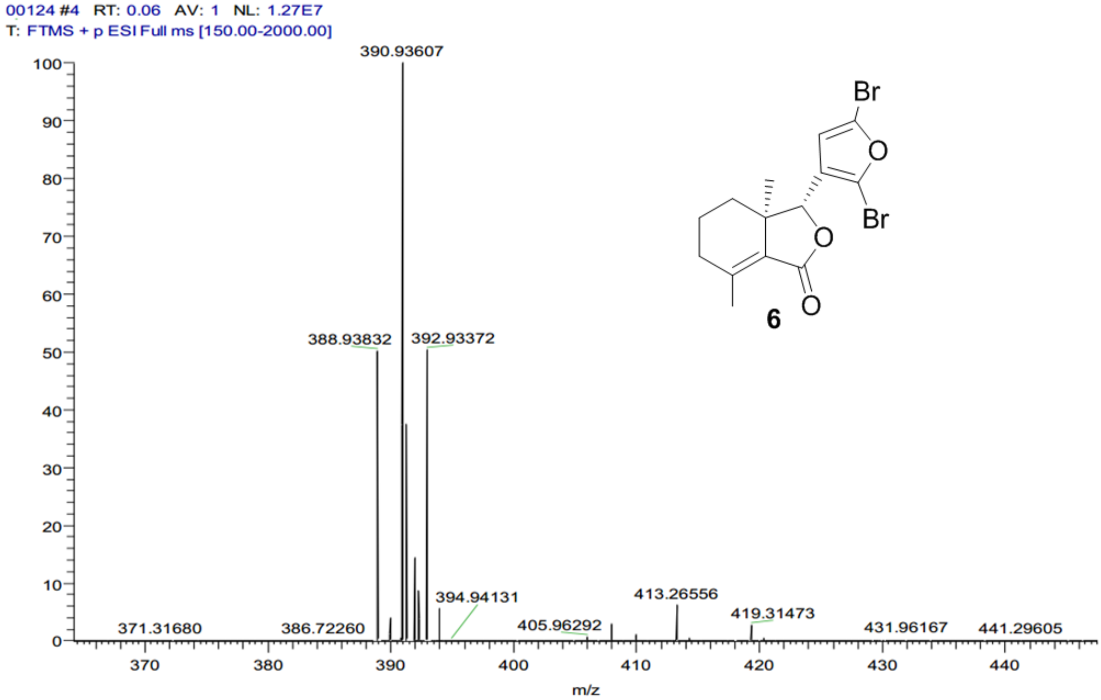

3a: HRMS (ESI) m/z calcd for $C_{20}H_{23}O_3^+$ (M+H)$^+$ 311.16417, found 311.16418.

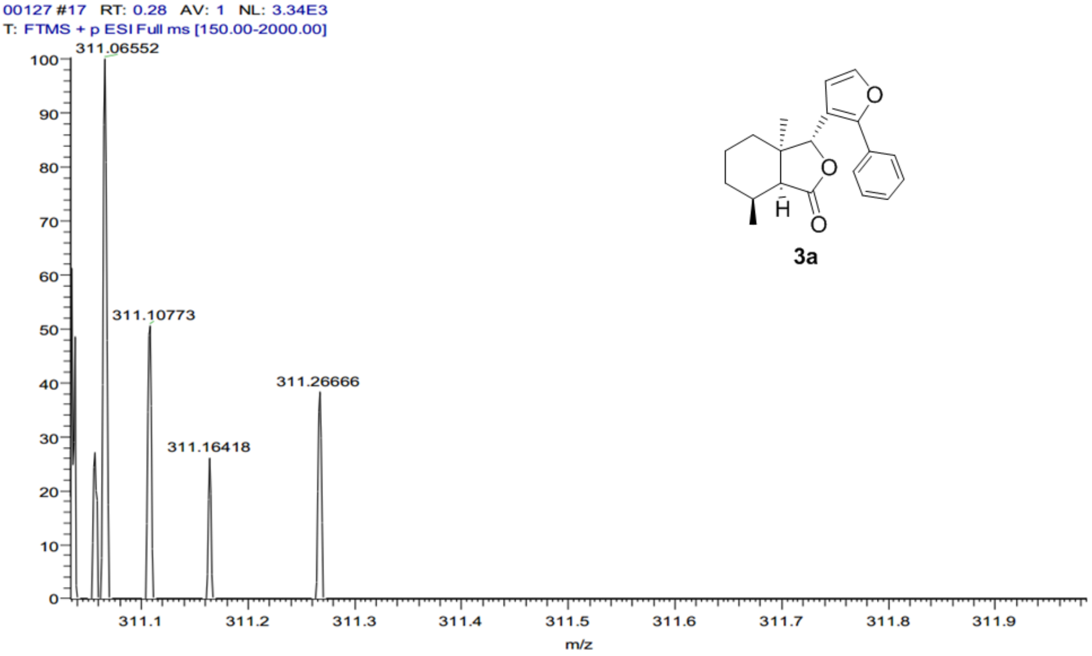

3b: HRMS (ESI) m/z calcd for $C_{18}H_{27}O_3^+$ (M+H)$^+$ 291.19547, found 291.19547.

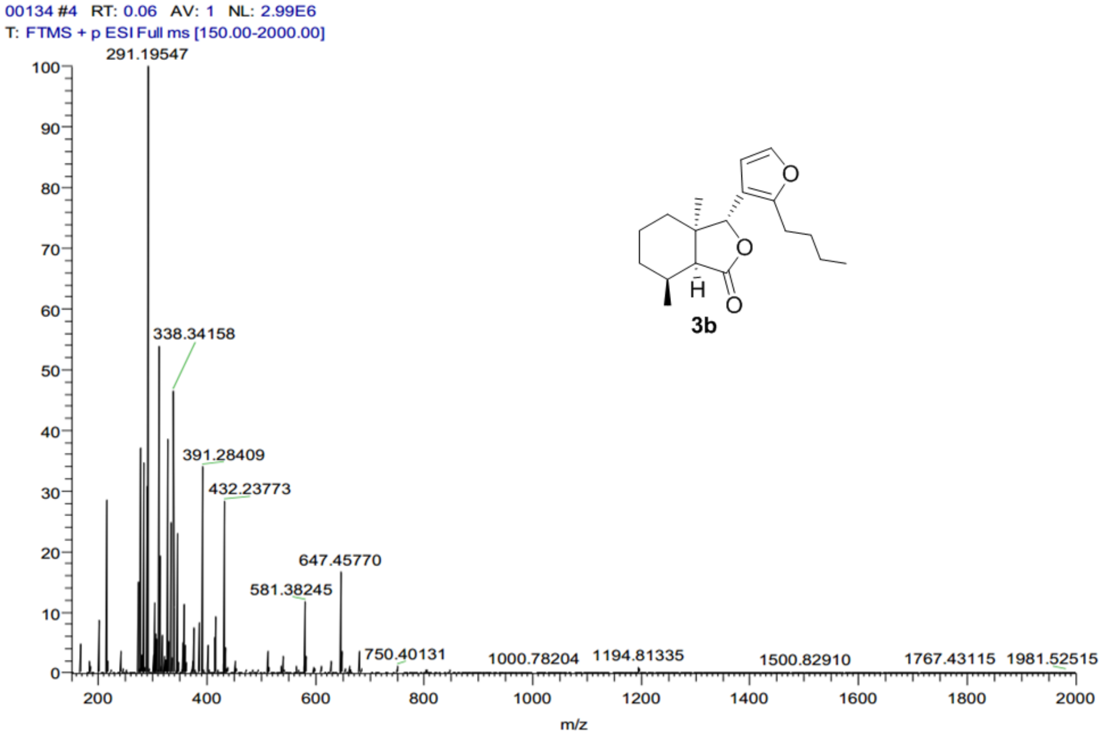

3c: HRMS (ESI) m/z calcd for $C_{22}H_{23}O_3^+$ (M+H)$^+$ 335.16417, found 335.16415.

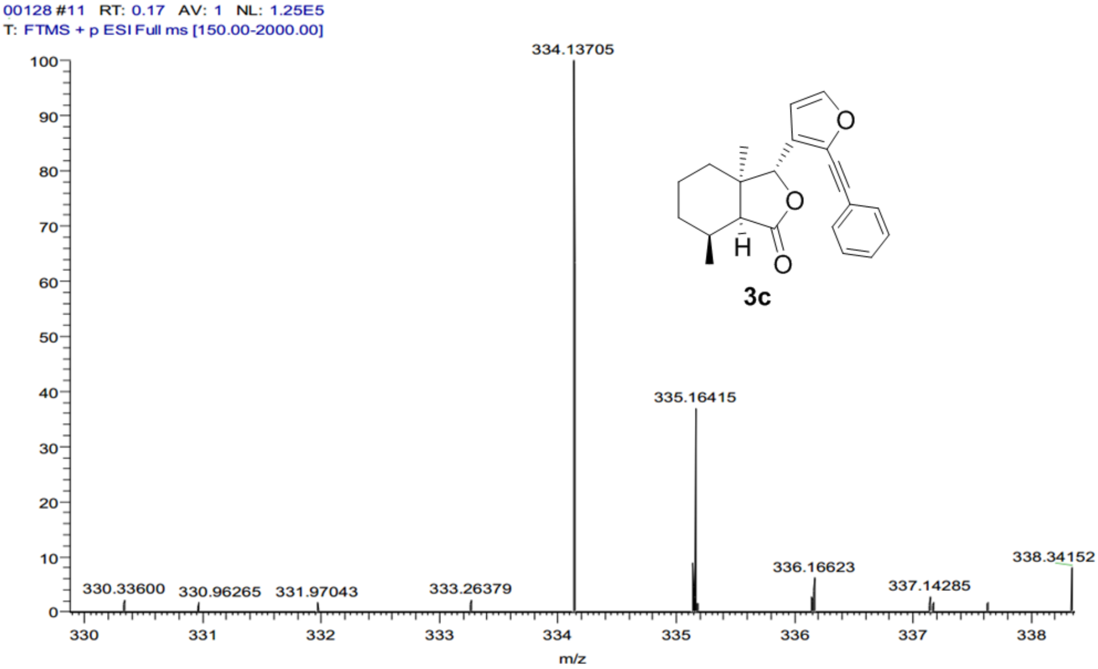

3d: HRMS (ESI) m/z calcd for $C_{24}H_{25}O_3^+$ (M+H)$^+$ 361.17982, found 361.17983.

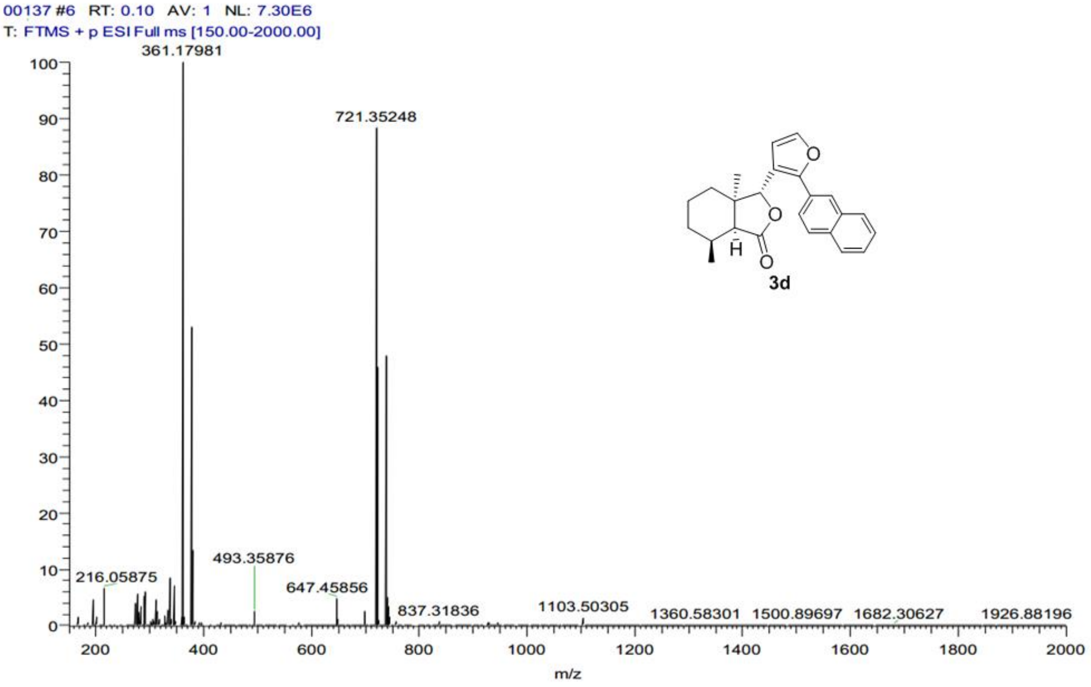

3e: HRMS (ESI) m/z calcd for $C_{22}H_{25}O_5^+$ $(M+H)^+$ 369.16965, found 369.16965.

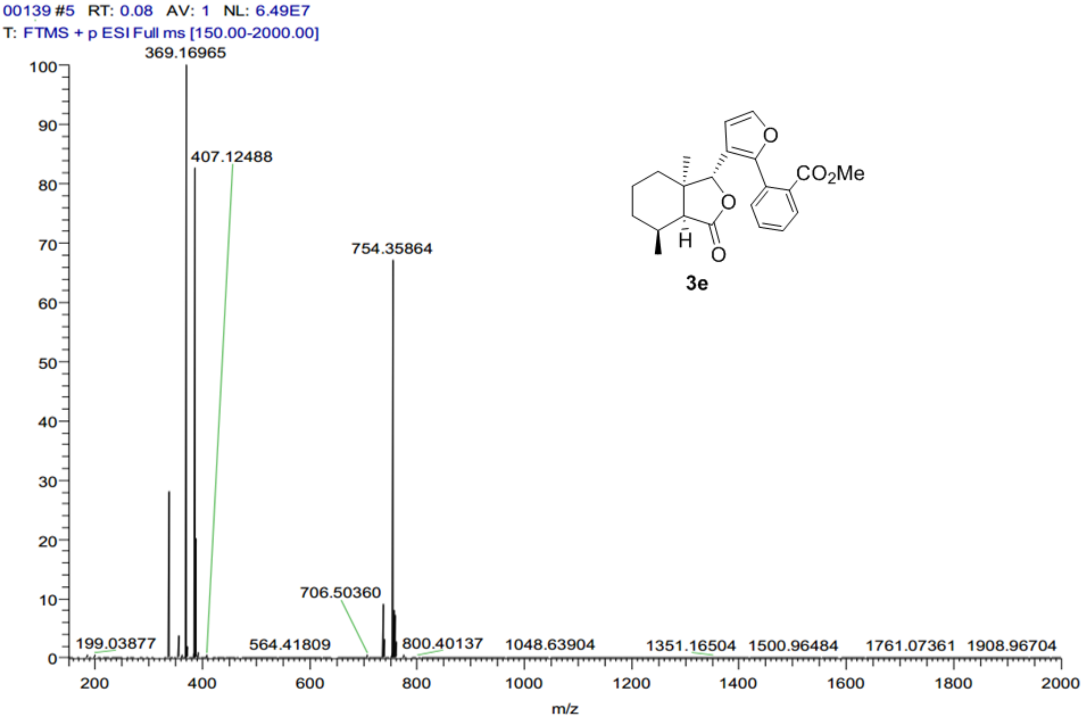

3f: HRMS (ESI) m/z calcd for $C_{21}H_{22}F_3O_3^+$ $(M+H)^+$ 379.15156, found 379.15152.

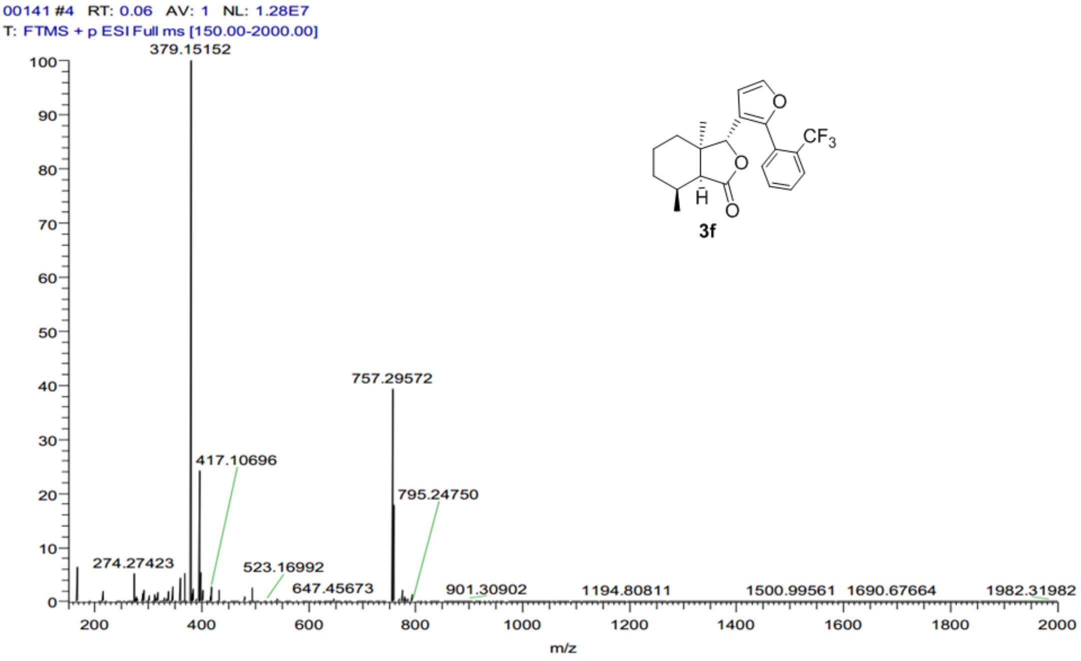

3g: HRMS (ESI) m/z calcd for $C_{17}H_{23}O_3^+$ $(M+H)^+$ 275.16417, found 275.16446.

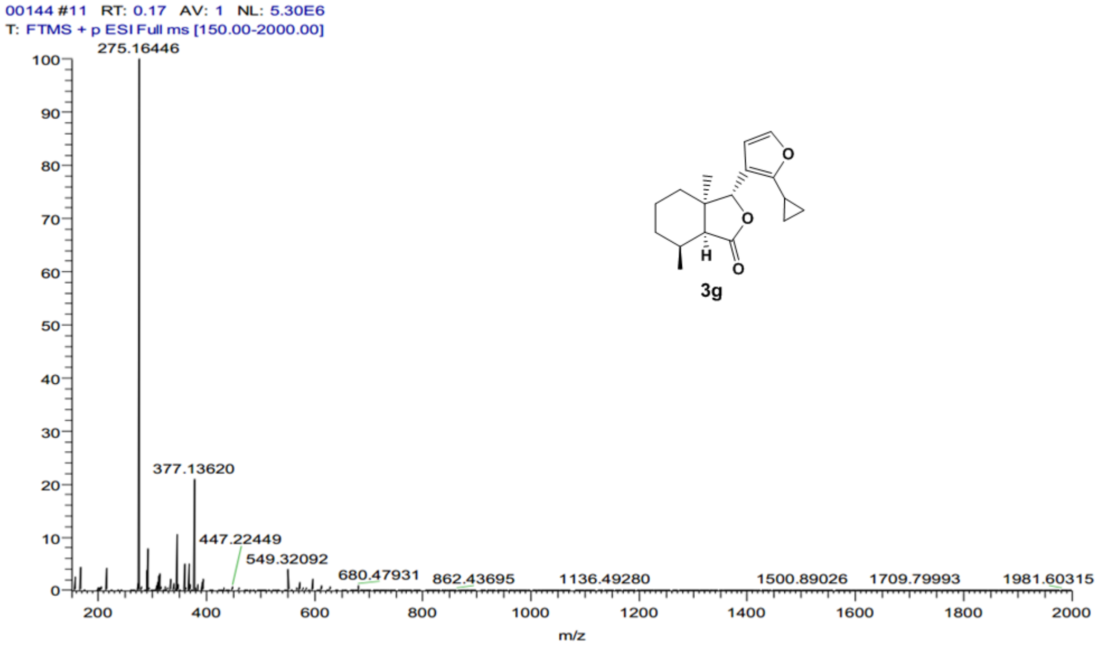

4a: HRMS (ESI) m/z calcd for $C_{26}H_{27}O_3^+$ $(M+H)^+$ 387.19547, found 387.19556.

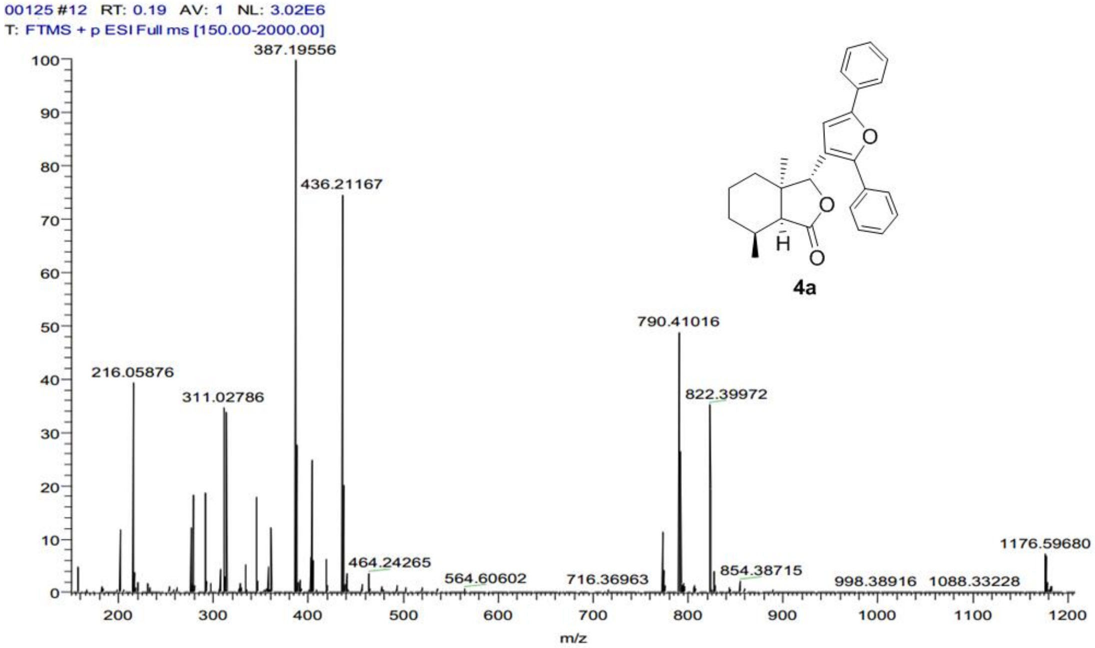

4b: HRMS (ESI) m/z calcd for $C_{22}H_{35}O_3^+$ $(M+H)^+$ 347.25807, found 347.25790.

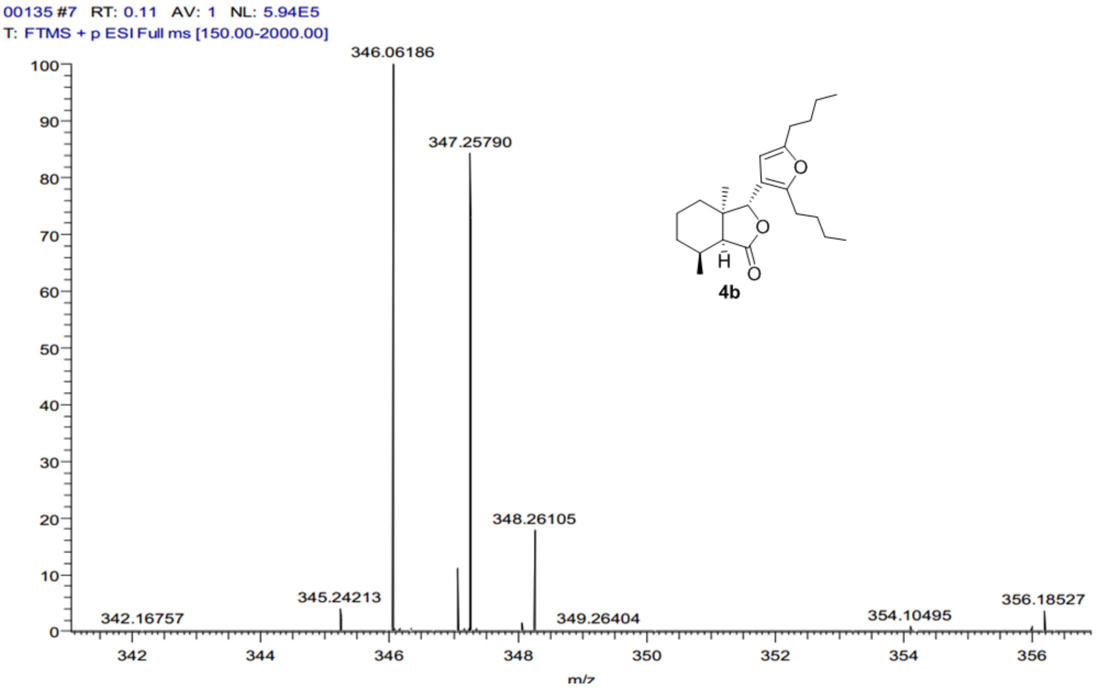

4c: HRMS (ESI) m/z calcd for $C_{30}H_{27}O_3^+$ $(M+H)^+$ 435.19547, found 435.19550.

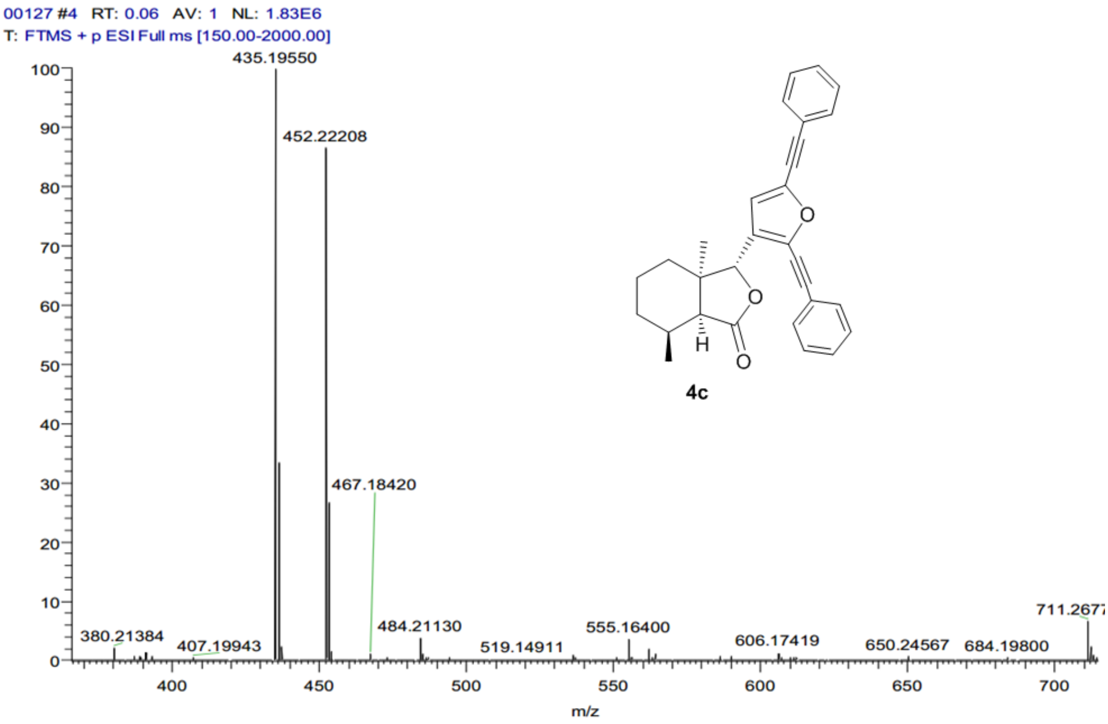

5a: HRMS (ESI) m/z calcd for $C_{20}H_{21}O_3^+$ $(M+H)^+$ 309.14852, found 309.14856.

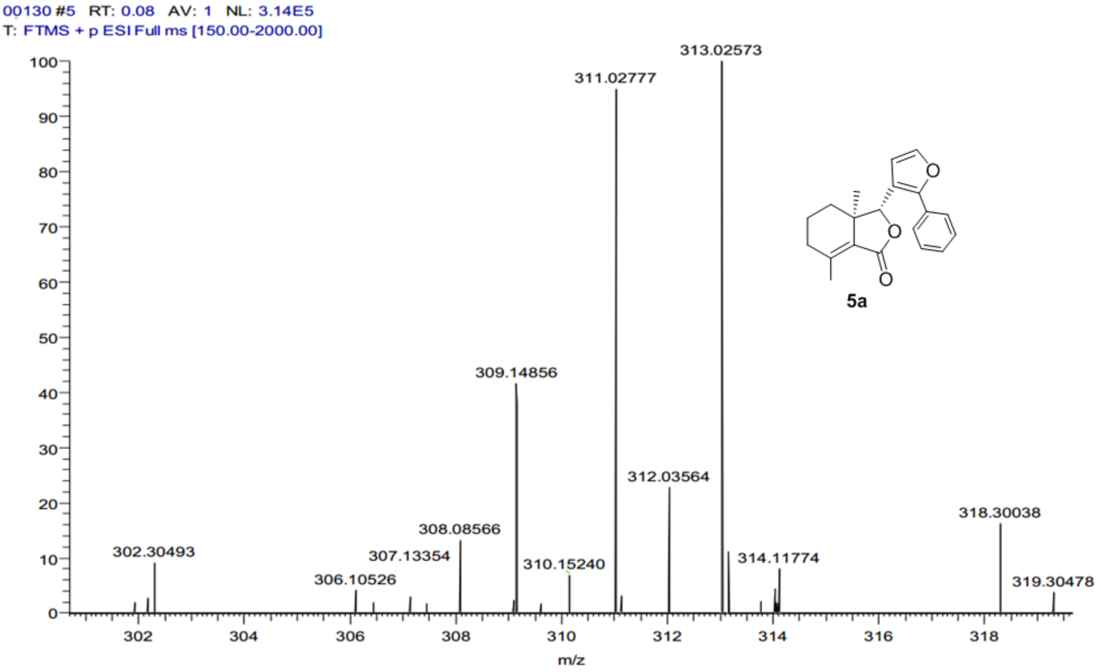

5b: HRMS (ESI) m/z calcd for $C_{18}H_{25}O_3^+$ $(M+H)^+$ 289.17982, found 289.17944.

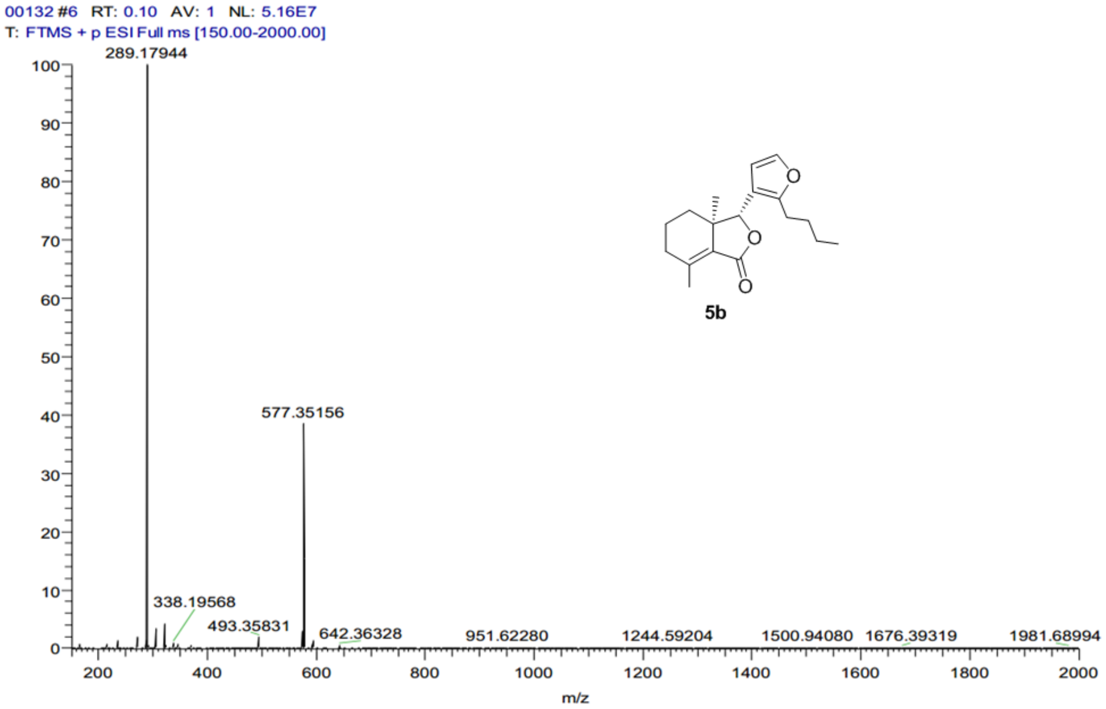

5c: HRMS (ESI) m/z calcd for $C_{22}H_{21}O_3^+$ $(M+H)^+$ 333.14852, found 333.14862.

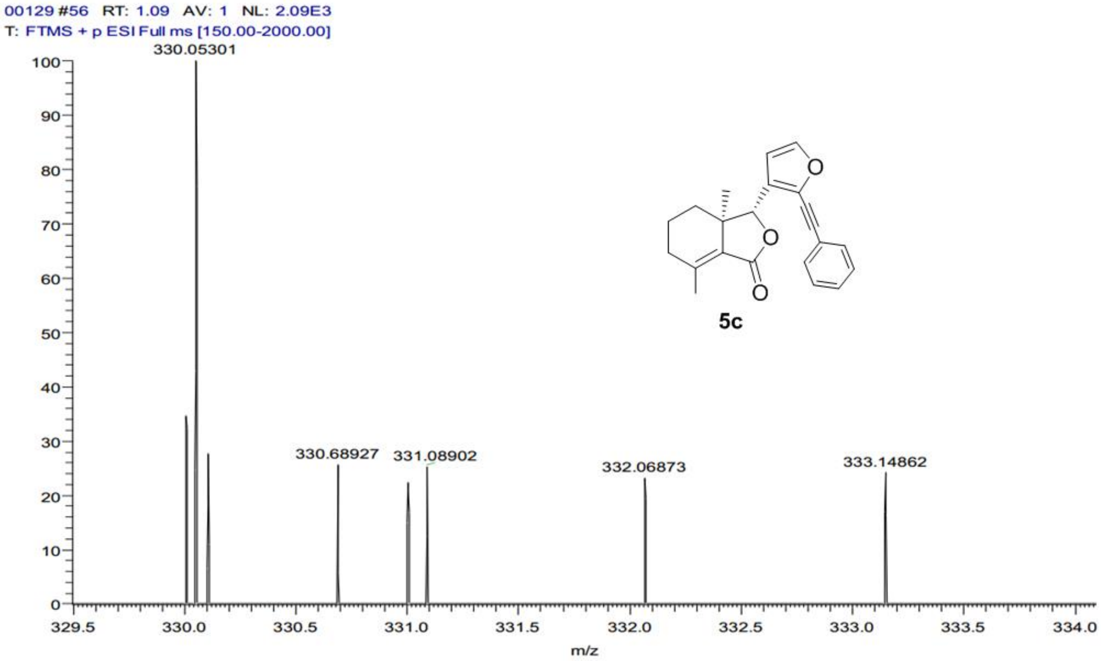

5d: HRMS (ESI) m/z calcd for $C_{24}H_{23}O_3^+$ $(M^+H)^+$ 359.16417, found 359.16415.

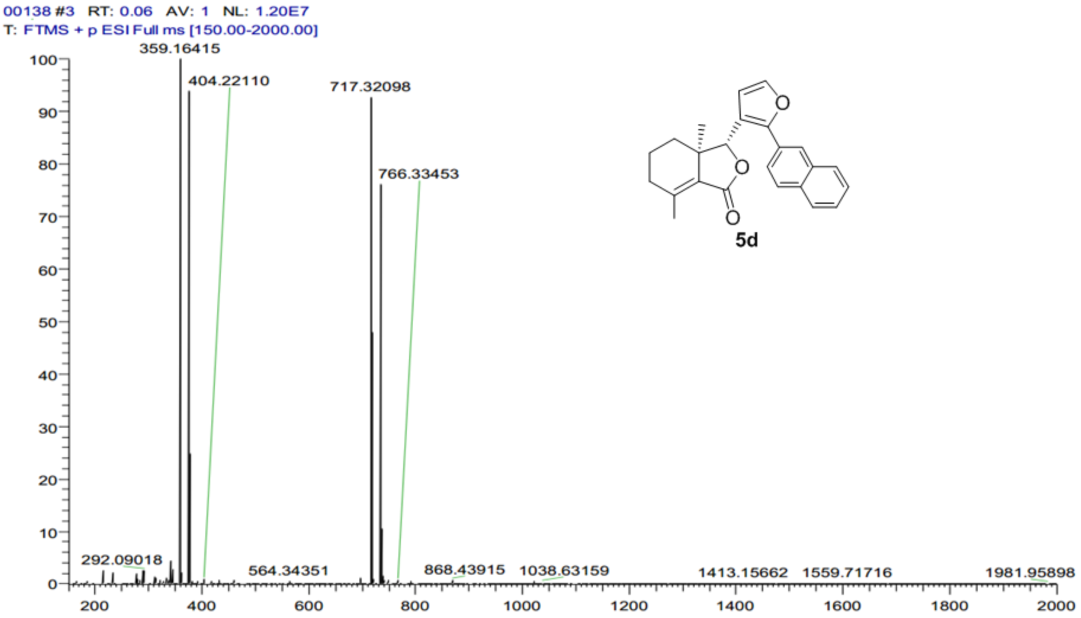

5e: HRMS (ESI) m/z calcd for $C_{22}H_{23}O_5^+$ $(M+H)^+$ 367.15400, found 367.15408.

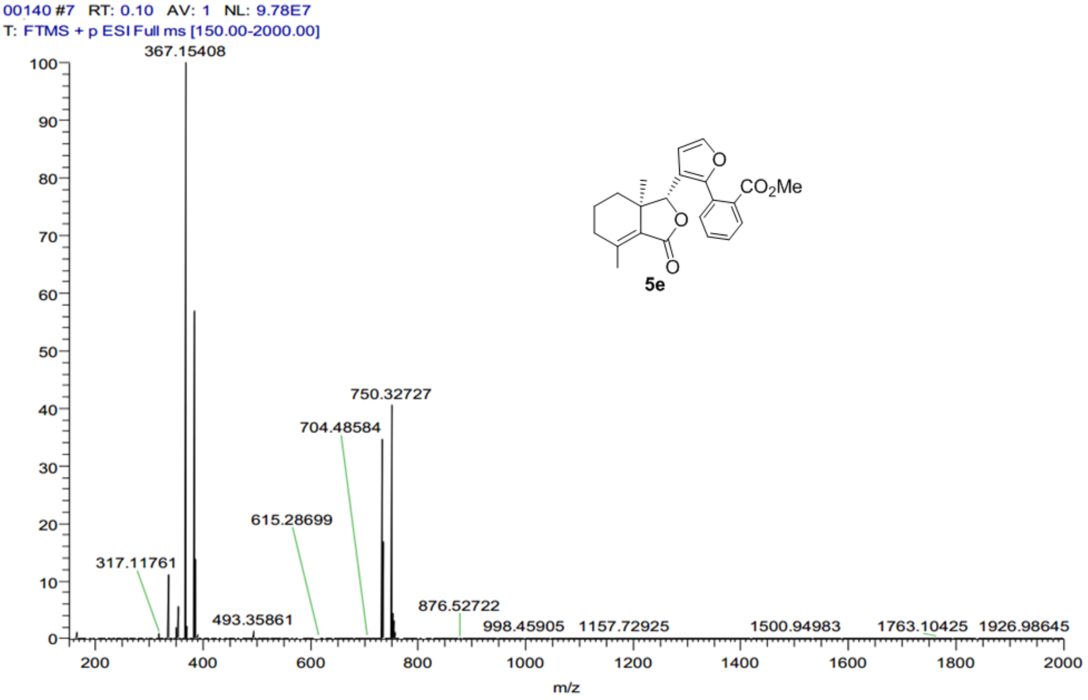

5f: HRMS (ESI) m/z calcd for $C_{21}H_{20}F_3O_3^+$ $(M+H)^+$ 377.13591, found 377.13593.

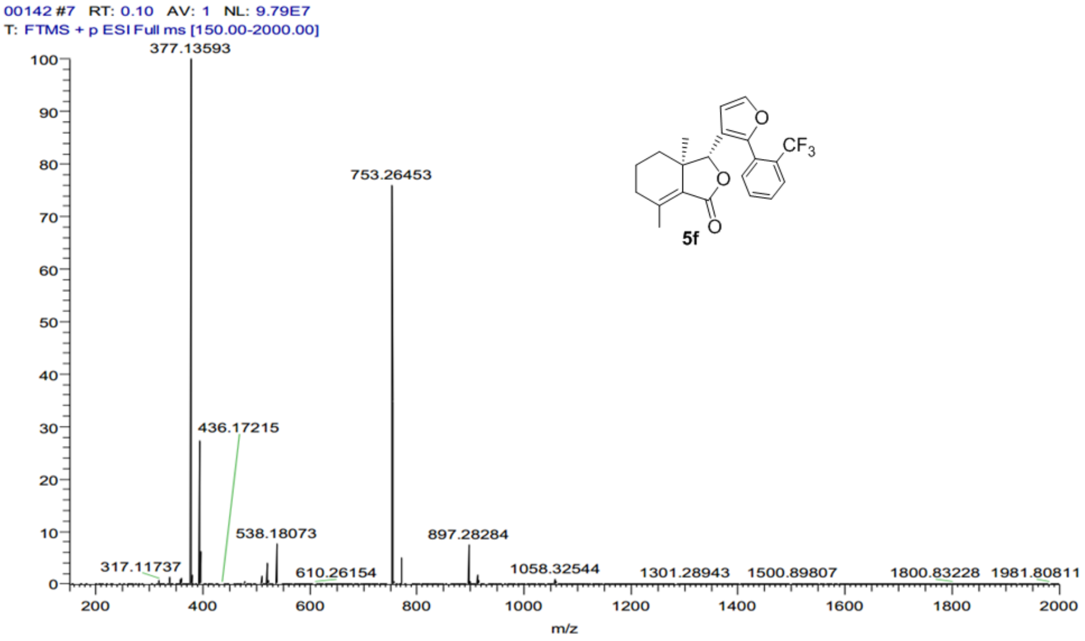

5g: HRMS (ESI) m/z calcd for $C_{17}H_{21}O_3^+$ $(M+H)^+$ 273.14852, found 273.14886.

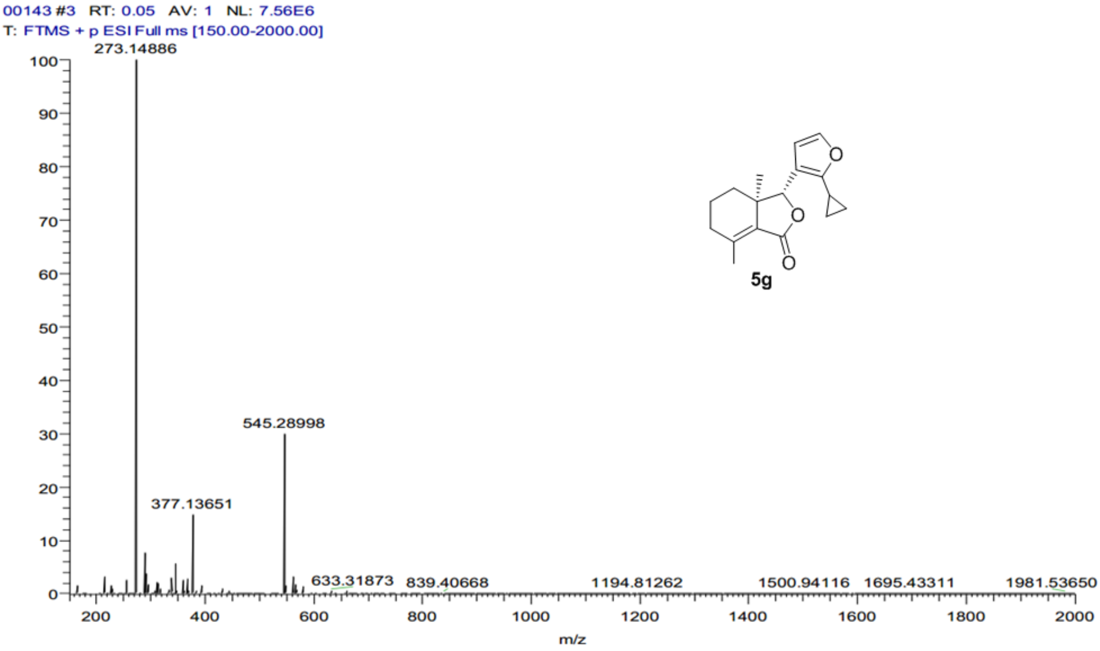

5h: HRMS (ESI) m/z calcd for $C_{21}H_{23}O_4^+$ $(M+H)^+$ 339.15909, found 339.15906.

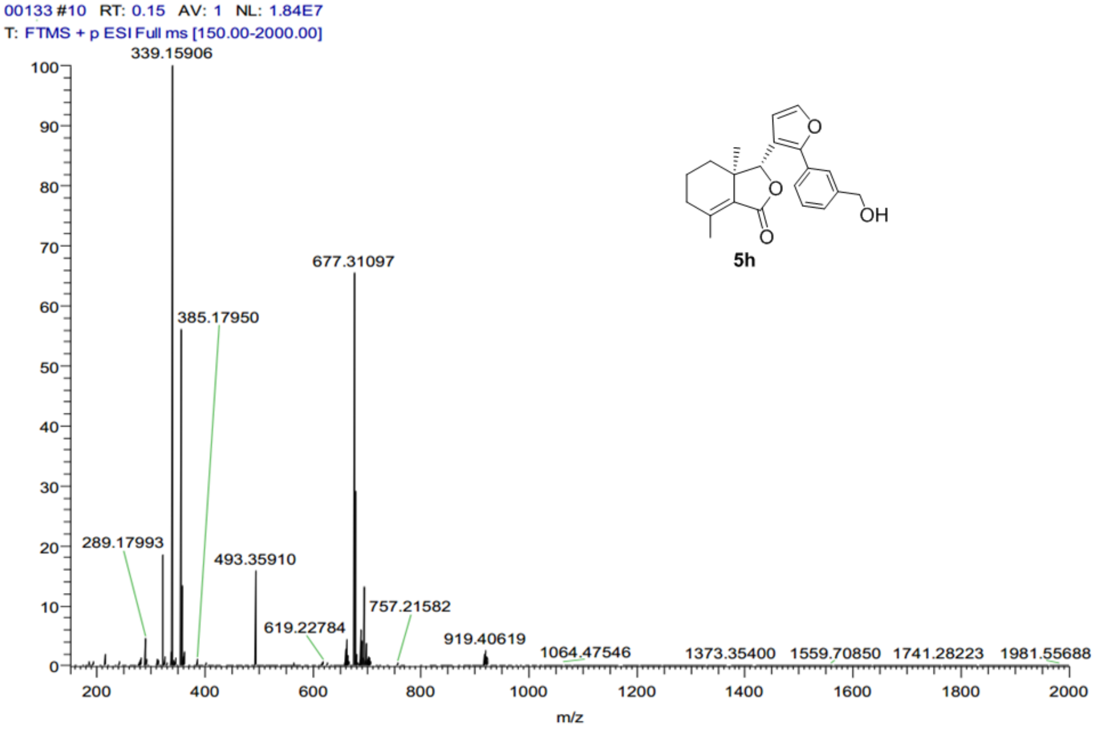

6a: HRMS (ESI) m/z calcd for $C_{26}H_{25}O_3^+$ $(M+H)^+$ 385.17982, found 385.17978.

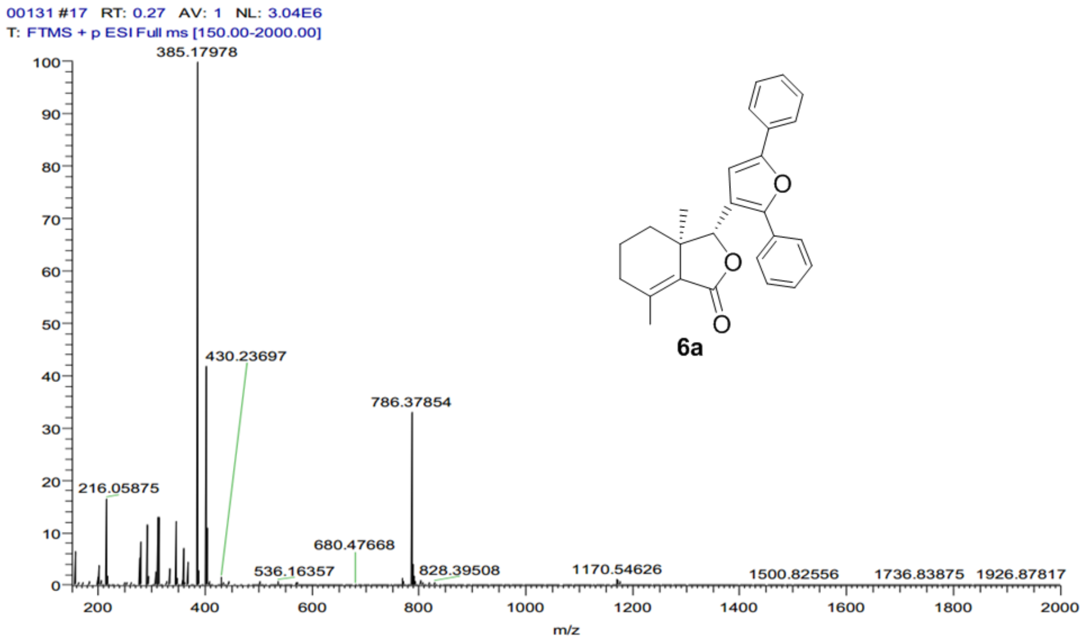

6b: HRMS (ESI) m/z calcd for $C_{22}H_{33}O_3^+$ $(M+H)^+$ 345.24242, found 345.24234.

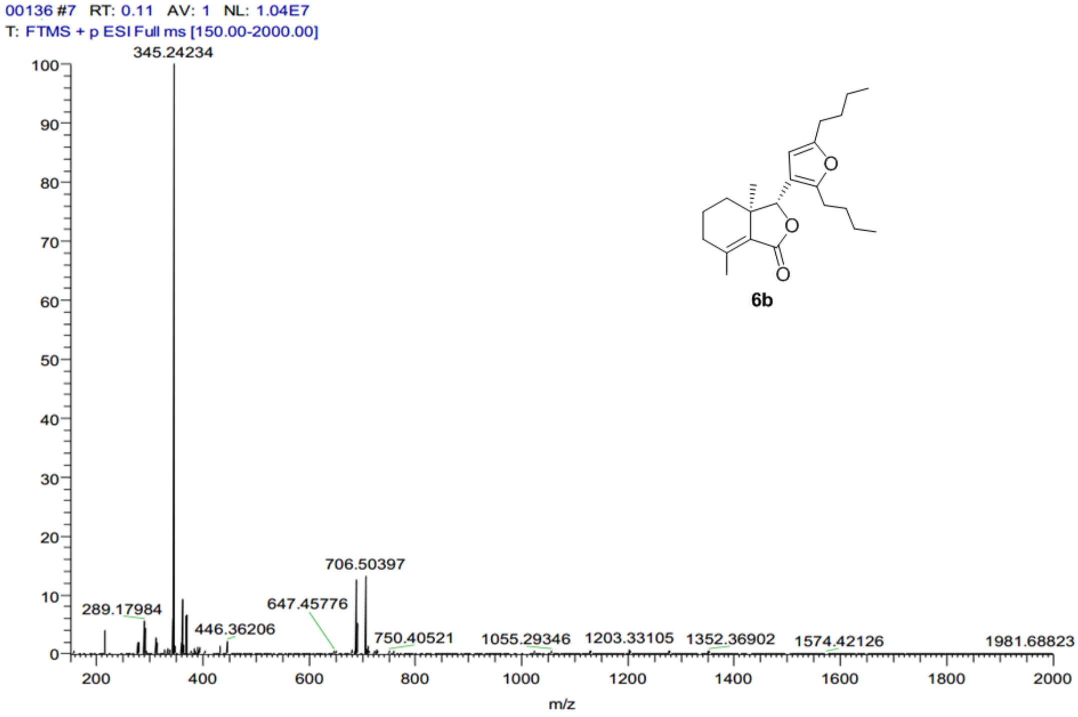

9: HRMS (ESI): m/z calcd for $C_{26}H_{31}O_{8}$ [M+H]+ 471.2013, found 471.2010.

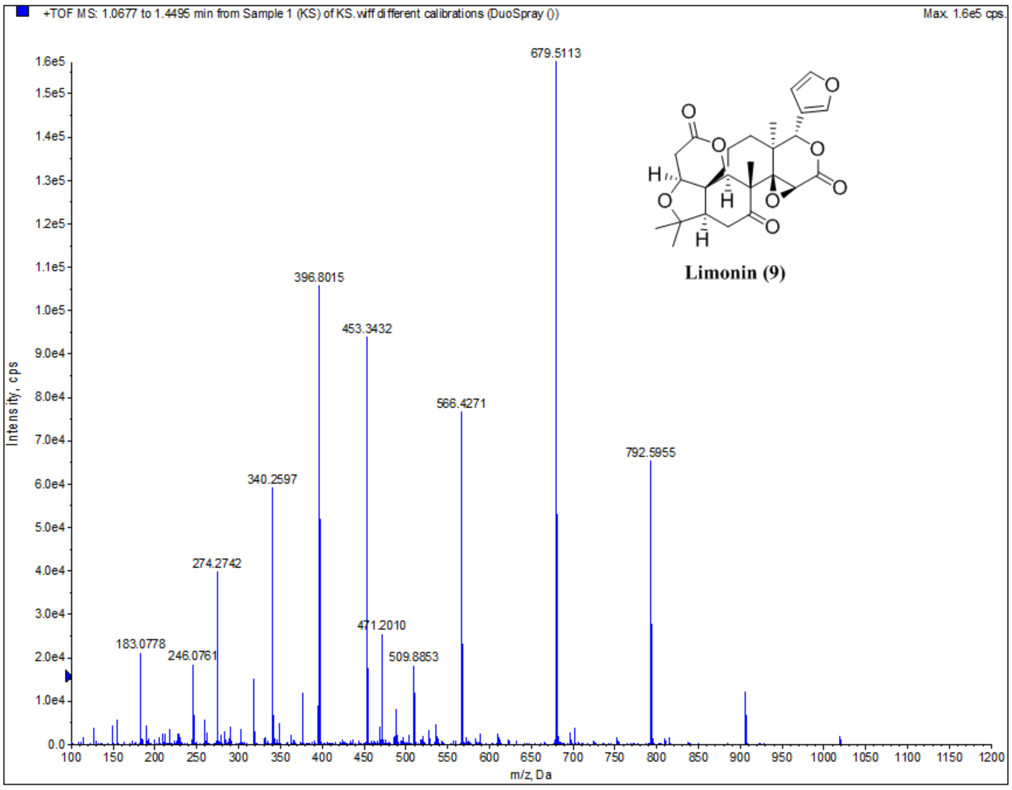

10: HRMS (ESI): m/z calcd for $C_{26}H_{30}O_{8}Br$ [M+H]+ 549.1119, found 549.1109.

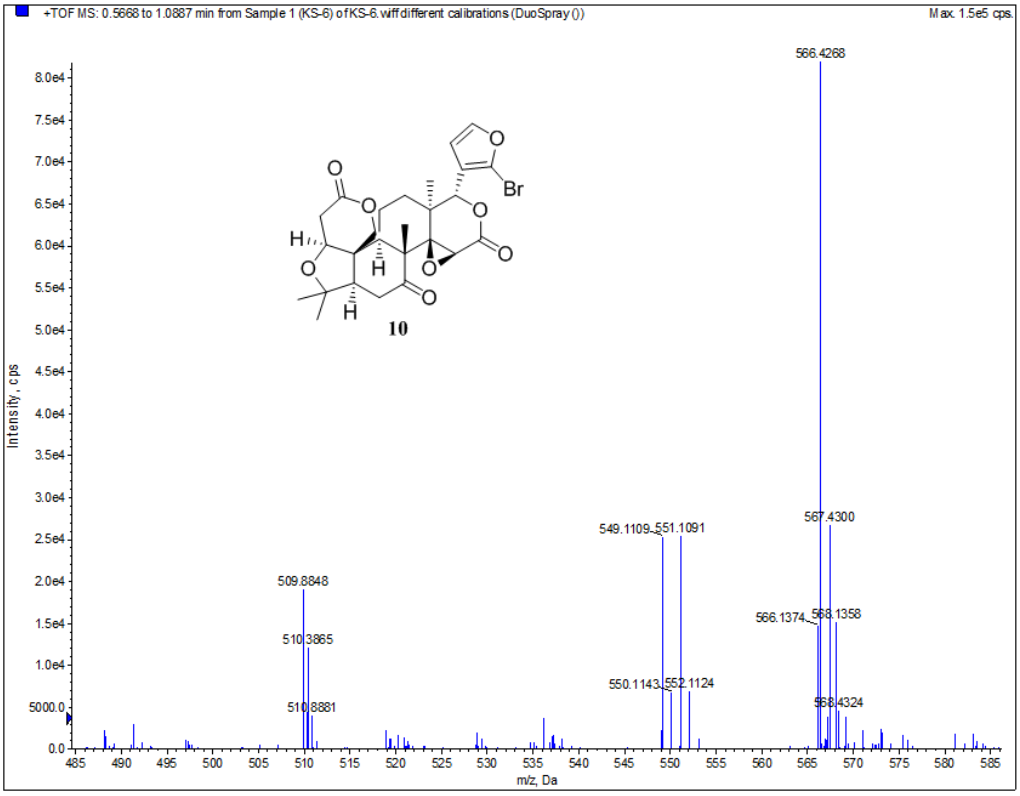

11: HRMS (ESI): m/z calcd for $C_{26}H_{29}O_8Br_2$ $[M+H]^+$ 627.0224, found $]^+$ 627.0214.

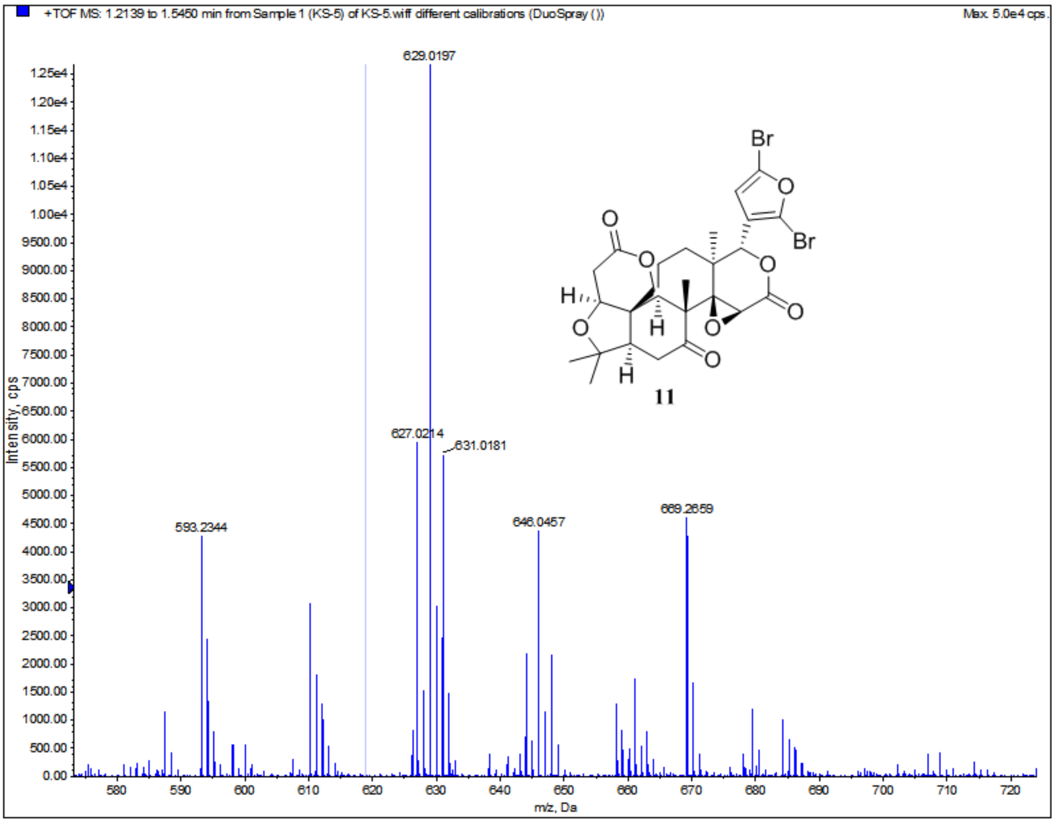

Supplement: Supplementary file 1 — Supplementary Information [file 41598_2018_26747_MOESM1_ESM.pdf]
